# DRN facilitates WUS transcriptional regulatory activity by chromatin remodeling to regulate shoot stem cell homeostasis in *Arabidopsis*

**DOI:** 10.1371/journal.pbio.3002878

**Published:** 2024-11-08

**Authors:** Linjie Luo, Li Liu, Lili She, Haoran Zhang, Nannan Zhang, Yaqin Wang, Yuting Ni, Fugui Chen, Fengying Wan, Yuqiu Dai, Guoping Zhu, Zhong Zhao

**Affiliations:** 1 Anhui Provincial Key Laboratory of Molecular Enzymology and Mechanism of Major Metabolic Diseases, Anhui Provincial Engineering Research Centre for Molecular Detection and Diagnostics, College of Life Sciences, Anhui Normal University, Wuhu, China; 2 CAS Center for Excellence in Molecular Plant Sciences, MOE Key Laboratory for Cellular Dynamics, School of Life Sciences, Division of Life Sciences and Medicine, University of Science and Technology of China, Hefei, China; University of California San Diego, UNITED STATES OF AMERICA

## Abstract

Shoot stem cells, harbored in the shoot apical meristem (SAM), play key roles during post-embryonic development of *Arabidopsis* and function as the origin of plant aerial tissues. Multiple transcription factors are involved in the sophisticated transcriptional regulation of stem cell homeostasis, with the *WUSCHEL* (*WUS*)/*CLAVATA3* (*CLV3*) negative feedback loop playing a central role. WUS acts as a master regulator in maintaining stem cells through its transcriptional regulatory activity including repressive and activating abilities. Although the interaction between WUS and TOPLESS confers the repressive activity of WUS in transcriptional control, the mechanism by which WUS activates gene expression remains elusive. Here, we showed that DORNRÖSCHEN competitively interacts with WUS and disturbs the WUS homodimer, which recruits BRAHMA to activate *CLV3* expression via nucleosome depletion for maintaining the stem cell pool.

## Abstract 2

DRN and WUS are known to upregulate the transcription of CLV3 in the shoot apical meristem of Arabidopsis, but how remains unclear. In this study, DRN is shown to interfere with WUS dimerization and chromatin remodeling to activate CLV3 transcription, thus contributing to the maintenance of the shoot stem cell pool.

## Introduction

Aboveground organs in plants, such as true leaves, flowers, and stems, originate from stem cells embedded in the shoot apical meristem (SAM) [1]. Stem cells also contribute to the developmental plasticity of plants to adapt to the ever-changing environment [2]. In *Arabidopsis*, the shoot stem cells, embedded in the central zone (CZ) of the SAM, divide slowly [3]. Some daughter cells retain the undifferentiated state of stem cells, while others differentiate into organ primordia through the periphery zone (PZ).

Previous studies have demonstrated that the *WUSCHEL* (*WUS*)*/CLAVATA3* (*CLV3*) negative feedback loop is a key hub of multiple regulatory networks underlying stem cell maintenance [4–7]. *WUS* transcribes in the organizing center (OC) beneath stem cells in the SAM. However, WUS proteins migrate to stem cells through the plasmodesmata, which maintain stem cells and directly activate the expression of *CLV3* [8–10]. *CLV3* encodes a peptide that acts as a signal to suppress *WUS* at the transcriptional and posttranslational levels via membrane receptors, *CLAVATA1* (*CLV1*)/*CLAVATA2* (*CLV2*)/*CORYNE* (*CRN*)/*BARELY ANY MERISTEMS* (*BAMs*)*/RECEPTORLIKE PROTEIN KINASE 2* (*RPK2*)/*CLAVATA3 INSENSITIVE RECEPTOR KINASES* (*CIKs*), for sustaining the stable pool of stem cells [11–14]. The robust activity of stem cells depends on the ability of WUS to positively and negatively regulate gene transcription, which relies on multiple domains of WUS [15–19]. A homeodomain (HD) of WUS can bind to DNA; a middle region is responsible for homodimerization; an acidic region and a WUS box are required for retention in the nucleus; and an EAR domain is related to nuclear export [9,20,21]. TOPLESS (TPL), a transcriptional corepressor, interacts with the WUS box and EAR domain, helping WUS to suppress gene expression [22,23]. However, the mechanism by which WUS activates downstream genes is unclear.

Interestingly, the ability of WUS to regulate gene expression seems to be closely related to the WUS protein concentration. Low levels of WUS proteins can activate *CLV3* expression, while high levels of WUS proteins function in the opposite way [24]. One mechanism underlying these effects could be that the WUS dimer and monomer, depending on the WUS concentration, have opposite effects on the regulation of *CLV3* expression. In this process, DNA *cis*-elements associated with WUS determine the threshold of WUS level for activating or repressing *CLV3* transcription [25]. Alternatively, WUS heterodimerization with co-regulators may play opposite roles in regulating downstream genes. Multiple WUS-interacting proteins have been identified in diverse developmental processes. During floral development, KNUCKLES (KNU) competitively interacts with WUS to expel it from the *CLV3* promoter, which ultimately inhibits *CLV3* expression and terminates the floral meristem (FM) [17]. In SAMs, the HAIRYMERISTEM (HAM) family proteins are expressed in the rib meristem (RM) and associated with WUS, which is important for stem cell homeostasis [26,27]. SHOOT MERISTEMLESS (STM) has also been reported to interact with WUS and bind to the *CLV3* promoter for stabilizing the DNA-protein complex to activate *CLV3* expression in SAMs [16]. Yet, how WUS initiates *CLV3* transcription is largely unknown.

Previous studies have shown that *DORNRÖSCHEN* (*DRN*) is mainly expressed in the CZ and directly up-regulates *CLV3* transcription independent of its DNA-binding activity [28,29], which suggests that DRN can interact with other proteins to jointly facilitate *CLV3* expression.

Here, we depicted a mechanistic framework for modulating *CLV3* expression by a protein complex including WUS, DRN, and BRAHMA (BRM). Chromatin remodeling processes by this complex positively regulate *CLV3* expression through specific nucleosome depletion, which depends on the unlocking of WUS-DRN anchored chromatin by SWItch/Sucrose Non-Fermentable (SWI/SNF) ATPases. Upon the specific relaxed DNA instead of nucleosome occupation, related transcription factors (TFs) including RNA polymerases are able to access the DNA and commence *CLV3* transcription to limit the stem cell population. This model provides a clear mechanism following the association between DNA and TFs, which is required for the positive transcriptional regulatory activity of WUS.

## Results

### DRN physically interacts with WUS and competitively inhibits WUS homodimerization

**Fig 1:**
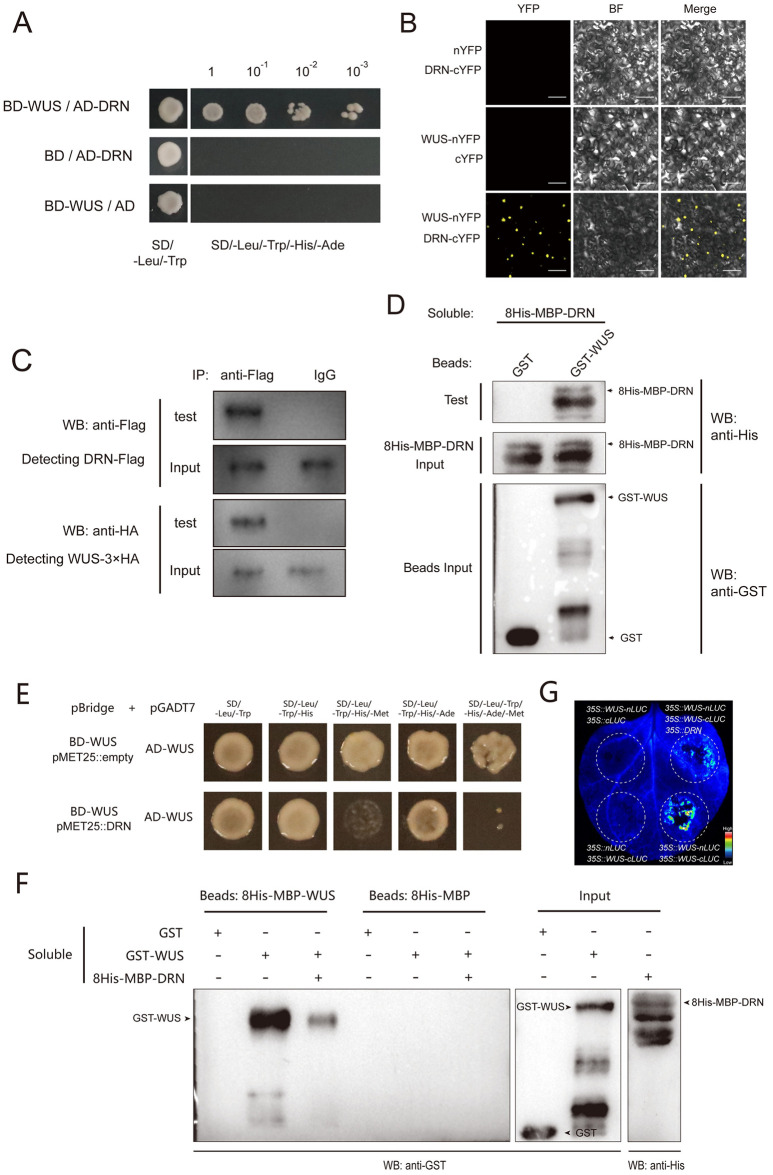
DRN competitively interacts with WUS. **(A)** Y2H assays exhibiting the interaction of WUS-DRN. BD and AD empty vectors were used as negative controls. Yeast cells were grown on the selective medium (SD/−Leu/−Trp/−His/−Ade) in a series of dilutions of 10^−1^, 10^−2^, and 10^−3^. Two independent experiments were performed with similar results. **(B)** BiFC exhibiting that the interaction of WUS-DRN occurs in tobacco leaves. BF, bright field. Scale bars, 100 μm. Five leaves were analyzed for each group. Two independent experiments were performed with similar results. **(C)** Co-IP showing the interaction of WUS and DRN in *Arabidopsis*. *35S*::*DRN-Flag* and *35S*::*WUS-3×HA* were transformed into *Arabidopsis* protoplasts. The anti-Flag antibody was used for IP. Instead of the anti-Flag antibody, IgG was used for IP as the negative control. Two independent experiments were performed with similar results. **(D)** Pull-down assays showing the interaction of WUS and DRN directly. Recombinant proteins were expressed in *E*. *coli*. Anti-His and anti-GST antibodies were used for immunoblot analysis. Two independent experiments were performed with similar results. **(E)** WUS–WUS interaction is disrupted by DRN in Y3H. Selective media (SD/−Leu/−Trp/−His) and (SD/−Leu/−Trp/−His/−Ade) were used to test the interaction of WUS-WUS. Selective media (SD/−Leu/−Trp/−His/−Met) and (SD/−Leu/−Trp/−His/−Ade/−Met) were used to test the competitive binding of WUS-DRN. Two independent experiments were performed with similar results. **(F)** Pull-down assays were used to determine the interruption of WUS homodimer by DRN. The binding group demonstrates the interaction of WUS-WUS, which is compromised by introducing 8His-MBP-DRN. Two independent experiments were performed with similar results. **(G)** Split-luciferase complementation assays in tobacco leaves were conducted to test that DRN competitively interacts with WUS. Five leaves were analyzed in each independent replicate. Two independent experiments were performed with similar results. BiFC, bimolecular fluorescence complementation; Y2H, yeast two-hybrid; Y3H, yeast three-hybrid.

WUS proteins are able to sustain stem cells and bind to the *CLV3* promoter to activate its transcription, which maintains stem cell homeostasis [8,9]. DRN can directly activate *CLV3* expression independent of its DNA-binding activity [29]. Furthermore, *WUS* and *DRN* are both required for shoot regeneration in tissue culture [30]. These findings suggest that WUS may interact with DRN to jointly regulate *CLV3* transcription and sustain stem cells. To test this hypothesis, we performed yeast two-hybrid (Y2H) assays and observed that WUS interacted with DRN in yeast cells with the positive control WUS-TPL and the negative control WUS-ARABIDOPSIS RESPONSE REGULATOR 7 (ARR7) (Figs 1A and S1). Additionally, bimolecular fluorescence complementation (BiFC) was also applied in tobacco (*Nicotiana benthamiana*) leaves, and we observed that WUS and DRN interacted with each other in vivo (Figs 1B and S2). To test this interaction in SAMs, we conducted BiFC assays in *Arabidopsis* SAMs using split YFP, driven by the native promoters of *WUS* and *DRN*, respectively. The fluorescent positive cells indicated that the WUS-DRN interactions occurred in SAMs (S3 Fig). Co-immunoprecipitation (co-IP) assays were also performed, and the immunoblot analysis showed that WUS-3×HA was co-immunoprecipitated with DRN-Flag using the anti-Flag antibody for IP, indicating the interaction of WUS and DRN in *Arabidopsis* (Fig 1C). To test whether the WUS-DRN interaction was direct, we performed pull-down assays with purified glutathione-S-transferase (GST)-WUS and 8His-maltose binding protein (MBP)-DRN expressed in *Escherichia coli* (*E*. *coli*), which confirmed our hypothesis (Fig 1D). Together, these data demonstrated that DRN physically interacts with WUS and may function as a co-activator in regulating *CLV3* expression.

Given that WUS contains multiple domains, we employed Y2H to determine which domain is responsible for the WUS-DRN interaction. The results exhibited that the middle region of WUS is required for the interaction with DRN (S4 Fig). With respect to DRN, the N-terminal part containing an AP2 domain (DNA-binding domain) accounts for the WUS-DRN interaction, but not the C-terminus (S5 Fig).

Previous studies have shown that WUS proteins can form homodimers via their middle region [9,24], which was also confirmed in our BiFC and Y2H assays (S6 Fig). The fact that the middle region of WUS contributes to both the WUS-WUS homodimers and WUS-DRN heterodimers suggests a hypothesis that the WUS-DRN interaction may compete with WUS homodimerization. To test this possibility, we carried out yeast three-hybrid (Y3H) assays. BD-WUS and AD-WUS were introduced into yeast along with the inducible DRN proteins driven by a conditional methionine promoter (*pMET25*). In the absence of methionine, *pMET25* promoter transcription occurred, and the induction of DRN proteins greatly impaired the WUS-WUS interaction (Fig 1E). These results indicated that DRN is able to disturb the WUS homodimerization in yeast. To exclude the effects of unrelated proteins in yeast cells, we performed pull-down assays with purified GST-WUS, 8His-MBP-WUS, and 8His-MBP-DRN, expressed in *E*. *coli*. These results, agreeing with those of Y3H, displayed that WUS dimerization was largely interrupted in the presence of DRN proteins through competitive binding in vitro (Fig 1F). In the competitive group of this pull-down experiment, the agarose beads with attached 8His-MBP-WUS proteins had been incubated with GST-WUS soluble proteins for 6 h before adding the 8His-MBP-DRN soluble proteins, which ruled out the possibility that the amount of free agarose beads loaded with 8His-MBP-WUS would decrease in the presence of 8His-MBP-DRN. In addition, split-luciferase complementation assays showed that DRN disrupted WUS homodimerization in vivo (Fig 1G). Collectively, our data demonstrated that DRN competitively interacts with WUS, which suggests that the WUS-DRN complex activates *CLV3* expression in the CZ.

### Direct activation of *CLV3* expression by WUS-DRN interactions

**Fig 2:**
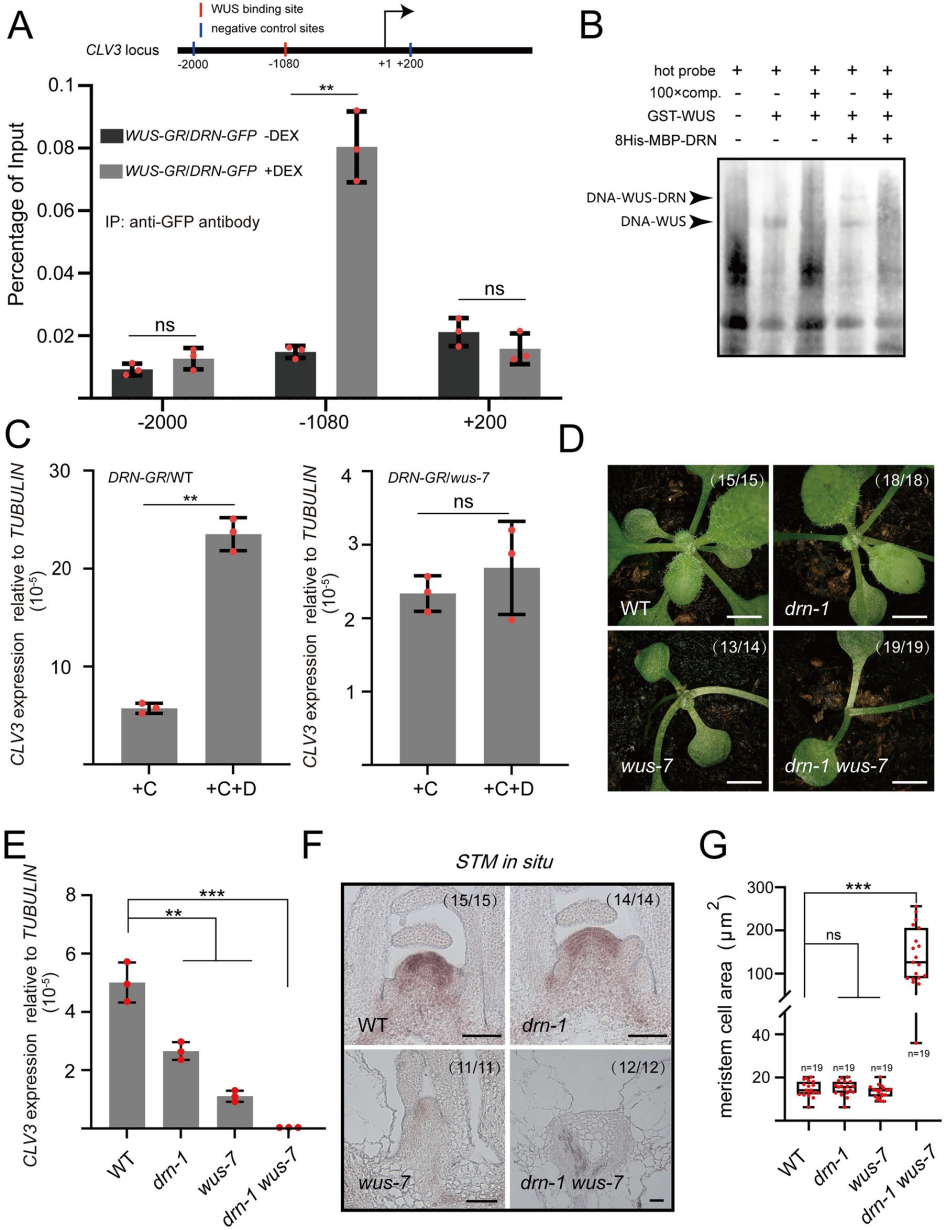
WUS-DRN complex associates with *CLV3* promoter to regulate *CLV3* expression. (A) The 14-day-old seedlings were used for ChIP assays. Upon DEX induction, the nuclear localization of WUS-GR enables the association of *CLV3* promoter (WUS-binding site, from −1,082 to −1,079) with DRN-GFP, using anti-GFP antibodies for IP. The upstream −2,000 bp site and downstream +200 site acted as negative control loci (no binding site). Two independent experiments were performed with similar results. **(B)** EMSAs results showing the direct binding of WUS-DRN and the *CLV3* promoter fragment enriched in ChIP. The black arrows indicate protein-DNA complexes. Two independent experiments were performed with similar results. **(C)** qRT-PCR was applied to test the relative transcript level of *CLV3* in *CLV3*::*DRN-GR*/WT and *CLV3*::*DRN-GR*/*wus-7* after DEX induction compared with the mock, using 14-day-old seedlings. +C, cycloheximide; +D, dexamethasone. Two independent experiments were performed with similar results. **(D)** Phenotypes of 14-day-old WT, *drn-1*, *wus-7*, and *drn-1 wus-7*. Scale bars, 1 mm. Two independent experiments were performed with similar results. **(E)** qRT-PCR was applied to test the relative *CLV3* expression in *drn-1*, *wus-7*, and *drn-1 wus-7* compared with WT, using 14-day-old seedlings. Two independent experiments were performed with similar results. **(F)** *STM* expression patterns were checked in SAMs of WT, *drn-1*, *wus-7*, and *drn-1 wus-7* by RNA in situ hybridization, using 14-day-old seedlings. Scale bars, 50 μm. Two independent experiments were performed with similar results. **(G)** Meristematic cell sizes of WT, *drn-1*, *wus-7*, and *drn-1 wus-7* in **F** were measured by image J software. Black bars, highest and lowest values; box, median 50%; black line in the box, median. Two independent experiments were performed with similar results. ***P < 0.001; **P < 0.01; ns, no significant difference; Student’s *t* test in **A**, **C**, **E**, and **G**. Data represent means ± SDs from 3 biological replicates in **A**, **C**, and **E**. The data underlying this figure can be found in S1 Data. ChIP, chromatin immunoprecipitation; DEX, dexamethasone; EMSA, electrophoretic mobility shift assay; SAM, shoot apical meristem.

To further explore the regulation of *CLV3* expression by the WUS-DRN complex, we generated transgenic plants, carrying *UBQ10*::*DRN-GFP* and *UBQ10*::*mCherry-WUS-GR* that produce dexamethasone (DEX) inducible WUS-GR. Chromatin immunoprecipitation (ChIP) was employed to determine the associations between DRN proteins and *CLV3* chromatin with or without DEX induction. The results showed that, upon nuclear localization of WUS-GR after DEX treatment, DRN strongly enriched the fragment of the *CLV3* promoter containing the WUS-binding site (TAAT, upstream from −1,082 to −1079), using the anti-GFP antibody for IP (Fig 2A). In contrast, DRN-GFP failed to effectively associate with the *CLV3* promoter without DEX induction (Fig 2A). However, why did the ChIP assays fail to detect the association of *CLV3* promoter with DRN in our previous study [29]? We reasoned that *DRN*::*DRN-GFP* lines, used for ChIP assays in the previous study [29], were in shortage of cells expressing WUS and DRN, and thus, extremely low abundance of DNA-WUS-DRN were immunoprecipitated. In the present study, we used materials with abundant DRN and inducible WUS, *UBQ10*::*mCherry-WUS-GR*/*UBQ10*::*DRN-GFP* lines, and succeeded in detecting the association of *CLV3* promoter by immunoprecipitation of DRN-GFP. These data indicated that DRN can access the *CLV3* promoter, which relies on WUS proteins in vivo. Previous studies have reported that multiple additional WUS-binding sites exist in downstream of *CLV3*, called the “*cis*-regulatory module” (CRM), and are involved in modulating *CLV3* expression [24,25]. We also performed ChIP to check the association of CRM DNA with the WUS-DRN complex. The results showed that CRM DNA cannot be co-immunoprecipitated with DRN-GFP in the presence of inducible WUS-GR (S7 Fig). We considered that WUS-DRN fails to associate with CRM DNA, which may be attributed to the low threshold of WUS level for WUS dimers in the CRM region [25]. To further investigate whether the WUS-DRN complex recognizes the fragment of the *CLV3* promoter with a WUS-binding site (upstream from −1,082 to −1,079), electrophoretic mobility shift assays (EMSAs) were conducted using the fragment of *CLV3* promoter (upstream from −1,090 to −1,064) as a probe. We observed a shift band in the presence of WUS, and an additional super-shift in the presence of both WUS and DRN (Fig 2B). Given that DRN cannot associate with the *CLV3* promoter alone [29], the ChIP and EMSA data in the present study suggested a regulatory model in which the activation of *CLV3* expression by DRN depends on WUS. To test this model genetically, we generated *CLV3*::*DRN-GR*/WT transgenic lines and introduced *CLV3*::*DRN-GR* into *wus-7*, a weak mutant allele of *WUS*, by crossing. Since the *wus* null mutants, in the absence of SAMs, fail to illustrate the genetic interaction on regulating SAM development, the weak mutant *wus-7* was selected for analysis (S8A Fig) [26,31]. After DEX induction, the *CLV3* expression of *CLV3*::*DRN-GR*/WT was significantly up-regulated compared with the control group (Fig 2C). However, the same DEX induction had no effect on *CLV3* transcription in *CLV3*::*DRN-GR*/*wus-7* (Fig 2C), which indicated that the activation of *CLV3* transcription by DRN-GR relies on *WUS*.

To further explore the biological significance of the WUS-DRN interaction, we analyzed *drn-1*, *wus-7*, and *drn-1 wus-7* mutants. The *drn-1* single mutants showed no obvious phenotypic defect in post-embryonic development, whereas the *wus-7* mutants grew slowly accompanied by a decrease of true leaves (Figs 2D and S8B–S8D). Interestingly, *drn-1 wus-7* double mutants showed severely arrested SAMs and fewer true leaves than *wus-7* single mutants, similar to *wus-8*, a null mutant allele of *WUS* (Figs 2D, S8A, S8E and S8F) [32]. The inflorescence meristem was also analyzed, and the *drn-1 wus-7* double mutants completely lacked their meristem, more severe than *drn-1* and *wus-7* single mutant (S9A and S9B Fig). The transcript level of *CLV3* significantly declined in the *drn-1* and *wus-7* single mutants, consistent with the activating roles of *DRN* and *WUS* (Figs 2E and S8J–S8L). However, the *CLV3* expression was barely detected in the *drn-1 wus-7* double mutant, similar to the *wus-8* null mutants (Figs 2E, S8M and S8N). Given the severe defects in true leaf formation in *drn-1 wus-7* double mutants, we speculated that the SAMs of the double mutants are completely dysfunctional. To test this hypothesis, RNA in situ hybridization was performed to examine the *STM* expression pattern in wild-type, *drn-1*, *wus-7*, and *drn-1 wus-7* plants, as *STM* is a well-recognized marker gene of the SAM [33]. The results showed that there was no significant difference in the region of *STM* expression in SAMs between *drn-1* and the wild type, whereas a decrease in *wus-7*, and *STM* RNA was barely detectable in *drn-1 wus-7* (Fig 2F). Additionally, it was exhibited that cells in the meristem region of *drn-1 wus-7* were much larger than those of wild-type, *drn-1*, and *wus-7* plants, indicating that these cells in *drn-1 wus-7* are highly differentiated rather than normally smaller meristematic cells (Fig 2F and 2G). We also generated *drn-1 wus-8* double mutants, showing similar phenotype to *drn-1 wus-7* and *wus-8* (S9A and S9B Fig), suggesting the role of DRN-WUS interaction in sustaining stem cells.

Since no obvious phenotypic defect in *drn-1* was observed, we also crossed the *drn-1 drnl-2* double mutant with *wus-7*. *DORNRÖSCHEN-LIKE* (*DRNL*) is a homolog of *DRN*, and they have been reported to redundantly modulate shoot stem cell homeostasis and organ initiation [29,34]. The *drn-1 drnl-2* double mutant showed an enlarged SAM, a decrease in the *CLV3* expression region (S8B, S8C, S8G, S8H, S8J, S8K, S8O and S8P Fig), as well as a pin-like inflorescence in the reproductive stage (S9A–S9C Fig) [29,34], whereas the SAM was absolutely absent in *drn-1 drnl-2 wus-7* triple mutants without subsequent development (S8I and S8Q Fig). Moreover, we generated *drn-1 clv3-7* and *wus-7 clv3-7* double mutants using *clv3-7*, a null allele of *CLV3* [5]. We found that there was no significant difference in SAM size between *drn-1 clv3-7* and *clv3-7* (S10A and S10B Fig). The SAM size of *wus-7 clv3-7* decreased obviously compared with *clv3-7*, and increased obviously compared with *wus-7*, indicating that *CLV3* negatively regulates *WUS* expression to maintain stem cell activity (S10A and S10B Fig). Together with the severe defects of SAMs in *drn-1 wus-7*, these data suggested that the WUS-DRN complex is not only involved in the regulation of *CLV3* expression, but also extensively participates in sustaining stem cells, and WUS seems to act as a master regulator in this process.

### DRN physically interacts with BRM

**Fig 3:**
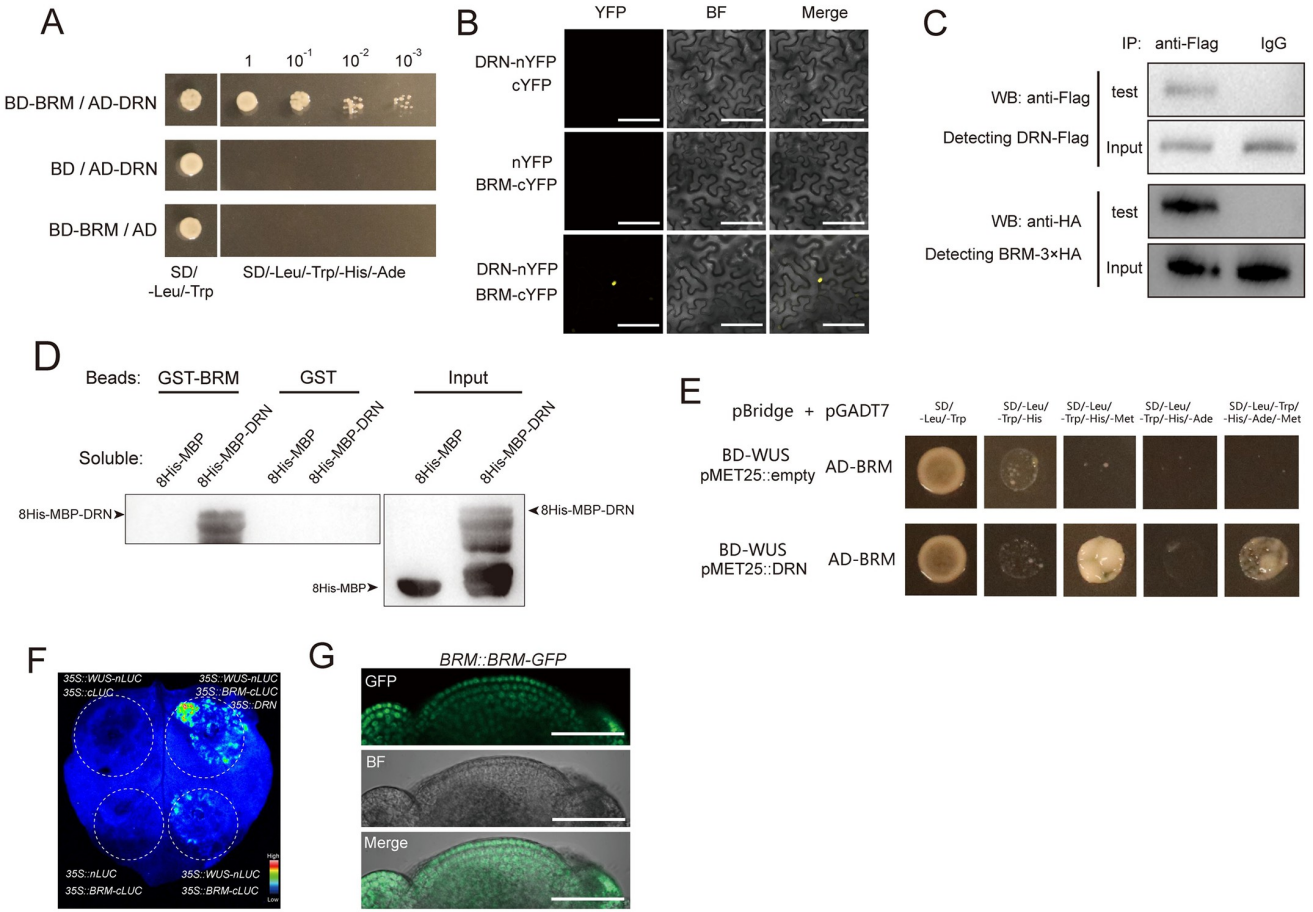
BRM interacts with DRN mediating WUS-DRN-BRM complex. **(A)** Y2H shows the interaction of DRN-BRM. The combinations with BD and AD empty vectors were introduced as negative controls. Yeast cells in a series of dilutions were grown on the selective medium (SD/−Leu/−Trp/−His/−Ade). Two independent experiments were performed with similar results. **(B)** BiFC exhibiting the interaction of DRN-BRM in tobacco leaves. YFP was split into N-terminus and C-terminus, which were fused to DRN and BRM, respectively. BF, bright field. Scale bars, 100 μm. Two independent experiments were performed with similar results, and 5 leaves were analyzed for each group. **(C)** Co-IP showing the interaction of DRN and BRM in *Arabidopsis*. *35S*::*DRN-Flag* and *35S*::*BRM-3×HA* were transformed into *Arabidopsis* protoplasts. The anti-Flag antibody was used for IP. Instead of the anti-Flag antibody, IgG was used for IP as the negative control. Two independent experiments were performed with similar results. **(D)** Pull-down assay showing the interaction of DRN and BRM directly. Recombinant proteins were expressed in *E*. *coli* and the anti-His antibody was used for immunoblot analysis. Two independent experiments were performed with similar results. **(E)** Y3H results showing that WUS fails to directly bind to BRM (upper lane) and that introducing DRN produces the WUS-DRN-BRM complex (bottom lane). SD/−Leu/−Trp/−His and SD/−Leu/−Trp/−His/−Ade were used for selective media. Lacking Met in addition allowed the transcription of *pMET25* promoter to produce DRN in the bottom lane but not in the upper lane. Two independent experiments were performed with similar results. **(F)** Split-luciferase complementation assays were used to test the association between WUS and BRM mediated by DRN in tobacco leaves. Two independent experiments were performed with similar results. Six leaves were analyzed for each independent replicate. **(G)** *BRM*::*BRM-GFP* lines were used to detect the distribution of BRM-GFP in SAMs. Green, GFP signal; BF, bright field. Scale bars, 50 μm. Eight apices were analyzed. BiFC, bimolecular fluorescence complementation; SAM, shoot apical meristem; Y2H, yeast two-hybrid; Y3H, yeast three-hybrid.

To further explore the mechanism underlying the activation of *CLV3* by WUS-DRN, we hypothesized that the WUS-DRN complex recruits additional proteins to facilitate the transcription of *CLV3*. To test this possibility, Y2H screening was employed to identify candidate proteins using BD-DRN as the bait. The results showed that BRM was identified as a candidate protein interacting with DRN and was independently isolated 3 times among 512 sequenced clones. BRM is a vital factor involved in the chromatin remodeling process to enable gene transcription [35–37], and mutating *BRM* leads to a decrease in SAM size [38]. Y2H assays were subsequently used to confirm the DRN-BRM interaction using the full-length coding sequences of *DRN* and *BRM* (Fig 3A). We also identified the N-terminus of BRM (amino acids 1–976), which is responsible for the interaction with DRN, using truncated BRM in Y2H assays (S11 Fig). We further adopted BiFC assays to verify DRN-BRM interactions in tobacco leaves (Figs 3B and S12). BiFC assays were also performed in *Arabidopsis* using *DRN*::*DRN-nYFP* and *BRM*::*BRM-cYFP* transgenic plants, which showed positive signals in the SAM, indicating that DRN-BRM interactions can occur in meristematic cells (S13 Fig). In addition, co-IP was applied to test this DRN-BRM interaction in *Arabidopsis* (Fig 3C). To further determine whether the DRN-BRM interaction is direct, pull-down assays were conducted, and the results showed a direct interaction between DRN and BRM in vitro (Fig 3D).

To test which region of the DRN protein is responsible for the interaction with BRM, truncated DRN proteins were used to perform Y2H assays. We observed that the C-terminus of DRN interacts with BRM (S14A and S14B Fig). Given that the DRN N-terminus interacts with WUS (S5 Fig), these data suggested that DRN may bridge the association between WUS and BRM and that the WUS-DRN-BRM protein complex may represent the molecular basis for WUS to activate *CLV3* expression. To further verify the protein interaction module of WUS-DRN-BRM, Y3H assays were performed using pBridge (BD-WUS, *pMET25*::*DRN*) and pGADT7 (AD-BRM) constructs. In the presence of methionine, the *pMET25* promoter was inhibited and WUS alone failed to interact with BRM (Fig 3E). When DRN proteins were expressed in yeast cells without methionine, the yeast reporter genes *HIS3* and *ADE2* were activated, indicating that the association between WUS and BRM relies on DRN (Fig 3E). In addition, split-luciferase assays showed that DRN functioned as a bridge connecting WUS and BRM in vivo (Fig 3F).

WUS and DRN proteins have been reported to be located and function in shoot stem cells [8,9,28,29]. *DRN*::*DRN-GFP/drn-1* and *WUS*::*WUS-GFP/wus-8* rescue lines were used to determine the distribution of DRN and WUS proteins in SAMs. The results showed that DRN and WUS proteins were present in shoot stem cells, whereas low levels of WUS were detected in L1 and L2 cell layers, and *CLV3*::*GFP/*WT lines were used to mark the shoot stem cells, as described in previous studies (S15A–S15C Fig) [5,8,10,29]. To carefully examine the expression pattern of *BRM* in the SAM, *BRM*::*BRM-GFP* lines were used and the results showed that BRM-GFPs occupied the whole SAM including the central zone, in line with a previous study [35] (Fig 3G). Specifically, abundant BRM-GFP proteins were located within the L1 cell layer of SAMs (S15D Fig). RNA in situ hybridization was also used to detect *BRM* mRNAs, which showed that *BRM* transcripts exist throughout the SAM, including in stem cells, but no signal in the sense probe control (S15E and S15F Fig). The presence of WUS, DRN, and BRM proteins in stem cells suggested that they may function in maintaining stem cells.

Collectively, DRN interacts with WUS and BRM proteins, forming a large protein complex mediated by DRN, which suggests a mechanism of regulating *CLV3* transcription.

### DRN and BRM jointly regulate *CLV3* transcription

**Fig 4:**
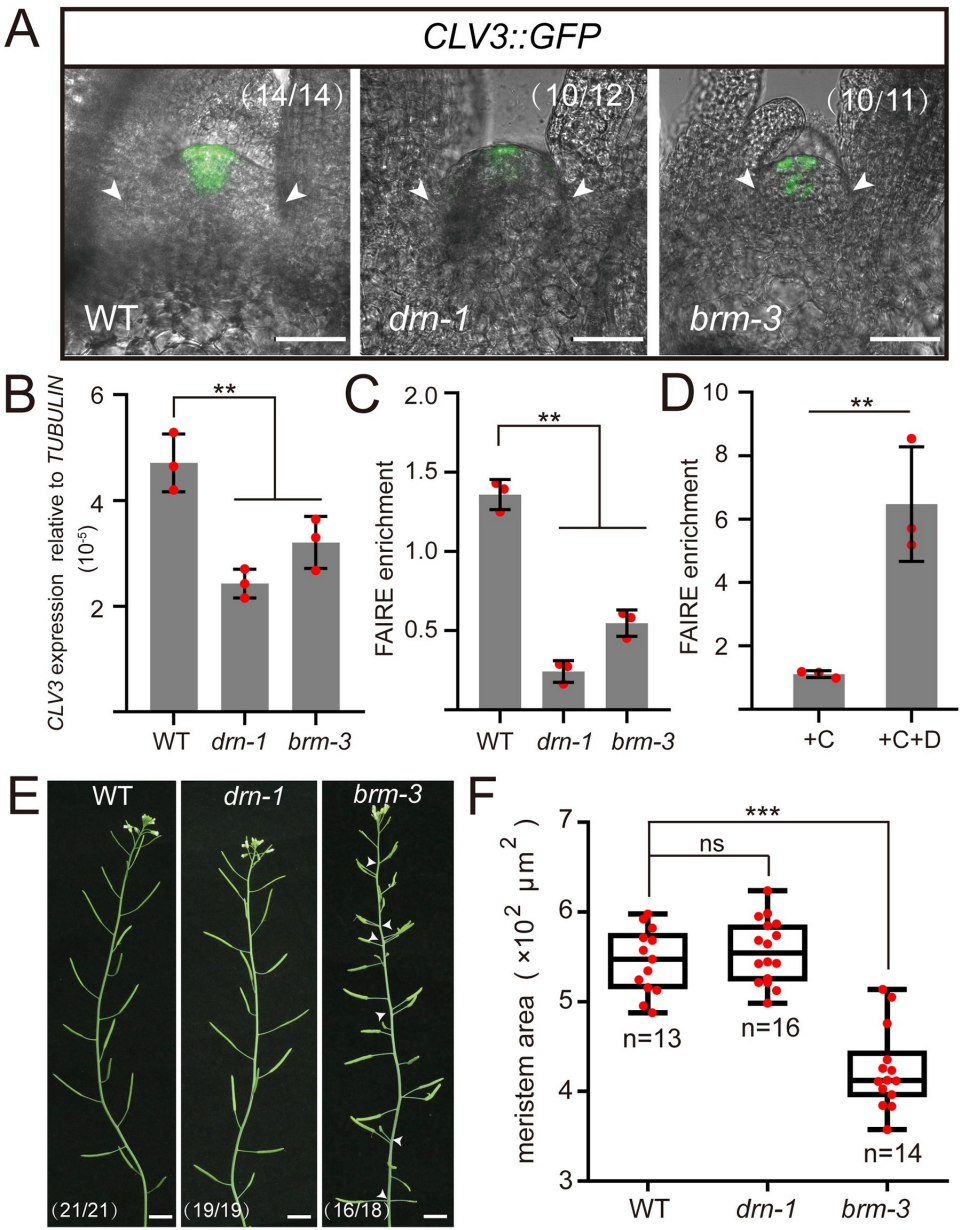
*BRM* contributes to *CLV3* expression and SAM maintenance. **(A)** *CLV3* expression was checked in WT, *drn-1*, and *brm-3* introduced with *CLV3*::*GFP*, using 14-day-old seedlings. The white arrows indicate the boundaries of SAMs. Scale bars, 50 μm. Two independent experiments were performed with similar results. **(B)** qRT-PCR was applied to test the relative *CLV3* transcript levels of *drn-1* and *brm-3* compared with WT, using 14-day-old seedlings. Two independent experiments were performed with similar results. **(C)** DNA accessibility at the *CLV3* locus was evaluated by FAIRE experiments in WT, *drn-1*, and *brm-3*, using 10-day-old seedlings. The ratio of FAIRE enrichment at the *CLV3* promoter (WUS-binding site −1,080) was normalized to the *Ta3* retrotransposon. Two independent experiments were performed with similar results. **(D)** DNA accessibility at the *CLV3* locus (WUS-binding site −1,080) was checked by FAIRE assays in *35S*::*DRN-GR*/WT after DEX treatment, using 10-day-old seedlings. The ratio of FAIRE enrichment at the *CLV3* promoter (WUS-binding site −1,080) was normalized to the *Ta3* retrotransposon. +C, cycloheximide; +D, dexamethasone. Two independent experiments were performed with similar results. **(E)** The inflorescences of WT, *drn-1*, and *brm-3* are shown. The white arrows indicate phyllotaxy defects. Scale bars, 1 cm. **(F)** The areas of SAMs in **A** were measured by image J and statistically analyzed. Black bars, highest and lowest values; box, median 50%; black line in the box, median. Two independent experiments were performed with similar results. ***P < 0.001; **P < 0.01; ns, no significant difference; Student’s *t* test in **B**, **C**, **D**, and **F**. Data represent means ± SDs from 3 biological replicates in **B**, **C**, and **D**. The data underlying this figure can be found in S1 Data. DEX, dexamethasone; FAIRE, formaldehyde-assisted isolation of regulatory elements; SAM, shoot apical meristem.

To further investigate the function of the WUS-DRN-BRM complex in regulating *CLV3* transcription, we analyzed *CLV3* expression in wild-type, *drn-1*, and *brm-3* plants by introducing *CLV3*::*GFP*. We observed that the transcriptional activity of *CLV3* promoter declined in *drn-1* and *brm-3* mutants compared with wild-type plants (Fig 4A). Consistently, qRT-PCR was also used to check *CLV3* transcripts, which demonstrated the markedly reduced *CLV3* expression in the *drn-1* and *brm-3* mutants compared with wild-type plants (Fig 4B).

To further confirm the regulation of *CLV3* expression by *BRM*, ethanol-inducible artificial microRNA*-BRM* (*amiBRM*) was introduced into wild-type plants. *CLV3* transcripts were subsequently examined by qRT-PCR in ethanol-inducible *amiBRM* lines, and the results showed that *CLV3* transcription was markedly reduced after 1% ethanol induction for 24 h, accompanied by the partial silencing of *BRM* transcripts (S16A and S16B Fig). These data indicated that both *DRN* and *BRM* contribute to the regulation of *CLV3* transcription.

Many studies have reported that chromatin remodeling complexes, such as SWI/SNF, are able to disrupt nucleosome structure and produce relaxed DNA, enabling the access of TFs, which eventually initiates gene transcription [35,37]. Considering that BRM is referred to as a key component in the SWI/SNF machinery in regulating gene transcription [36], we speculated that the WUS-DRN-BRM interactions might recruit SWI/SNF to the *CLV3* promoter, allowing specific nucleosome depletion to initiate *CLV3* transcription. To test this hypothesis, we performed formaldehyde-assisted isolation of regulatory elements (FAIRE) assays to examine the nucleosome-depleted regions (NDRs) in the *CLV3* promoter including the WUS-binding site (TAAT, from −1,082 to −1,079) in *drn-1* and *brm-3* mutants. In line with the reduced *CLV3* expression in these mutants, we observed the decline of NDRs enrichment in *drn-1* and *brm-3* mutants compared with the wild type, whereas no significant change was detected in −2,000 upstream site, as a negative control, indicating reduced DNA accessibility in these mutants (Figs 4C and S17A). In addition, FAIRE assays were also applied to *35S*::*DRN-GR*/WT lines. Upon DEX induction, we observed a higher level of NDRs located at the *CLV3* promoter, including WUS-binding site (TAAT, from −1,082 to −1,079), agreeing with the activation of *CLV3* expression by DEX treatment (Figs 2C and 4D, and S17B), but not found in *CLV3* downstream CRM region (S18 Fig). These data demonstrated that the WUS-DRN-BRM complex participates in nucleosome destabilization of the *CLV3* promoter, eventually allowing its transcription. In addition to disturbed *CLV3* expression, we also observed a decrease of SAM size and severely disturbed phyllotaxis in *brm-3* mutants but no significant change in *drn-1* mutants (Fig 4A, 4E, and 4F), suggesting additional functions of *BRM* in regulating SAM activity.

**Fig 5:**
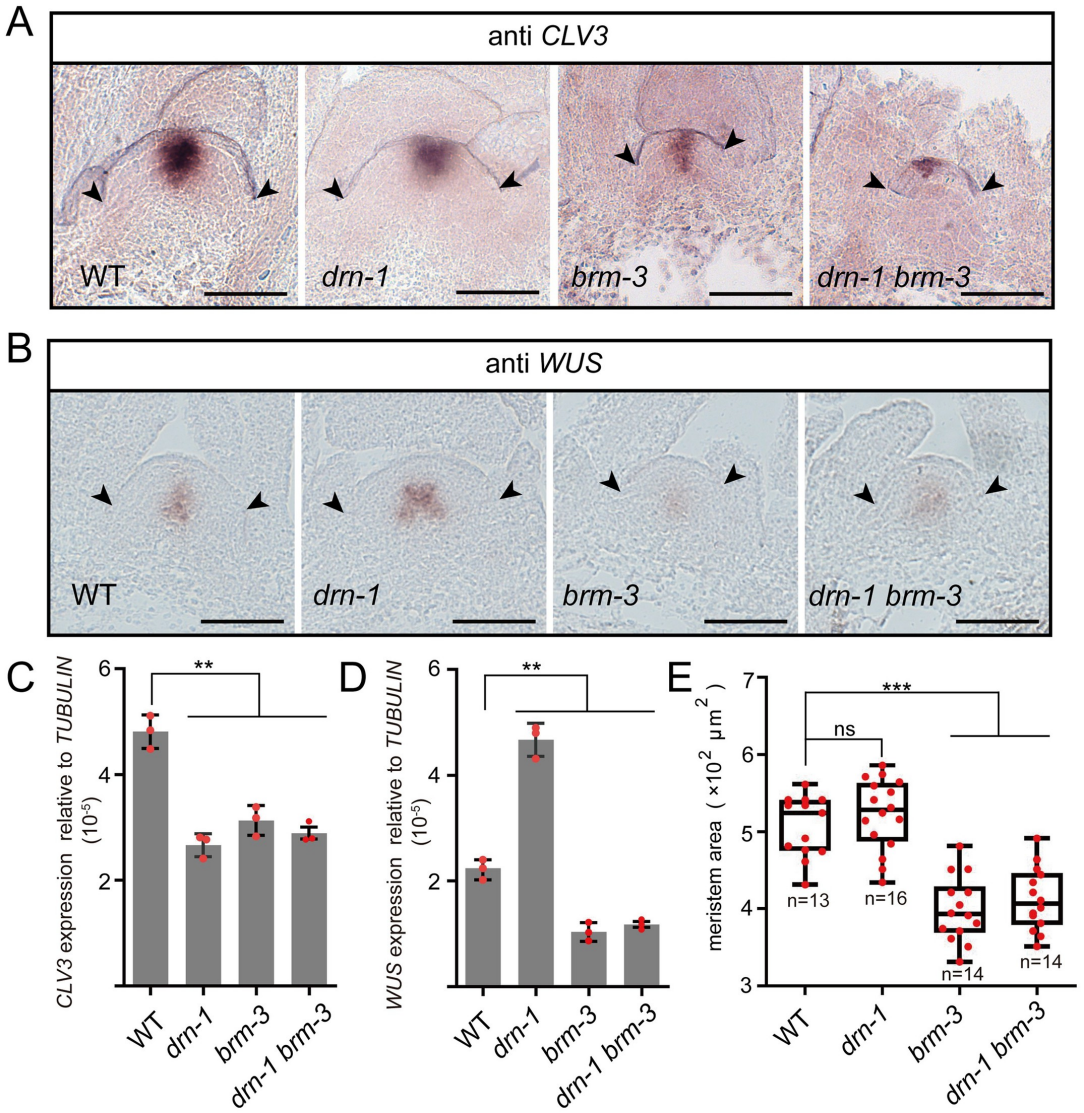
*BRM* is epistatic to *DRN* in regulating *WUS* transcription. **(A)** *CLV3* mRNAs were checked in SAMs of WT, *drn-1*, *brm-3*, and *drn-1 brm-3* by RNA in situ hybridization, using 14-day-old seedlings. Scale bars, 50 μm. Two independent experiments were performed with similar results, and 10 samples of each mutant were analyzed. **(B)** *WUS* mRNAs were checked in SAMs of WT, *drn-1*, *brm-3*, and *drn-1 brm-3* by RNA in situ hybridization, using 14-day-old seedlings. Scale bars, 50 μm. Two independent experiments were performed with similar results, and 10 samples of each mutant were analyzed. **(C)** qRT-PCR was applied to test the relative *CLV3* transcript levels of *drn-1*, *brm-3*, and *drn-1 brm-3* compared with WT, using 14-day-old seedlings. Two independent experiments were performed with similar results. **(D)** qRT-PCR was applied to test the relative *WUS* transcript levels of *drn-1*, *brm-3*, and *drn-1 brm-3* compared with WT, using 14-day-old seedlings. Two independent experiments were performed with similar results. **(E)** The areas of SAMs in **A** and **B** were measured by Image J software. Black bars, highest and lowest values; box, median 50%; black line in the box, median. Two independent experiments were performed with similar results. Black arrows indicate the boundaries of SAMs in **A** and **B**. **P < 0.01; ***P < 0.001; ns, no significant difference; Student’s *t* test in **C**, **D**, and **E**. Data represent means ± SDs from 3 biological replicates in **C** and **D**. The data underlying this figure can be found in S1 Data. SAM, shoot apical meristem.

To determine the genetic interaction between *DRN* and *BRM*, we generated *drn-1 brm-3* double mutants by crossing. Similar to single mutants, *CLV3* expression in *drn-1 brm-3* double mutants was decreased significantly compared with wild-type plants (Figs 5A, 5C, and S19). Despite decreased *CLV3* expression, *WUS* mRNAs were surprisingly reduced in *brm-3* and *drn-1 brm-3*, examined by RNA in situ hybridization, qRT-PCR, and the florescent reporter (Figs 5B, 5D, and S19), which agreed with the reduced SAM sizes of *brm-3* and *drn-1 brm-3* in both vegetative and reproductive development (Figs 5E, S20A and S20B). To exclude the possibility that overall developmental defects in *brm-3* affect SAM activity, we checked the number of true leaves of 14-day-old seedlings, showing no significant difference between the wild type and mutants (S21A and S21B Fig). Although *WUS* expression in *drn-1* increased moderately, that in *drn-1 brm-3* still decreased, similar to *brm-3*, indicating that *BRM* is epistatic to *DRN* in regulating *WUS* transcription (Fig 5D). In addition, we generated *brm-3 clv3-7* double mutants, which showed a decreased SAM size compared to *clv3-7* (S10A and S10B Fig). Together, we concluded that *BRM* not only regulates *CLV3* expression, but may also participate in additional signaling pathways to sustain SAM activity.

**Fig 6:**
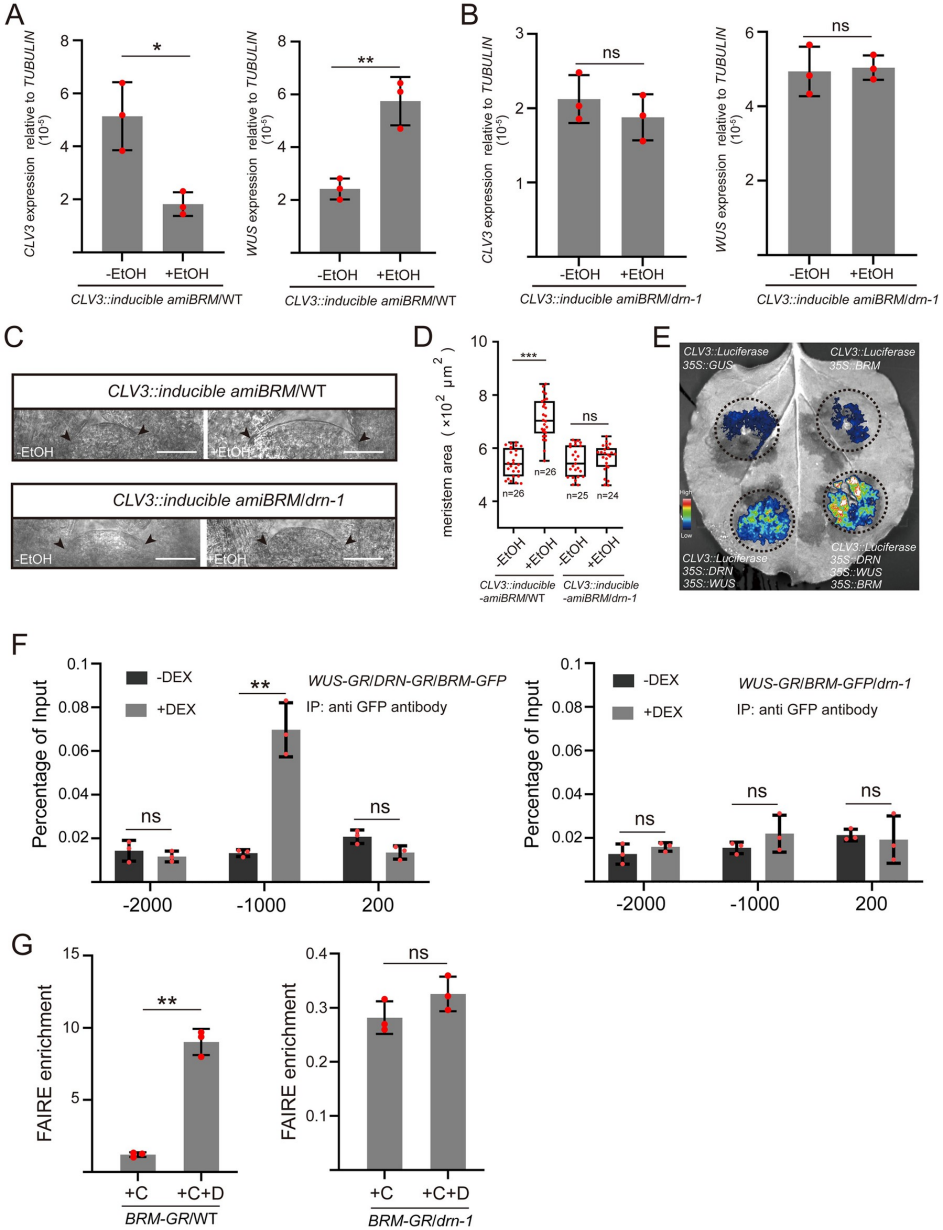
The positive regulation of *CLV3* by *BRM* relies on *DRN*. **(A)** qRT-PCR was applied to test the relative transcript levels of *CLV3* and *WUS* after ethanol (EtOH) induction for 24 h in *CLV3*::*inducible amiBRM*/WT transgenic seedlings (14-day-old). Two independent experiments were performed with similar results. **(B)** qRT-PCR was applied to test the relative transcript levels of *CLV3* and *WUS* after ethanol (EtOH) induction for 24 h in *CLV3*::*amiBRM*/*drn-1* transgenic seedlings (14-day-old). Two independent experiments were performed with similar results. **(C)** SAMs of the mock group and EtOH treated group (72 h) in *CLV3*::*inducible amiBRM*/WT and *CLV3*::*inducible amiBRM*/*drn-1* transgenic seedlings (14-day-old). Black arrows indicate the boundaries of SAMs. Bars, 50 μm. Two independent experiments were performed with similar results. **(D)** Areas of SAMs in **C** were measured by Image J software. Two independent experiments were performed with similar results. **(E)** Different combinations of vectors, including *CLV3*::*Luciferase*, *35S*::*GUS*, *35S*::*WUS*, *35S*::*DRN*, and *35S*::*BRM* were transformed into tobacco leaves via *Agrobacterium*. The intensity of luciferase signal represents the activity of *CLV3* promoter. The regions where *Agrobacterium* transformed were circled by dotted lines. Two independent experiments were performed with similar results. **(F)** *UBQ10*::*mCherry-WUS-GR/35S*::*DRN-GR/UBQ10*::*BRM-GFP* lines (14-day-old seedlings) were used for ChIP assays. Upon DEX induction, the nuclear localization of WUS-GR enables the association of the *CLV3* promoter (WUS-binding site, from −1,082 to −1,079) by BRM-GFP, using the anti-GFP antibody for IP, but not found in *UBQ10*::*mCherry-WUS-GR/UBQ10*::*BRM-GFP/drn-1* lines. The upstream −2,000 bp site and downstream +200 site acted as negative control loci (no binding site). Two independent experiments were performed with similar results. **(G)** DNA accessibility at the *CLV3* locus (WUS-binding site −1,080) was checked by FAIRE assays after DEX treatment, using *UBQ10*::*BRM-GR*/WT and *UBQ10*::*BRM-GR*/*drn-1* lines (10-day-old seedlings). The ratio of FAIRE enrichment at the *CLV3* promoter (WUS-binding site −1,080) was normalized to *Ta3* retrotransposon. +C, cycloheximide; +D, dexamethasone. Two independent experiments were performed with similar results. *P < 0.05; **P < 0.01; ***P < 0.001; ns, no significant difference; Student’s *t* test in **A**, **B**, **D**, **F**, and **G**. Data represent means ± SDs from 3 biological replicates in **A**, **B**, **F**, and **G**. The data underlying this figure can be found in S1 Data. ChIP, chromatin immunoprecipitation; DEX, dexamethasone; FAIRE, formaldehyde-assisted isolation of regulatory elements; SAM, shoot apical meristem.

Given the decrease of *WUS* expression in the *brm-3* mutant, we fail to rule out the likelihood that the decline in *CLV3* expression is a consequence of the reduced *WUS* levels in *brm-3*. To address this issue, we generated transgenic plants with ethanol-inducible *amiBRM* driven by the *CLV3* promoter (*CLV3*::*inducible amiBRM*) to specifically knock down *BRM* mRNAs in shoot stem cells. Upon ethanol induction for 24 h, RNA in situ hybridization results showed that *BRM* transcripts faded away in stem cells, but not in the OC and inner cells (S22 Fig). In *CLV3*::*inducible amiBRM*/WT plants, knocking down *BRM* in stem cells by ethanol induction for 24 h resulted into a decrease of *CLV3* transcription and, subsequently, an increase of *WUS* transcription (Fig 6A), whereas not occurring in the ethanol-inducible *GUS* control (S23A and S23B Fig). However, the *CLV3* and *WUS* transcription were not altered in *CLV3*::*inducible amiBRM*/*drn-1* plants (Fig 6B). Agreeing with the alteration of *CLV3* and *WUS* transcription, the SAM size noticeably increased in *CLV3*::*inducible amiBRM*/WT plants after ethanol treatment for 72 h, which was not found in *CLV3*::*inducible amiBRM*/*drn-1* plants and *GUS* negative control (Figs 6C, 6D, S23C and S23D). These data indicated that *BRM* regulates stem cell activity via modulating *CLV3* expression, which requires *DRN*. Furthermore, transient transfection of tobacco leaves was used to illustrate the synergistic effects of *WUS*, *DRN*, and *BRM* in regulating *CLV3* transcription. The results showed that the presence of *WUS* and *DRN* notably activated the activity of the *CLV3* promoter, whereas the luciferase signal of the group with *BRM* alone was comparable to the *GUS* control (Fig 6E). Introducing *WUS*, *DRN*, and *BRM* simultaneously resulted in much more intense signals (Fig 6E), indicating that the activation of *CLV3* expression by *BRM* relies on *DRN* and *WUS*.

In order to test the association between *CLV3* and BRM, we conducted ChIP assays using *UBQ10*::*WUS-GR*/*35S*::*DRN-GR*/*UBQ10*::*BRM-GFP* lines and *UBQ10*::*WUS-GR*/*UBQ10*::*BRM-GFP/drn-1* lines. We observed that BRM was able to bind to the *CLV3* promoter (−1,080 site) in the presence of DRN-GR and WUS-GR induced by DEX, which was not found in *drn-1* background (Fig 6F). We also performed FAIRE assays to test NDR levels using *UBQ10*::*BRM-GR* and *UBQ10*::*BRM-GR/drn-1* lines. The results showed that *BRM* overexpression could up-regulate the NDR level of *CLV3* promoter, but not in *drn-1* background, indicating that the modification of *CLV3* promoter chromatin by *BRM* requires *DRN* (Fig 6G). These data genetically demonstrated that *BRM* modulates *CLV3* expression by altering the chromatin state to tune *WUS*/*CLV3* feedback loop and maintain stem cell activity, which depends on *WUS* and *DRN*.

**Fig 7:**
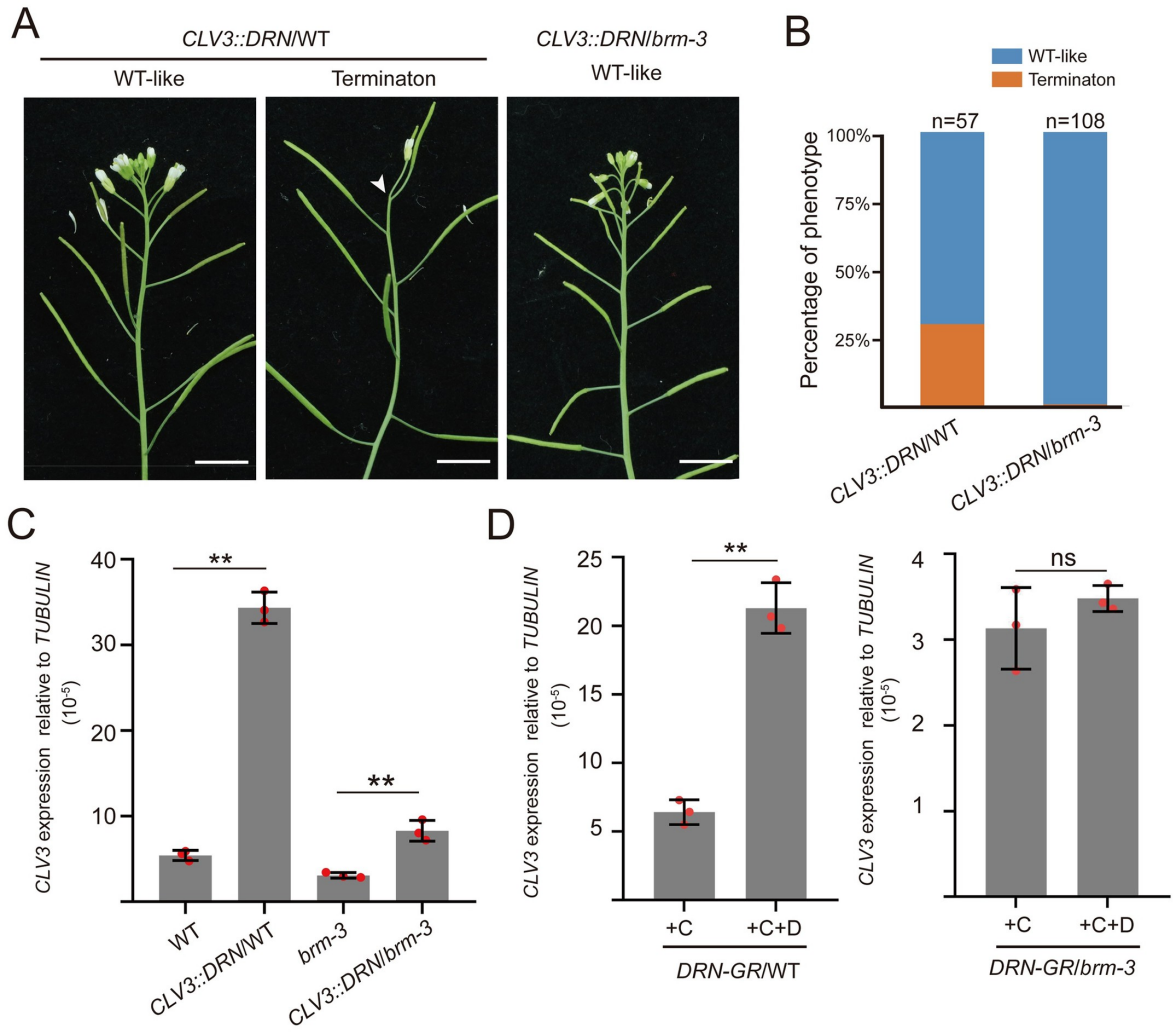
The activation of *CLV3* expression by *DRN* requires *BRM*. **(A, B)** The phenotypes of *CLV3*::*DRN* transgenic plants in WT and *brm-3* backgrounds are shown and statistically analyzed. The plants in **A** were analyzed in **B**. Two independent experiments were performed with similar results. **(C)** qRT-PCR was used to test the relative transcript level of *CLV3* in *CLV3*::*DRN/*WT, *brm-3*, and *CLV3*::*DRN/brm-3* lines, using 14-day-old seedlings. Two independent experiments were performed with similar results. **(D)** qRT-PCR was applied to test the relative *CLV3* transcript level of *CLV3*::*DRN-GR* in the wild-type background or *brm-3* mutant background after DEX induction compared with the mock group, using 14-day-old seedlings. +C, cycloheximide; +D, dexamethasone. Two independent experiments were performed with similar results. **P < 0.01; ns, no significant difference; Student’s *t* test in **C**, and **D**. Data represent means ± SDs from three biological replicates in **C** and **D**. The data underlying this figure can be found in S1 Data. DEX, dexamethasone.

To conversely determine whether the activation of *CLV3* expression by *DRN* required *BRM*, we generated 2 transgenic plants, *CLV3*::*DRN*/WT and *CLV3*::*DRN*/*brm-3*. Consistent with our previous study [29], *CLV3*::*DRN*/WT lines failed to produce SAMs, which was not found in *CLV3*::*DRN*/*brm-3* lines (Fig 7A and 7B). Moreover, qRT-PCR experiments were also performed on the 14-day-old seedlings of these 2 transgenic plants, and the results exhibited a dramatic increase in *CLV3* expression in *CLV3*::*DRN*/WT lines compared with wild-type plants (Fig 7C). However, the marked increase of *CLV3* transcription caused by *CLV3*::*DRN* was compromised in the *CLV3*::*DRN*/*brm-3* lines (Fig 7C), agreeing with the phenotypes of these 2 transgenic lines (Fig 7A and 7B), which suggests that the positive regulation of *CLV3* expression by *DRN* requires *BRM*.

To test whether the direct regulation of *CLV3* expression by DRN requires *BRM*, we introduced *CLV3*::*DRN-GR* into wild-type and *brm-3* mutant plants. After DEX induction, *CLV3* expression was significantly activated compared with the mock group in *CLV3*::*DRN-GR*/WT plants, whereas the *brm-3* mutation attenuated the up-regulation of *CLV3* expression induced by DEX in *CLV3*::*DRN-GR*/*brm-3* plants (Fig 7D). Taken together, our data demonstrated that DRN interacts with BRM and synergistically regulates *CLV3* expression depending on each other.

## Discussion

Stem cells embedded in the SAM generate almost all aerial organs of plants, which is critical for the success of plant life cycles and human sustenance. The *WUS*/*CLV3* feedback loop, known as a classical circuit in controlling the stem cell pool, has been extensively studied, and WUS has dual functions of modulating gene expression for stem cell activity [15]. Here, we showed that DRN competitively interacts with WUS and recruits BRM, located at the *CLV3* promoter, to activate its transcription via nucleosome depletion. This finding explains the mechanism by which WUS positively regulates gene transcription, whereas the negative transcriptional activity of WUS relies on the interaction with TPL [22,23]. Moreover, previous studies have reported that a dose-dependent manner confers the transcriptional regulatory ability of WUS [24]. WUS monomers, in the low levels of WUS proteins, tend to activate *CLV3* expression, whereas high levels of WUS result in WUS dimers to negatively regulate *CLV3* expression [24]. Our results showed that DRN competitively interacts with WUS to disturb the WUS dimer and recruits BRM to form the WUS-DRN-BRM complex, which uncovers the molecular base of WUS monomer activating *CLV3* expression. Yet, how the WUS dimer, with its homodimerization threshold is affected by CRM, represses *CLV3* expression remains elusive [24].

*CLV3*, acting as the marker gene of shoot stem cells, specifically transcribes in the CZ [5], which is under the precise control of multiple gene regulatory networks. WUS transcribes in the OC, and a fraction of WUS proteins move to stem cells to activate *CLV3* expression. However, a large portion of WUS proteins are retained in the OC and accumulate at high concentrations, which allow WUS homodimerization to repress *CLV3* expression [24]. Additionally, *HAM*s have been reported to be expressed in deep cell layers beneath stem cells and to repress *CLV3* expression [27]. Both WUS and HAMs are considered as the signals from the stem cell niche to modulate *CLV3* expression. Here, we also found that the transcription factor DRN, mainly located in stem cells, can positively regulate *CLV3* expression together with WUS, describing a new mechanism of restricting *CLV3* expression in shoot stem cells. Proper *CLV3* expression requires the synergetic regulation of signals from stem cells and stem cell niche. Furthermore, despite the reduced *CLV3* expression in *drn-1* and *drn-1 drnl-2* (Figs 4B, S8J, S8K, and S8P) [29], the SAM size of *drn-1* was comparable to the wild type, and *drn-1 drnl-2* does not have extremely enlarged SAMs like *clv3-7* (S9 and S10 Figs) [29], suggesting that additional unknown factors are involved in activating *CLV3* in stem cells.

WUS proteins have 2 functions in regulating shoot stem cell activity: activating *CLV3* and sustaining stem cell fate [4,6,8]. WUS interacts with DRN to activate *CLV3* expression to limit a stable stem cell pool. Our data also showed that the *drn-1 wus-7* double mutants completely lost their functional SAMs, more serious than either of the single mutants (Figs 2D, S9A and S9B), indicating the positive regulation of WUS-DRN in sustaining SAMs. Given that *WUS* is necessary for initiating shoot regeneration in tissue culture [39–41], our data may explain why *DRN* overexpression can also enhance shoot regeneration [42]. Similar to DRN and WUS, BRM also seems to have 2 opposite effects on modulating stem cells, activating *CLV3* and sustaining SAM activity. The *brm-3 clv3-7* mutants showed smaller SAMs than *clv3-7* (S10 Fig), whereas silencing *BRM* transcripts in stem cells led to decreased *CLV3* expression and increased SAM size (Fig 6A, 6C, and 6D). These results suggested that the WUS-DRN-BRM machinery may support stem cell activity via targeting additional unknown downstream genes, as well as limiting the stem cell number via activating *CLV3*.

We also found reduced *WUS* expression in *brm-3*, whereas *CLV3* expression likewise declined in *brm-3* (Figs 5A–5D and S19). These seemingly paradoxical results suggested that *BRM* not only regulates stem cell activity, but also sustains stem cell niche. The reduction in *CLV3* expression of *brm-3* is not sufficient to affect SAM size, as observed in the *drn-1* single mutant, and the decrease in *WUS* expression is predominantly responsible for the smaller SAM size in *brm-3* (Fig 5C–5E). Although BRM proteins are located in the whole SAM (Fig 3G), DRN proteins were not detected in OC (S15A Fig), indicating that the regulation of *WUS* expression by *BRM* does not rely on *DRN*.

**Fig 8:**
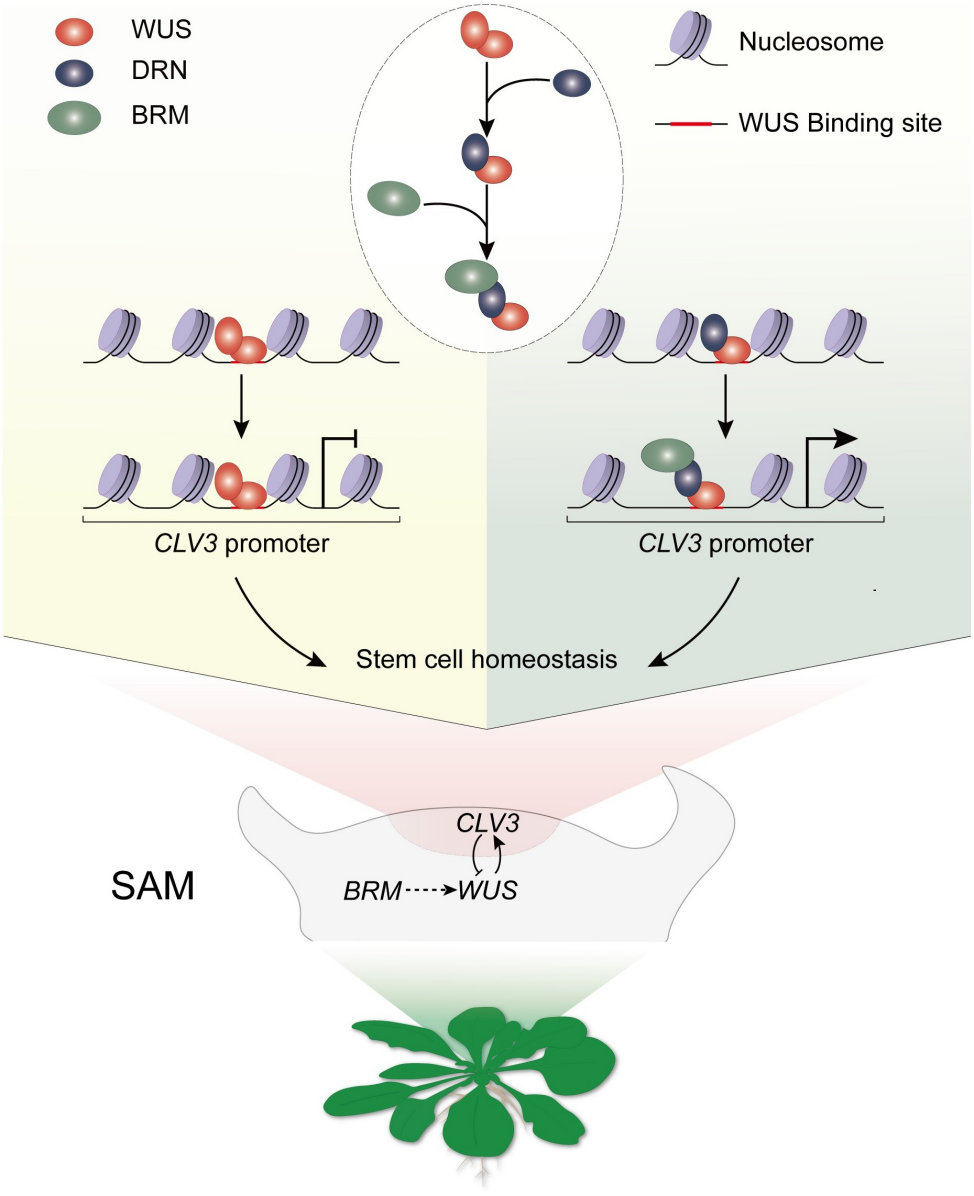
Schematic model of the WUS-DRN-BRM complex maintaining stem cell homeostasis. SAM, shoot apical meristem.

Overall, we illustrated a mechanism underlying the positive transcriptional regulatory activity of WUS, which relies on the WUS-DRN-BRM complex (Fig 8). This ternary complex plays a key role in regulating the *CLV3* transcription via chromatin remodeling process to maintain the stem cell pool (Fig 8). Further investigation is required to determine (1) the additional unknown downstream genes of the WUS-DRN-BRM complex in the maintenance of stem cell activity; and (2) the molecular basis underlying BRM regulates the stem cell niche.

## Methods

### Plant materials and treatments

All plants are Columbia-0 background. The *wus-8* (CS349353) is from ABRC [32]. The *wus-7* is a weak mutant allele of *WUS* [26,31] and was backcrossed with Columbia-0 for 5 times. The seeds of *brm-3* and *BRM*::*BRM-GFP* lines were kindly provided by Prof. Doris Wagner and Prof. Jun Xiao [35,37]. *clv3-7* is a null allele [5]. All seeds, except those used for the ethanol induction experiments, were sterilized in 70% ethanol and 0.5% Tween 20 for 10 min, and then washed twice with 95% ethanol. In the ethanol induction experiments, 0.16 M NaClO was used for sterilizing seeds, followed by several washes with water. Plants were grown under long-day conditions at 21°C. Half-strength Murashige and Skoog medium with 0.8% agar was used for cultivating the seedlings. For ethanol induction, we collected the inflorescence apices (*35S*::*AlcR AlcA*::*amiBRM*) and 14-day-old seedlings (*CLV3*::*AlcR AlcA*::*amiBRM*) with the induction condition of 1% ethanol for 24 h to analyze gene expression, for 72 h to analyze SAM size. In the DEX induction assays for gene expression analysis, 14-day-old seedlings were treated with 15 μM DEX and 50 μM cycloheximide in 1/2 MS medium for 2 h. For all induction assays, 3 biological replicates were performed.

### Plasmid construction

The 1.4 kb promoter and 1.3 kb terminator of the *CLV3* were used, respectively. *CLV3*::*DRN* and *CLV3*::*DRN-GR* were described in our previous study [29]. In Y2H assays, the full-length and truncated coding sequences of *WUS*, *DRN*, and *BRM* with stop codons were introduced into pGADT7 and pGBKT7 using EcoRI and BamHI sites. In pull-down assays, BRM1-976 and full-length coding sequence of *WUS* and *DRN* with stop codons were introduced into modified pET28b with an 8His-MBP tag using SalI and NotI sites. BRM1-976 and full-length coding sequence of *WUS* were introduced into pGEX6p-1 with a GST tag using BamHI and NotI sites. In co-IP assays, BRM1-976 and the full-length coding sequence of WUS and DRN without the stop codon were introduced into pUC19 with the Flag or 3×HA tag using SalI or BcuI site. We applied the gateway system to construct the plasmids used for BiFC assays in tobacco leaves. BRM1-976 and full-length coding sequence of *WUS*, and *DRN* without stop codon were introduced into entry vector PJLBlue, and were recombined into pGreen with the split YFP terminus by LR reaction. The 5.6 kb promoter and 1.2 kb terminator of the *WUS* [8], the 4.8 kb promoter and 1.5 kb terminator of the *DRN* [29], the 1.2 kb promoter and 0.5 kb terminator of the *BRM* [35], the full-length of *WUS*, *DRN*, and *BRM* CDS were used for BiFC in SAMs. The full-length of *BRM* CDS was used in *UBQ10*::*BRM-GR*. The target sequence of ethanol-inducible *amiBRM* was referred in Wu and colleagues [37]. Pre-amiRNA was assembled by PCR using the RS300 plasmid as templates. Subsequently, the PCR fragments were cloned after the *AlcA* promoter (*35S*::*AlcR AlcA*::*amiBRM*, *CLV3*::*AlcR AlcA*::*amiBRM*) in pGreen using gateway system. The primer sequences used in plasmid construction are listed in S1 Table.

### Chromatin immunoprecipitation (ChIP)

ChIP was performed as described in previous studies [29,43,44]. The entire seedlings (14-day-old) were inducted with 15 μM DEX for 2 h and fixed in fixation buffer (100 mM Na_3_PO_4_, 50 mM NaCl, 0.1 M sucrose, and 1% formaldehyde (pH 7)) under vacuum conditions 3 times for 10 min at room temperature. Subsequently, use glycine under vacuum for 5 min to end the fixation. The tissues were ground in liquid nitrogen. Nuclei were isolated from the tissues and resuspended with sonication buffer (10 mM Na_3_PO_4_, 100 mM NaCl, 0.5% sarkosyl, 10 mM EDTA, 1 mM PMSF, one complete protease inhibitor cocktail tablet per 10 ml (pH 7)). The chromatin was interrupted into fragments with the average DNA size of 0.2 to 1.0 kb using Bioruptor UCD-200 for sonication (30 s on, 30 s off, medium level, 15 min duration). The lysate was precleared by an incubation with 15 μl protein A beads (catalog no. 26162, Thermo Fisher) for 1 h and was incubated with the anti-GFP antibody (catalog no. Ab290, Abcam). The bound DNA was purified and analyzed by qPCR. The primers used for qPCR are shown in S1 Table.

### Co-immunoprecipitation (co-IP)

Plasmids were introduced into wild-type protoplasts. Total proteins were extracted from the protoplasts with the PEN-140 buffer (140 mM NaCl, 2.7 mM KCl, 25 mM Na_2_HPO_4_, 1.5 mM KH_2_PO_4_, 1 mM EDTA, 0.05% NP-40, 0.5 mM PMSF) and then incubated with Flag-Trap beads (catalog no. M20038, Abmart) at 4°C for 4 h (IgG group as a negative control), followed by 5 times of washing with PEN-140 buffer. Western blotting was performed to analyze the immunoprecipitated proteins using the anti-HA antibody (catalog no. M20003, Abmart) and the anti-Flag antibody (catalog no. M20008, Abmart).

### Pull-down

Recombinant GST-WUS, GST-BRM, 8His-MBP-WUS, and 8His-MBP-DRN proteins were expressed in *E*. *coli* BL21 (DE3) and purified; 1 ml well-cultured cells was added to 500 ml fresh lysogeny broth (LB) medium and cultured at 37°C until the optical density at 600 nm (OD600) reached 0.8 to 1. Proteins were induced by adding isopropyl-β-D-thiogalactopyranoside (IPTG) to a final concentration of 0.5 mM at 16°C for 16 h. The bacteria were harvested by centrifuging at 12,000 rpm. The cell precipitate was washed with buffer (100 mM NaCl, 50 mM Tris-HCl (pH 7.4)) 3 times and resuspended with buffer (100 mM NaCl, 50 mM Tris-HCl, 1 mM PMSF (pH 7.4)). After sonication (1 s on, 2 s off, at 30% amplitude for 10 min), the expressed GST-WUS and GST-BRM proteins were purified using glutathione resin (catalog no. P2253, Beyotime); 8His-MBP-WUS and 8His-MBP-DRN proteins were purified using Ni resin (catalog no. P2233, Beyotime). Beads loaded with proteins and soluble proteins were incubated by rotating at 4°C for 4 h in pull-down buffer (200 mM NaCl, 1 mM EDTA, 20 mM Tris-HCl, 0.15 mM PMSF (pH 8.0)). After being washed 3 times with pull-down buffer, the resin beads were boiled and processed for western blotting analyses using anti-GST (catalog no. M20025, Abmart) and anti-His (catalog no. M20020, Abmart) antibodies.

### Yeast two-hybrid (Y2H)

The pGBKT7 (BD) and pGADT7 (AD) vectors (Clontech) were used for plasmid construction. The combinations of AD and BD were transformed into the yeast strain Y2H Gold, which was cultured on complete medium lacking leucine and tryptophan (SD/−Leu/−Trp). The protein interactions were tested on complete medium lacking leucine, tryptophan, histidine, and adenine (SD/−Leu/−Trp/−His/−Ade). The empty BD and empty AD were used as negative controls.

### Yeast three-hybrid (Y3H)

The vectors of pBridge and pGADT7 were used for constructing plasmids. The full-length coding sequence of *WUS* and *DRN* were cloned behind the BD and *pMET25* promoter of pBridge, respectively. *WUS* and *BRM* full-length coding sequence were cloned into pGADT7 vector, respectively. The designed combinations of pBridge vectors and pGADT7 vectors were cotransformed into the yeast strain Y2H Gold. The interactions relationship among the 3 proteins were measured on selective medium (SD/-Leu/-Trp/-His, SD/-Leu/-Trp/-His/-Met SD/-Leu/-Trp/-His/-Ade, and SD/-Leu/-Trp/-His/-Ade/-Met).

### Bimolecular fluorescence complementation (BiFC)

The constructs were transformed into the *Agrobacterium*. The *Agrobacterium* were cultured, collected, and resuspended in infiltration buffer (10 mM MES, 10 mM MgCl_2_, 0.15 mM acetosyringone (pH 5.8)). Equal volumes of different *Agrobacterium* with YFP N-terminus or C-terminus fused proteins were mixed and incubated for 2 h at room temperature, subsequently injected into *N*. *benthamiana* leaves. The plants with injected leaves were grown in the green house for 48 h. The *DRN*::*DRN-nYFP*/*WUS*::*WUS-cYFP* and *DRN*::*DRN-nYFP*/*BRM*::*BRM-cYFP* lines were generated by crossing. YFP fluorescence was detected by confocal laser-scanning microscope (ZEISS, LSM710).

### Split-luciferase complementary assays

The constructs were transformed into the *Agrobacterium*. The *Agrobacterium* was cultured, collected, and resuspended in infiltration buffer (10 mM MES, 10 mM MgCl_2_, 0.15 mM acetosyringone (pH 5.8)) and adjusted to the OD = 1. Equal volumes of different *Agrobacterium* with luciferase N-terminus or C-terminus fused proteins were mixed and incubated for 2 h at room temperature, and subsequently injected into *N*. *benthamiana* leaves. The injected plants were cultured under normal conditions for 48 h; 1 mM D-Luciferin (catalog no. 40902ES01, YEASEN) was infiltrated into leaves, which were subsequently incubated for 3 min after injection. The luciferase activity was detected by an imaging system (Tanon, 5200).

### Transient activation assays in tobacco

The *Agrobacterium* with constructs were cultured, collected, and resuspended in infiltration buffer (10 mM MES, 10 mM MgCl_2_, 0.15 mM acetosyringone (pH 5.8)) and adjusted to OD = 1. The *Agrobacterium* carrying different constructs were injected into *N*. *benthamiana* leaves with identical *Agrobacterium* concentrations in all groups. The injected plants were grown under normal conditions for 48 h. Subsequently, 1 mM D-Luciferin (catalog no. 40902ES01, YEASEN) was injected into leaves, which were subsequently incubated for 3 min after injection. The luciferase activity was detected by an imaging system (Tanon, 5200).

### Electrophoretic mobility shift assay (EMSA)

The GST-WUS and 8His-MBP-DRN proteins were expressed in *E*. *coli* BL21 (DE3) and purified using Ni resin (catalog no. P2233, Beyotime). Size-exclusion chromatography was used to yield proteins with the desired molecular weight. The biotin-labeled probes were synthesized by Tsingke Biotechnology Co. The experiments were performed using the EMSA kit (catalog no. 20148, Thermo).

### Quantitative reverse transcription PCR (qRT-PCR)

The leaves and roots of 14-day-old seedlings were removed. Seedlings (0.05 g) were ground into powder in liquid nitrogen. RNA isolater Total RNA Extraction Reagent (catalog no. R401-01, Vazyme) was used to extract total RNA from the plant samples. The HiScript III RT SuperMix Kit (catalog no. R323-01, Vazyme) was used for cDNA synthesis. The sequences of primers used for qRT-PCR are listed in S1 Table. qRT-PCR was performed using a Roche LightCycler 96 instrument with SYBR qPCR Master Mix (catalog no. Q711-02, Vazyme) and the following PCR program: Step 1, 95°C for 5 min; Step 2, 40 cycles of 95°C for 10 s followed by 62°C for 30 s; and Step 3, 20°C for 10 s. *TUBULIN* was used for normalization. Three biological replicates were performed.

### Confocal microscopy

For SAM images of the *CLV3*::*GFP*, *WUS*::*GFP*, *WUS*::*WUS-GFP*, *DRN*::*DRN-GFP*, and *BRM*::*BRM-GFP* lines, the tissues were fixed with agarose and sectioned into 50 μm slices. Afterwards, the sections were visualized by a confocal microscope (ZEISS, LSM710). The top view of the *BRM*::*BRM-GFP* lines was performed by the confocal microscope (Olympus, FV3000), after the lateral floral organs were dissected and stained with propidium iodide (PI).

### RNA in situ hybridization

Templates of RNA probes were amplified from cDNA using gene-specific primers containing T7 promoter sequences at the 5′ end. The primer sequences are listed in S1 Table. The RNA probes with DIG-labeled UTP were synthesized by T7 RNA polymerase, and RNA in situ hybridization was performed according to standard protocols [29]. The RNA probes were detected by the anti-DIG antibody (catalog no. 11093274910, Roche).

### Formaldehyde-assisted isolation of regulatory elements (FAIRE) assays

FAIRE was performed as described [45]. For each replicate, 0.1 g of 10-day-old seedlings (removed leaves and roots) were crosslinked with crosslinking buffer (1% formaldehyde, 400 mM sucrose,10 mM Tris-HCl, 5 mM β-mercaptoethanol, 0.1 mM PMSF (pH 8.0)) under vacuum for 5 min, and 0.1 g materials was under vacuum for 5 min in crosslinking buffer without formaldehyde. The isolated DNA fragments were purified with Phenol/Chloroform/Isoamyl alcohol (25:24:1) for 2 times. The purified DNA was used as templates for qPCR. Primer sequences are listed in S1 Table. The *Ta3* retrotransposon (*At1g37110*) was used as a reference for normalization [46–48]. The fold enrichment of a locus was obtained by normalization to *Ta3* in crosslinked samples over that in uncrosslinked samples.

## Supporting information

S1 Table Oligonucleotides used in this study. (DOCX)

S1 Fig The controls in Y2H for detecting the WUS interaction. Yeast cells were grown on the selective medium (SD/−Leu/−Trp/−His/−Ade) in a series of dilutions of 10^–1^, 10^–2^, and 10^–3^. ARR7 and TPL served as the negative and positive controls, respectively. Two independent experiments were performed with similar results.
(TIF)

S2 Fig The negative controls in BiFC for detecting the interaction of WUS and DRN. ARF1 was used as the negative controls in BiFC. YFP was split into the N-terminus and C-terminus, fused to WUS, ARF1, and DRN. Scale bars, 100 μm.
(TIF)

S3 Fig The interaction of DRN-WUS in SAMs. The *DRN::DRN-nYFP/WUS::WUS-cYFP* transgenic plants were used to detect DRN-WUS interactions in inflorescence SAMs. *DRN::nYFP* and *WUS::cYFP* were introduced as negative controls. Scale bars, 50 μm. Two independent experiments were performed with similar results.
(TIF)

S4 Fig Identification of the region of WUS accounting for the interaction with DRN. (A) Diagram of the *WUS* coding sequence. (B) Truncated WUS and full-length DRN were used for Y2H. BD and AD empty vectors were introduced as negative controls. Yeast cells were grown on the selective medium (SD/−Leu/−Trp/−His/−Ade) in a series of dilutions of 10^–1^, 10^–2^, and 10^–3^. The experiments were independently performed two times with similar results.
(TIF)

S5 Fig Identification of the region of DRN accounting for the interaction with WUS. (A) Diagram of *DRN* coding sequence. (B) The full-length WUS, truncated and full-length DRN were used for Y2H. BD and AD empty vectors were used as negative controls. Yeast cells were grown on the selective medium (SD/−Leu/−Trp/−His/−Ade) in a series of dilutions of 10^–1^, 10^–2^, and 10^–3^. The experiments were independently performed 2 times with similar results.
(TIF)

S6 Fig Homodimerization of WUS. (A) BiFC exhibiting that the interaction of WUS-WUS in tobacco leaves. YFP was split into the N-terminus and C-terminus, fused to WUS, respectively. Scale bars, 100 μm. (B) The full-length and truncated WUS were used for Y2H. Yeast cells were grown on the selective medium (SD/−Leu/−Trp/−His/−Ade) in a series of dilutions of 10^–1^, 10^–2^, and 10^–3^. All experiments were independently performed 2 times with similar results.
(TIF)

S7 Fig DRN does not associate with the CRM locus downstream of *CLV3*. *UBQ10::mCherry-WUS-GR/UBQ10::DRN-GFP* lines (14-day-old seedlings) were used for ChIP assays. The nuclear localization of WUS-GR induced by DEX failed to confer the association of CRM with DRN-GFP, using the anti-GFP antibody for IP. The upstream -2,000 bp site acted as the negative control (no binding site). The experiments were independently performed 2 times with similar results. The data underlying this figure can be found in S1 Data.
(TIF)

S8 Fig The genetic interactions of *DRN*, *DRNL*, and *WUS* in SAMs. (A) The diagram showing the point mutation of *wus-7* mutants, and the T-DNA insertion of *wus-8* mutants. (B–I) The images of 10-day-old seedlings including WT, *drn-1, wus-7, drn-1 wus-7, wus-8, drnl-2, drn-1 drnl-2*, and *drn-1 drnl-2 wus-7*. The dotted circle indicates the meristem in H. Bars in B–G, 1 mm. Bars in H and I, 100 μm. (J–O) The in situ hybridization was performed in the wild-type and mutants (10-day-old seedlings) to check *CLV3* expression. Bars in J–L, O, and P, 30 μm. Bars in M and N, 100 μm. Bar in Q, 15 μm. All experiments were independently performed 2 times with similar results.
(TIF)

S9 Fig *DRN* and *WUS* collectively maintain SAM activity during reproductive stage. (A) The phenotypes of indicated mutants, including the shoots and SAMs, are shown. A portion of *drn-1 wus-7, wus-8*, and *drn-1 wus-8* mutants failed to produce shoots. Black scale bars in WT, *drn-1, wus-7*, and *drn-1 drnl-2*, 100 μm. Black scale bars in *drn-1 wus-7, wus-8*, and *drn-1 wus-8*, 1 mm. White scale bars, 5 mm. (B) The percentage of phenotypic plants in A was analyzed. (C) The SAM sizes of the plants in A were analyzed. Black bars, highest and lowest values; box, median 50%; black line in the box, median. ***P < 0.001; **P < 0.01; ns, no significant difference; Student’s *t* test. The experiments were independently performed 2 times with similar results. The data underlying this figure can be found in S1 Data.
(TIF)

S10 Fig The genetic interactions of *WUS, DRN, BRM*, and *CLV3* in SAMs. (A) The SAMs of indicated mutants are shown. The white arrows indicate the diameter of SAMs. Scale bars, 100 μm. (B) The SAM sizes of plants in A were analyzed. Black bars, highest and lowest values; box, median 50%; black line in the box, median. ***P < 0.001; ns, no significant difference; Student’s *t* test. The experiments were independently performed 2 times with similar results. The data underlying this figure can be found in S1 Data.
(TIF)

S11 Fig Identification of the region of BRM accounting for the interaction with DRN. (A) Diagram of the *BRM* coding sequence. (B) The full-length of DRN and truncated BRM were used for Y2H. BD and AD empty vectors were used as negative controls. Yeast cells were grown on the selective medium (SD/−Leu/−Trp/−His/−Ade) in a series of dilutions of 10^–1^, 10^–2^, and 10^–3^. The experiments were independently performed 2 times with similar results.
(TIF)

S12 Fig The negative controls in BiFC for detecting the interaction of DRN and BRM. ARF1 was used as the negative control in BiFC. YFP was split into the N-terminus and C-terminus, fused to ARF1, BRM, and DRN. Scale bars, 100 μm.
(TIF)

S13 Fig The interaction of DRN-BRM in SAMs. The *DRN::DRN-nYFP/BRM::BRM-cYFP* transgenic plants were used to detect DRN-BRM interactions in inflorescence SAMs. *DRN::nYFP* and *BRM::cYFP* were introduced as negative controls. Scale bars, 50 μm. The experiments were independently performed 2 times with similar results.
(TIF)

S14 Fig Identification of the region of DRN accounting for the interaction with BRM. (A) Diagram of the *DRN* coding sequence. (B) The full-length BRM, truncation and full-length DRN were used for Y2H. BD and AD empty vectors were used as negative controls. Yeast cells were grown on the selective medium (SD/−Leu/−Trp/−His/−Ade) in a series of dilutions of 10^–1^, 10^–2^, and 10^–3^. The experiments were independently performed 2 times with similar results.
(TIF)

S15 Fig Expression patterns of *DRN, WUS, CLV3*, and *BRM* in SAMs. (A) The distribution of DRN proteins in the SAM was checked using *DRN::DRN-GFP/drn-1* rescue lines during the reproductive stage, and 8 apices were analyzed. (B) The distribution of WUS proteins in the SAM was checked using *WUS::WUS-GFP/wus-8* rescue lines during the reproductive stage. Ten apices were analyzed. The red arrows indicate WUS-GFP signals in the L1 and L2 cell layers. (C) *CLV3* expression pattern was checked in *CLV3::GFP*/WT lines during the reproductive stage. Six apices were analyzed. (D) The top view of the SAM of *BRM::BRM-GFP* represents the L1 cell layer. Five apices were analyzed. Green, BRM-GFP signals; red, propidium iodide (PI) signals. (E) *BRM* mRNAs were detected by RNA in situ hybridization in the SAMs of wild-type plants. Seven apices were analyzed. (F) The *BRM* sense probe was used as a negative control. Five apices were analyzed. Scale bars in A–F, 50 μm. Bright field, BF, in A–C. All experiments were independently performed 2 times with similar results.
(TIF)

S16 Fig Expression of *BRM* and *CLV3* in ethanol inducible *amiBRM* plants. qRT-PCR was applied to check the relative transcript levels of *BRM* and *CLV3* in *35S::inducible amiBRM*/WT transgenic plants after 1% ethanol (EtOH) induction for 24 h using 14-day-old seedlings. Data represent means ± SDs from 3 biological replicates. *P < 0.1; **P < 0.01; Student’s *t* test. The experiments were independently performed 2 times with similar results. The data underlying this figure can be found in S1 Data.
(TIF)

S17 Fig The negative control for the FAIRE assays in Fig 4. The −2,000 site upstream of *CLV3* was selected to be the negative control of FAIRE assays in Fig 4C and 4D, using 10-day-old seedlings. Data represent means ± SDs from 3 biological replicates. ns, no significant difference; Student’s *t* test. The experiments were independently performed 2 times with similar results. The data underlying this figure can be found in S1 Data.
(TIF)

S18 Fig *DRN* fails to induce the nucleosome depletion of CRM region, downstream of *CLV3*. FAIRE assays were performed, using *35S::DRN-GR* lines (10-day-old seedlings) inducted with 15 μM DEX for 3 h, to check the chromatin state at CRM region, downstream of CLV3. The upstream −2,000 site was used as the negative control. Data represent means ± SDs from 3 biological replicates. ns, no significant difference; Student’s *t* test. The experiments were independently performed 2 times with similar results. The data underlying this figure can be found in S1 Data.
(TIF)

S19 Fig *CLV3* and *WUS* expression in WT, *drn-1, brm-3*, and *drn-1 brm-3*. *CLV3::GFP* and *WUS::GFP* lines were crossed with *drn-1, brm-3*, and *drn-1 brm-3*, respectively. *CLV3* and *WUS* expression was checked in these mutants by fluorescence. Six apices were analyzed in each line. Scale bars, 50 μm. The experiments were independently performed 2 times with similar results.
(TIF)

S20 Fig The genetic interactions of *DRN* and *BRM* in SAMs. (A) The phenotypes of indicated mutants, including the shoots and SAMs, are shown. The white arrows indicate the diameter of SAMs. Black scale bars, 100 μm. White scale bars, 5 mm. (B) The SAM sizes of plants in A were analyzed. Black bars, highest and lowest values; box, median 50%; black line in the box, median. ***P < 0.001; ns, no significant difference; Student’s *t* test. The experiments were independently performed 2 times with similar results. The data underlying this figure can be found in S1 Data.
(TIF)

S21 Fig Detecting the developmental timing in *drn-1, brm-3*, and *drn-1 brm-3*. The number of true leaves was analyzed in WT, *drn-1, brm-3, and drn-1 brm-3*, using 14-day-old seedlings. Black bars, highest and lowest values; box, median 50%. ns, no significant difference; Student’s *t* test. The experiments were independently performed 2 times with similar results. The data underlying this figure can be found in S1 Data.
(TIF)

S22 Fig Expression of *BRM* in *CLV3::inducible amiBRM* plants. *BRM* mRNAs were detected by RNA in situ hybridization in SAMs of *CLV3::inducible amiBRM* plants after 1% ethanol induction for 24 h during the reproductive stage. Scale bars, 50 μm. Ten apices were analyzed in each group. The experiments were independently performed 2 times with similar results.
(TIF)

S23 Fig The negative control for EtOH induction. (A and B) *CLV3::inducible GUS*/WT lines (14-day-old seedlings), as the negative control of Fig 6A and 6B, were used for EtOH induction. qRT-PCR was performed to test *CLV3* and *WUS* expression. Data represent means ± SDs from 3 biological replicates. (C and D) The SAM sizes of *CLV3::inducible GUS*/WT lines (14-day-old seedlings) with EtOH induction were analyzed, as the negative control of Fig 6C and 6D. The black arrows indicate the boundaries of SAMs. Scale bars, 50 μm. Black bars, highest and lowest values; box, median 50%; black line in the box, median. ns, no significant difference; Student’s *t* test. All experiments were independently performed 2 times with similar results. The data underlying this figure can be found in S1 Data.
(TIF)

S1 Data Values corresponding to figures. Values of histograms and box plots in main figures and supplemental figures.
(XLSX)

S1 Raw Images Raw gel images. Raw gels of co-IP, pull-down, and EMSA assays in this study. The gels used in figures and the repetitive results are included in this file.
(PDF)

## Abbreviations

BiFC: bimolecular fluorescence complementation
ChIP: chromatin immunoprecipitation
CRM: cis-regulatory module
CZ: central zone
DEX: dexamethasone
EMSA: electrophoretic mobility shift assay
FAIRE: formaldehyde-assisted isolation of regulatory elements
FM: floral meristem
HD: homeodomain
LB: lysogeny broth
MBP: maltose binding protein
NDR: nucleosome-depleted region
OC: organizing center
PI: propidium iodide
PZ: periphery zone
RM: rib meristem
SAM: shoot apical meristem
TF: transcription factor
Y2H: yeast two-hybrid
Y3H: yeast three-hybrid
