# Supplementary material for: DRN facilitates WUS transcriptional regulatory activity by chromatin remodeling to regulate shoot stem cell homeostasis in Arabidopsis
Source: PLoS Biol. 2024 Nov 8;22(11):e3002878. doi: 10.1371/journal.pbio.3002878 (PMC11548754; doi:10.1371/journal.pbio.3002878)
Supplement: S1 Table — (DOCX) [file pbio.3002878.s001.docx]

**S1 Table: Oligonucleotides used in this study**

| **Purpose** | **Gene** | **Sequence** |
| --- | --- | --- |
| qRT-PCR | *TUBULIN* | GAGCCTTACAACGCTACTCTGTCTGTC |
|  |  | ACACCAGACATAGTAGCAGAAATCAAG |
|  | *CLV3* | GATGAAAATGGAAAGTGAATGG |
|  |  | GGGAGCTGAAAGTTGTTTCTTG |
|  | *WUS* | ATCATGCAAGCTCAGGTACTGAATGT |
|  |  | GAGCTTTAATCCCGAGCGACACCGG |
|  | *BRM* | CCCACTCATCCAAACAACAG |
|  |  | GCTAGGCCGTCTTTTACCA |
| Constructions for Y2H | BD-WUS | CCCGCATATGATGGAGCCGCCACAGCAT |
|  |  | GGATCCCTAGTTCAGACGTAGCTCAAG |
|  | AD-WUS | CCCGCATATGATGGAGCCGCCACAGCAT |
|  |  | GGATCCCTAGTTCAGACGTAGCTCAAG |
|  | BD-WUS_1-99Aa_ | CCGGAATTCATGGAGCCGCCACAGCATC |
|  |  | CGCGGATCCCTACTTCTTCTGACGCTCACG |
|  | BD-WUS_100-249Aa_ | CCGGAATTCAGATTCAACGGAACAAACA |
|  |  | CGCGGATCCCTACAGATAAGCATCGCCACC |
|  | BD-WUS_250-292Aa_ | CCGGAATTCGAACATCGACGTACGCTTCC |
|  |  | CGCGGATCCCTAGTTCAGACGTAGCTC |
|  | AD-DRN | CCGGAATTCATGGAAAAAGCCTTGAGAAAC |
|  |  | CGCGGATCCCTATCCCCACGATCTTCGGC |
|  | AD-DRN_1-55Aa_ | CCGGAATTCATGGAAAAAGCCTTGAGAAAC |
|  |  | CGCGGATCCCTACGTCGTGCTGCTGCCAGC |
|  | AD-DRN_56-113Aa_ | CCGGAATTCAGGTACCGCGGCGTACGCCGG |
|  |  | CGCGGATCCCTAAGTAAAATTAGTACGAGCC |
|  | AD-DRN_1-113Aa_ | CCGGAATTCATGGAAAAAGCCTTGAGAAAC |
|  |  | CGCGGATCCCTAAGTAAAATTAGTACGAGCC |
|  | AD-DRN_114-328Aa_ | CCGGAATTCTATCCGACAGCTGTCATTATGC |
|  |  | CCCGGATCCCTATCCCCACGATCTTCGGCAAG |
|  | BD-BRM | ATGGCCATGGAGGCCGAATTCATGCAATCTGGAGGCAGTGG |
|  |  | TCGACGGATCCCCGGGAATTCCTATAAATGGCTAGGCCGTCTTTT |
|  | BD-BRM_1-976Aa_ | TGGCCATGGAGGCCGAATTCATGCAATCTGGAGGC |
|  |  | CGGATCCCCGGGAATTCCTAAAGCATTGAAGGTTG |
|  | BD-BRM_977-2193Aa_ | TGGCCATGGAGGCCGAATTCCAGGCTGGAACTTTG |
|  |  | CGACGGATCCCCGGGAATTCCTATAAATGGCTAGG |
|  | AD-ARR7 | CCATGGAGGCCAGTGAATTCATGGCGGTTGGTGAG |
|  |  | TGCCCACCCGGGTGGAATTCCTAAAGTAGAGAAAA |
|  | AD-TPL | CCATGGAGGCCAGTGAATTCATGTCTTCTCTTAGT |
|  |  | TGCCCACCCGGGTGGAATTCCTATCTCTGAGGCTG |
| Constructions for Y3H | pBridge (*BD-WUS pMET25::DRN*) | CCCCATATGATGGAGCCGCCACAGCAT |
|  |  | CCCGGATCCCTAGTTCAGACGTAGCTCAAGAG |
|  |  | CCCGCGGCCGCAATGGAAAAAGCCTTGAGAAACT |
|  |  | CCCAAGATCTCTATCCCCACGATCTTCGGC |
| Constructions for protein expression | GST-WUS | CCCGGATCCATGGAGCCGCCACAGCATC |
|  |  | CCCCTCGAGCTAGTTCAGACGTAGCTCAAG |
|  | 8His-MBP-WUS | CCCGTCGACATGGAGCCGCCACAGCATC |
|  |  | CCCGCGGCCGCCTAGTTCAGACGTAGCTCAAG |
|  | 8His-MBP-DRN | CCCGTCGACATGGAAAAAGCCTTGAG |
|  |  | CCCCTCGAGCTAGCATGCCGTTTTCGATGCTG |
|  | 8His-MBP-BRM | CCCGTCGACATGCAATCTGGAGGCAGTG |
|  |  | CCCGCGGCCGCCTAAAGCATTGAAGGTTGTC |
| Constructions for co-IP | WUS-3×HA | CCCACTAGTATGGAGCCGCCACAGCATCAGC |
|  |  | CCCACTAGTGTTCAGACGTAGCTCAAGAG |
|  | DRN-Flag | CCCGTCGACATGGAAAAAGCCTTGAGAAAC |
|  |  | CCCGTCGACTCCCCACGATCTTCGGCAAG |
|  | BRM-3×HA | CCCACTAGTATGCAATCTGGAGGCAGTGG |
|  |  | CCCACTAGTAAGCATTGAAGGTTGTCTTAC |
| Constructions for BiFC | PJLBlue-WUS | CCCAAGCTTATGGAGCCGCCACAGCATCAG |
|  |  | CCCGCGGCCGCAGTTCAGACGTAGCTCAAGAGAAG |
|  | PJLBlue-DRN | CCCCTCGAGATGGAAAAAGCCTTGAGAA |
|  |  | CCCGCGGCCGCCTCCCCACGATCTTCGGCAAG |
|  | PJLBlue-BRM | CCCGTCGACATGCAATCTGGAGGCAGTGG |
|  |  | CCCGCGGCCGCCAAGCATTGAAGGTTGTCTTAC |
| Probes for EMSA | *CLV3* | GGCTCATATAATCCATTCAATTTATG |
|  |  | CATAAATTGAATGGATTATATGAGCC |
| Probes for *in situ* hybridization | *CLV3* | AGTTTCTATATTTCTCTCTT |
|  |  | TTTTCTTAGAAAATCATGAGAT |
|  | *BRM* | ATGCAATCTGGAGGCAGTGGCGGA |
|  |  | AAGCATTGAAGGTTGTCTTACAA |
|  | *STM* | ATGGAGAGTGGTTCCAACAGC |
|  |  | ACACACCACAACATCAAACGC |
| FAIRE | *CLV3* | GAAACCTGATCGATATGGCAG |
|  |  | AAGGAGGTAAGATTCTGCAGC |
|  |  | CTCTCTGCCTCATGACATCAG |
|  |  | AATATGGATGATACCTTAATCGG |
|  |  | Tccaaagcaatgtaccgttggg |
|  |  | acactgacactgcctgtcactg |
|  | *Ta3* | TGCATGCGTCTATGTCAAGC |
|  |  | ATCTCAGCCTTGCTAGCTCC |
| ChIP | *CLV3* | GAAACCTGATCGATATGGCAG |
|  |  | AAGGAGGTAAGATTCTGCAGC |
|  |  | CTCTCTGCCTCATGACATCAG |
|  |  | AATATGGATGATACCTTAATCGG |
|  |  | ACTCAAGCTCATGCTCACGTTC |
|  |  | AACTGGACCGGACATCTACATG |
|  |  | Tccaaagcaatgtaccgttggg |
|  |  | acactgacactgcctgtcactg |
