# Supplementary figures and images for: DRN facilitates WUS transcriptional regulatory activity by chromatin remodeling to regulate shoot stem cell homeostasis in Arabidopsis
Source: PLoS Biol. 2024 Nov 8;22(11):e3002878. doi: 10.1371/journal.pbio.3002878 (PMC11548754; doi:10.1371/journal.pbio.3002878)

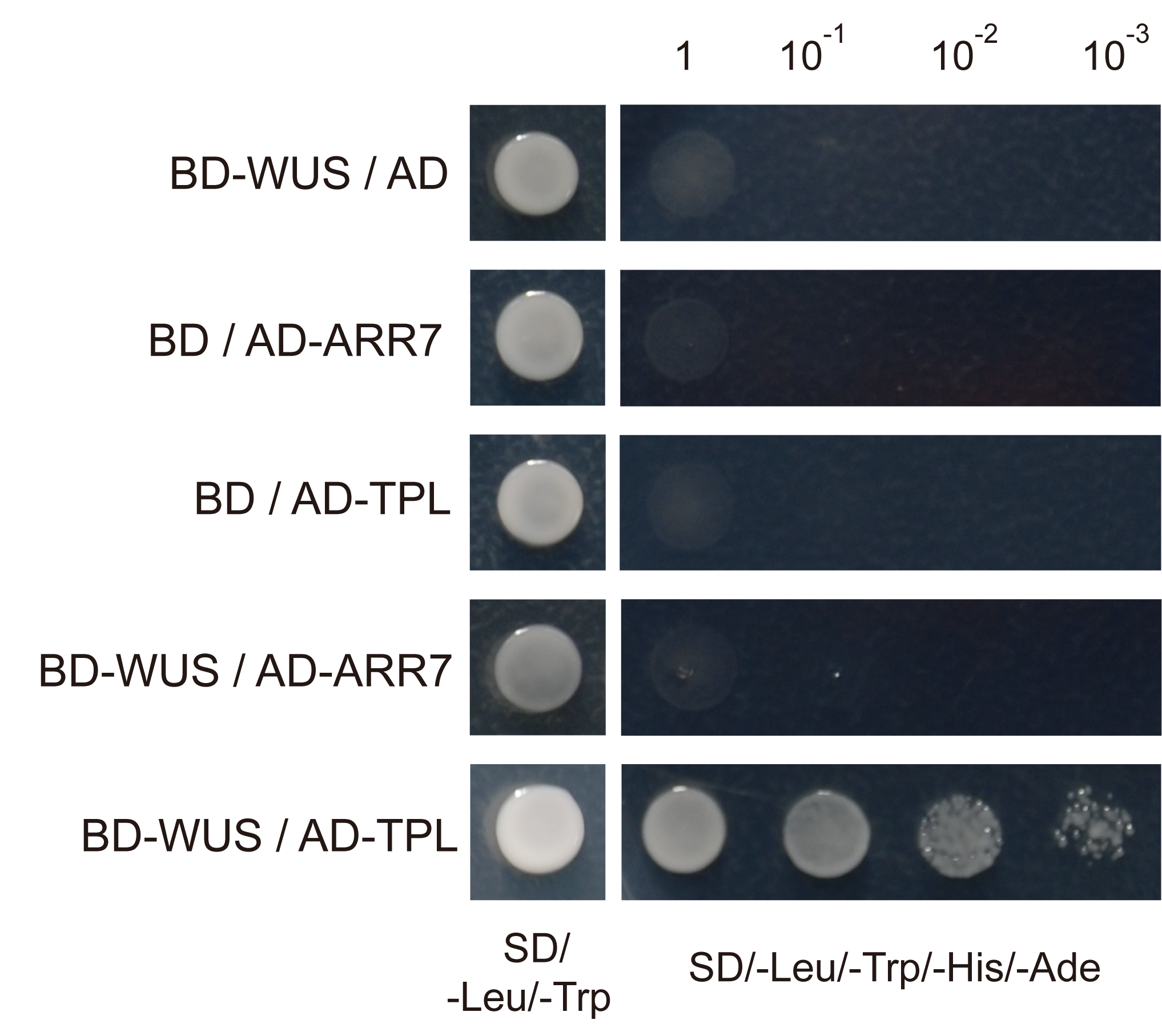

Supplement: S1 Fig — Yeast cells were grown on the selective medium (SD/−Leu/−Trp/−His/−Ade) in a series of dilutions of 10–1, 10–2, and 10–3. ARR7 and TPL served as the negative and positive controls, respectively. Two independent experiments were performed with similar results. (TIF) [file pbio.3002878.s002.tif]

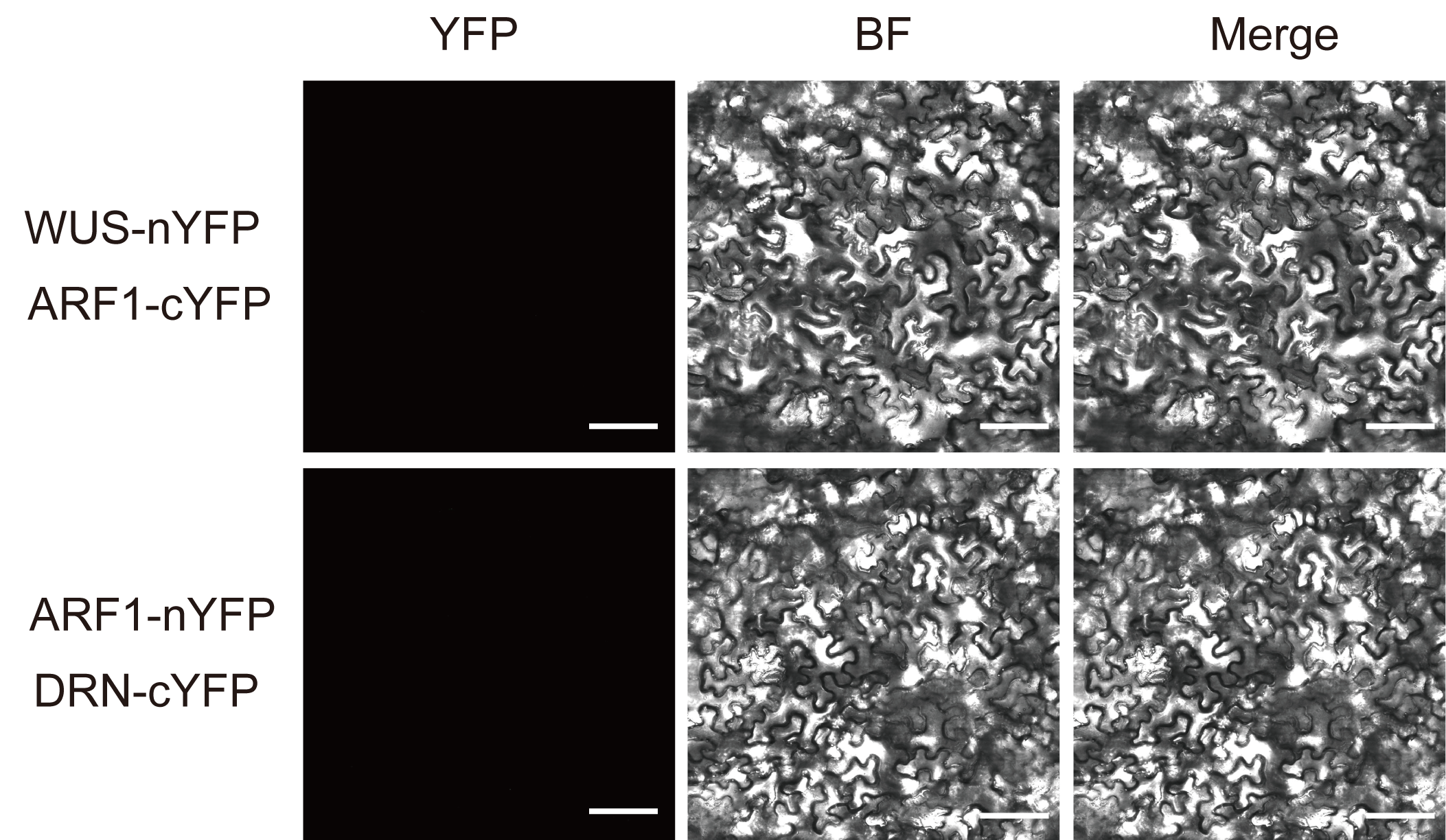

Supplement: S2 Fig — ARF1 was used as the negative controls in BiFC. YFP was split into the N-terminus and C-terminus, fused to WUS, ARF1, and DRN. Scale bars, 100 μm. (TIF) [file pbio.3002878.s003.tif]

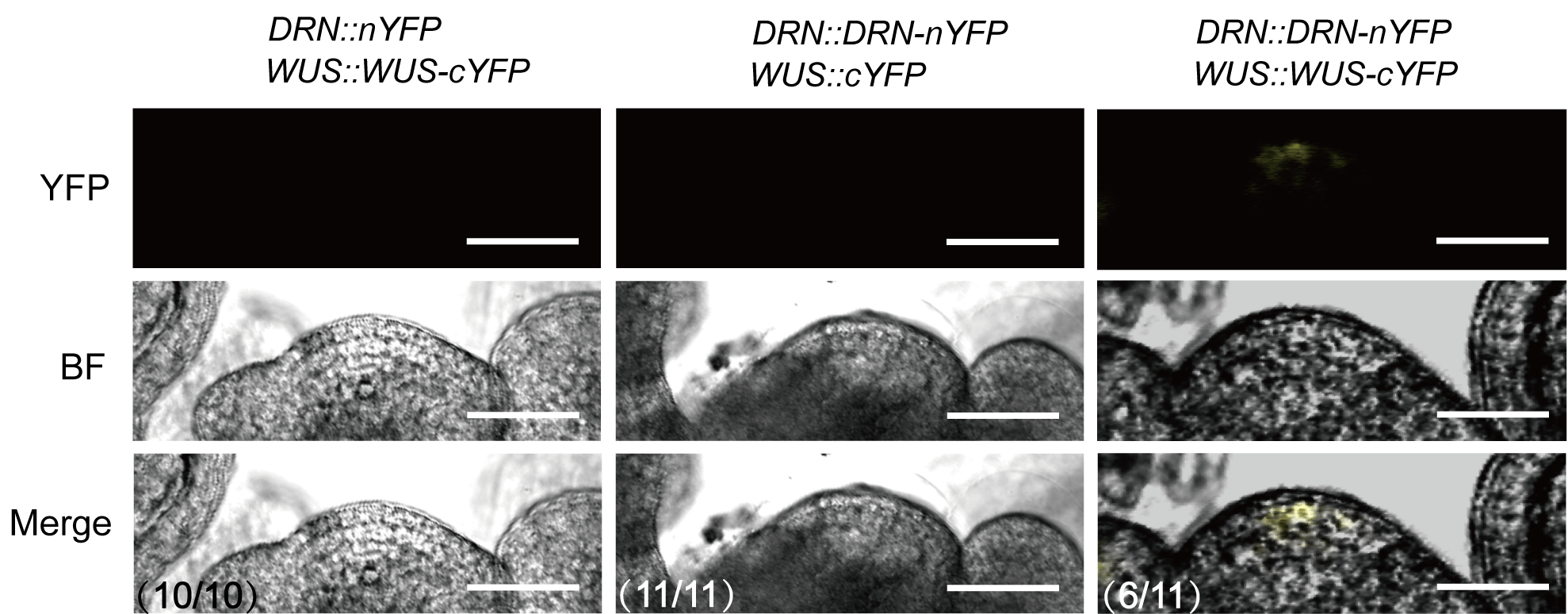

Supplement: S3 Fig — The DRN::DRN-nYFP/WUS::WUS-cYFP transgenic plants were used to detect DRN-WUS interactions in inflorescence SAMs. DRN::nYFP and WUS::cYFP were introduced as negative controls. Scale bars, 50 μm. Two independent experiments were performed with similar results. (TIF) [file pbio.3002878.s004.tif]

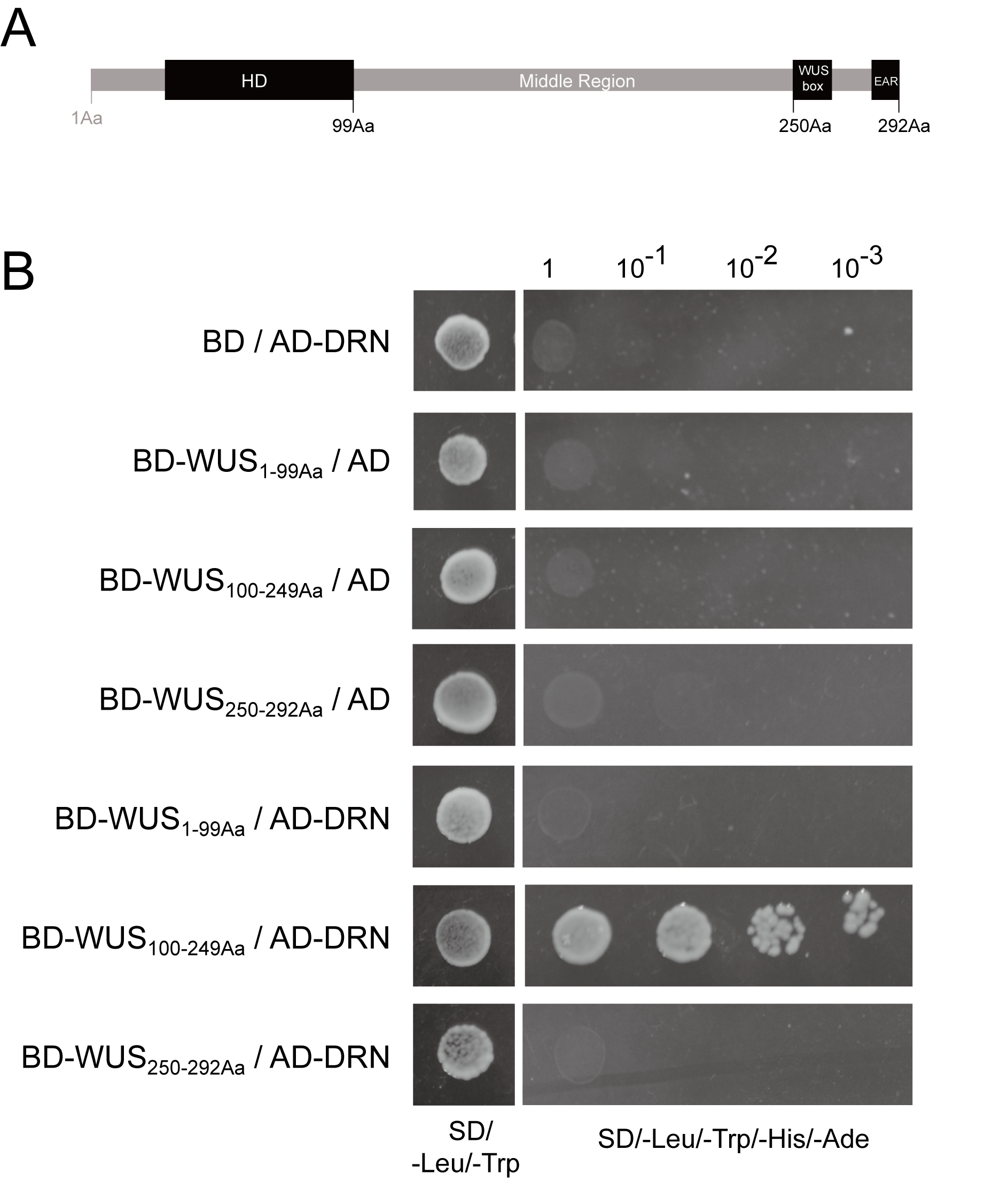

Supplement: S4 Fig — (A) Diagram of the WUS coding sequence. (B) Truncated WUS and full-length DRN were used for Y2H. BD and AD empty vectors were introduced as negative controls. Yeast cells were grown on the selective medium (SD/−Leu/−Trp/−His/−Ade) in a series of dilutions of 10–1, 10–2, and 10–3. The experiments were independently performed two times with similar results. (TIF) [file pbio.3002878.s005.tif]

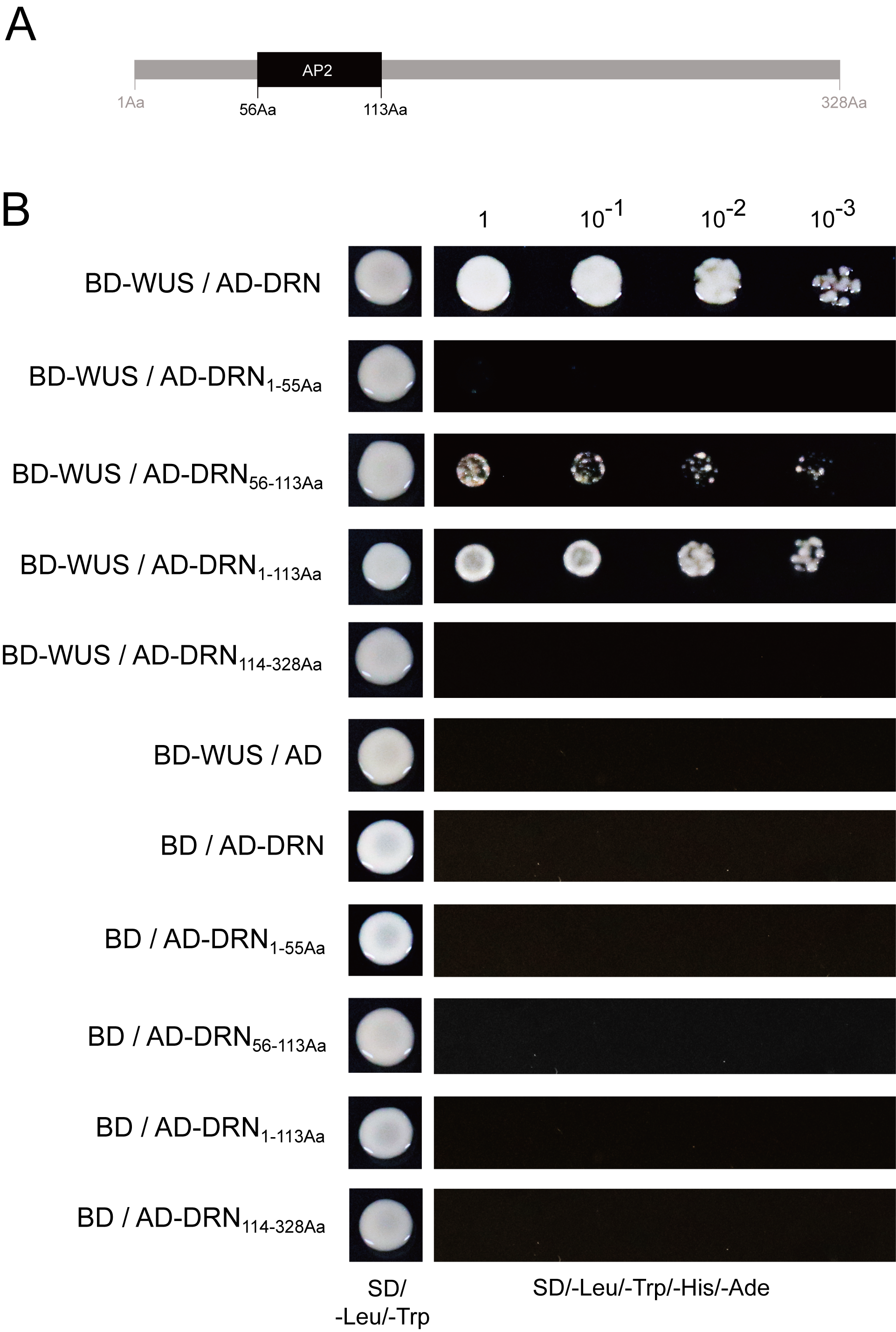

Supplement: S5 Fig — (A) Diagram of DRN coding sequence. (B) The full-length WUS, truncated and full-length DRN were used for Y2H. BD and AD empty vectors were used as negative controls. Yeast cells were grown on the selective medium (SD/−Leu/−Trp/−His/−Ade) in a series of dilutions of 10–1, 10–2, and 10–3. The experiments were independently performed 2 times with similar results. (TIF) [file pbio.3002878.s006.tif]

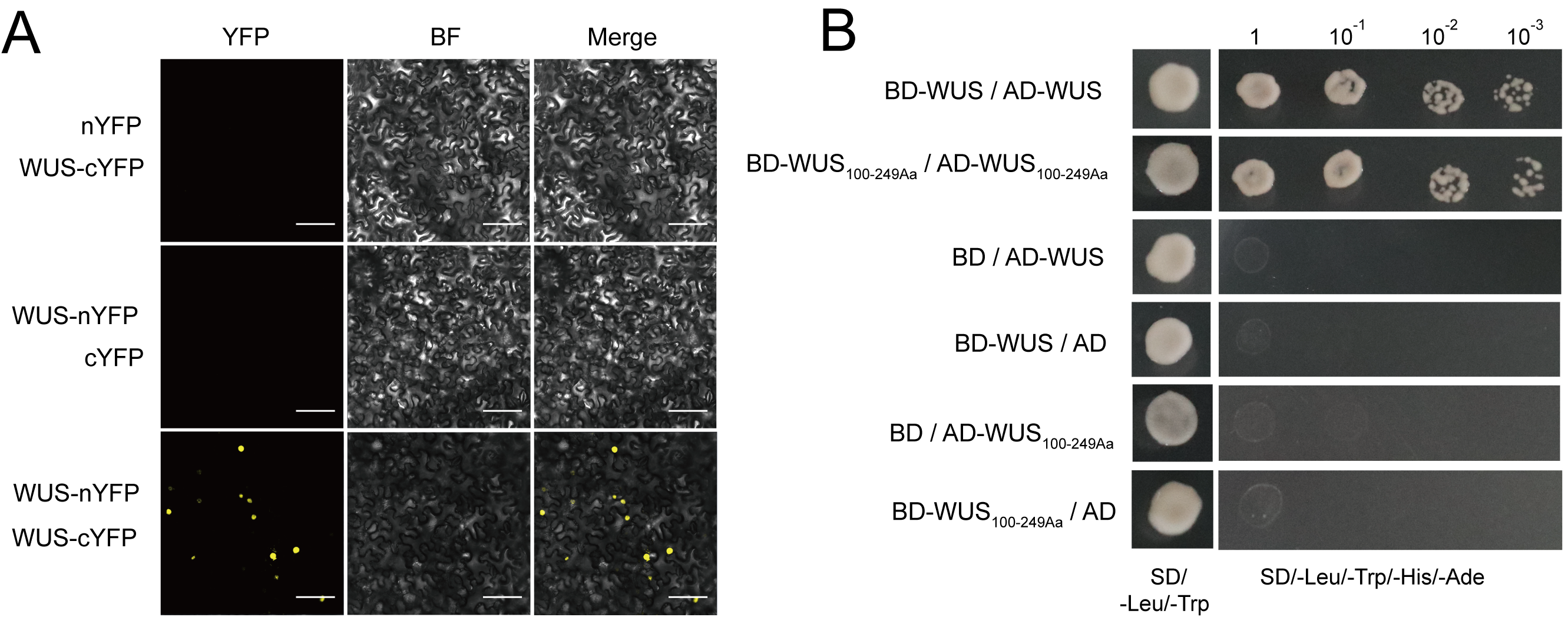

Supplement: S6 Fig — (A) BiFC exhibiting that the interaction of WUS-WUS in tobacco leaves. YFP was split into the N-terminus and C-terminus, fused to WUS, respectively. Scale bars, 100 μm. (B) The full-length and truncated WUS were used for Y2H. Yeast cells were grown on the selective medium (SD/−Leu/−Trp/−His/−Ade) in a series of dilutions of 10–1, 10–2, and 10–3. All experiments were independently performed 2 times with similar results. (TIF) [file pbio.3002878.s007.tif]

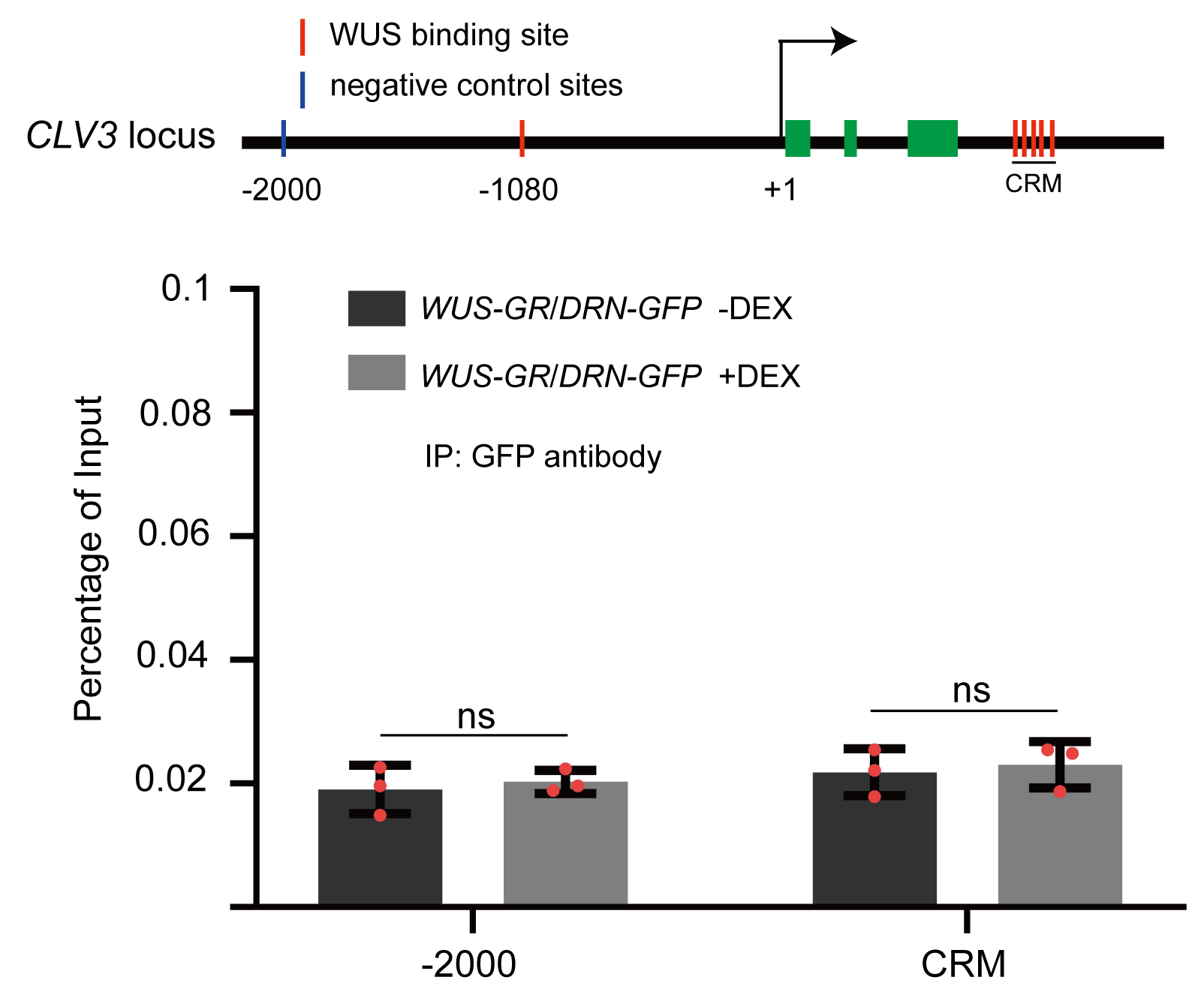

Supplement: S7 Fig — UBQ10::mCherry-WUS-GR/UBQ10::DRN-GFP lines (14-day-old seedlings) were used for ChIP assays. The nuclear localization of WUS-GR induced by DEX failed to confer the association of CRM with DRN-GFP, using the anti-GFP antibody for IP. The upstream -2,000 bp site acted as the negative control (no binding site). The experiments were independently performed 2 times with similar results. The data underlying this figure can be found in S1 Data. (TIF) [file pbio.3002878.s008.tif]

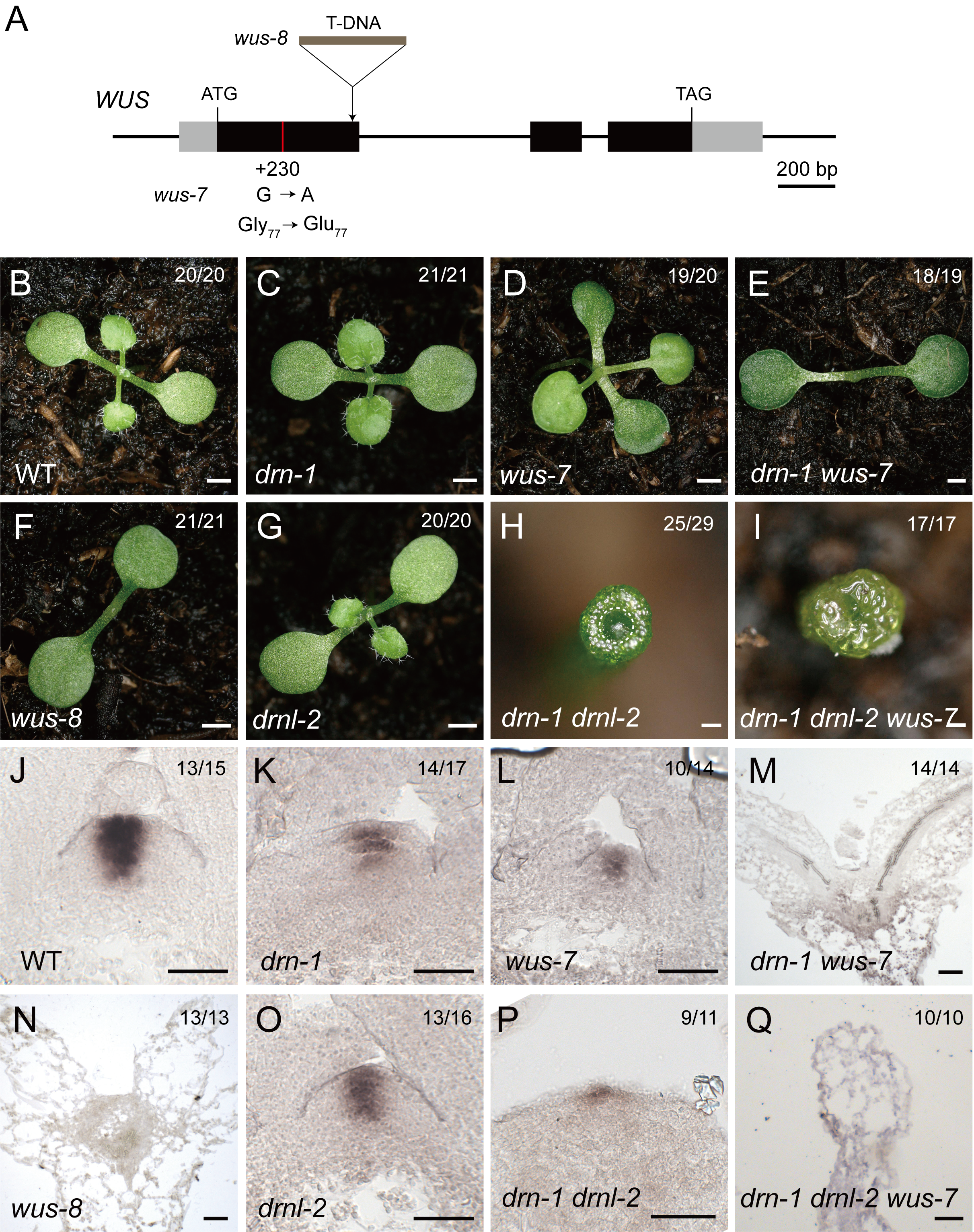

Supplement: S8 Fig — (A) The diagram showing the point mutation of wus-7 mutants, and the T-DNA insertion of wus-8 mutants. (B–I) The images of 10-day-old seedlings including WT, drn-1, wus-7, drn-1 wus-7, wus-8, drnl-2, drn-1 drnl-2, and drn-1 drnl-2 wus-7. The dotted circle indicates the meristem in H. Bars in B–G, 1 mm. Bars in H and I, 100 μm. (J–O) The in situ hybridization was performed in the wild-type and mutants (10-day-old seedlings) to check CLV3 expression. Bars in J–L, O, and P, 30 μm. Bars in M and N, 100 μm. Bar in Q, 15 μm. All experiments were independently performed 2 times with similar results. (TIF) [file pbio.3002878.s009.tif]

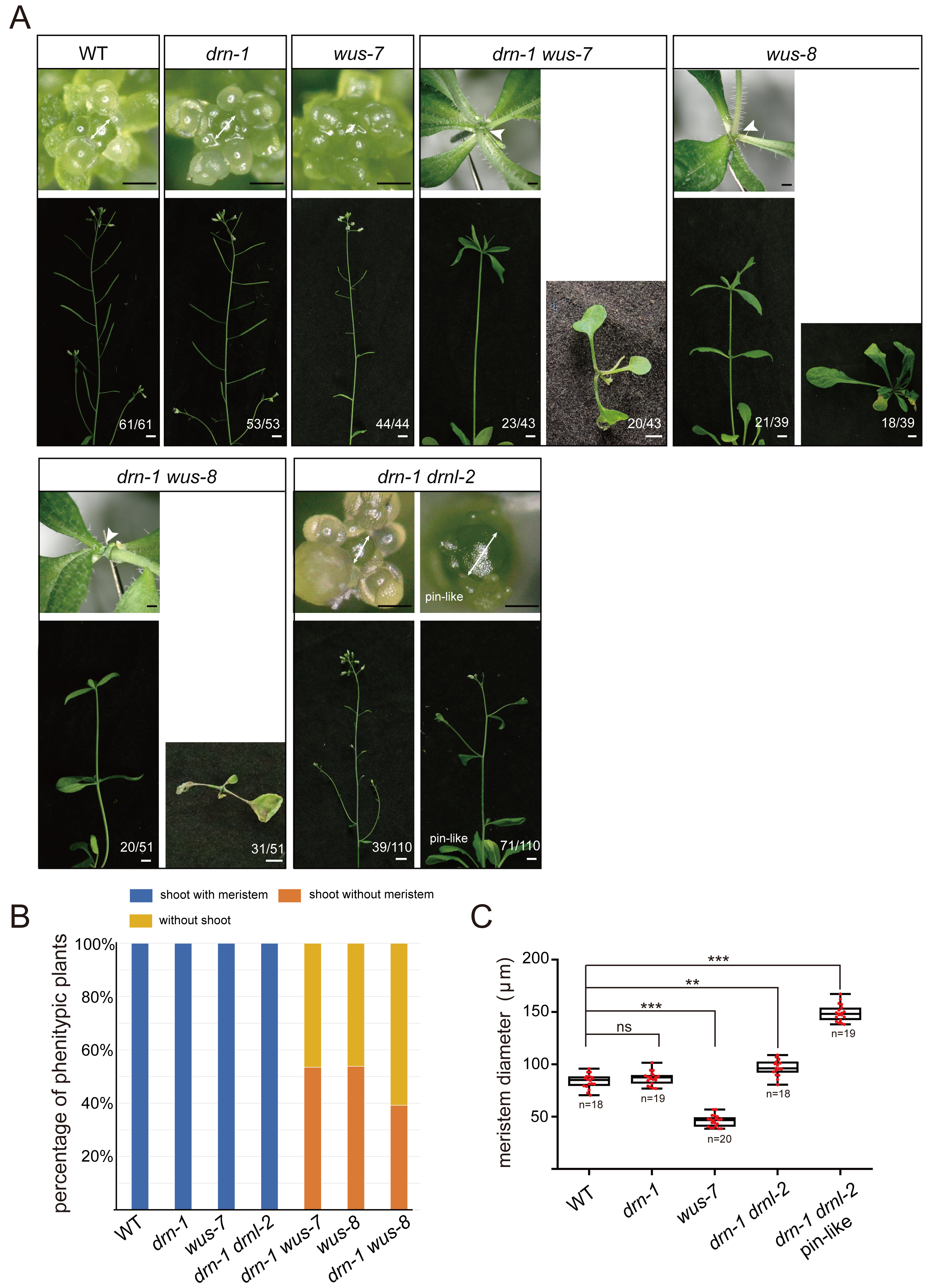

Supplement: S9 Fig — (A) The phenotypes of indicated mutants, including the shoots and SAMs, are shown. A portion of drn-1 wus-7, wus-8, and drn-1 wus-8 mutants failed to produce shoots. Black scale bars in WT, drn-1, wus-7, and drn-1 drnl-2, 100 μm. Black scale bars in drn-1 wus-7, wus-8, and drn-1 wus-8, 1 mm. White scale bars, 5 mm. (B) The percentage of phenotypic plants in A was analyzed. (C) The SAM sizes of the plants in A were analyzed. Black bars, highest and lowest values; box, median 50%; black line in the box, median. ***P < 0.001; **P < 0.01; ns, no significant difference; Student’s t test. The experiments were independently performed 2 times with similar results. The data underlying this figure can be found in S1 Data. (TIF) [file pbio.3002878.s010.tif]

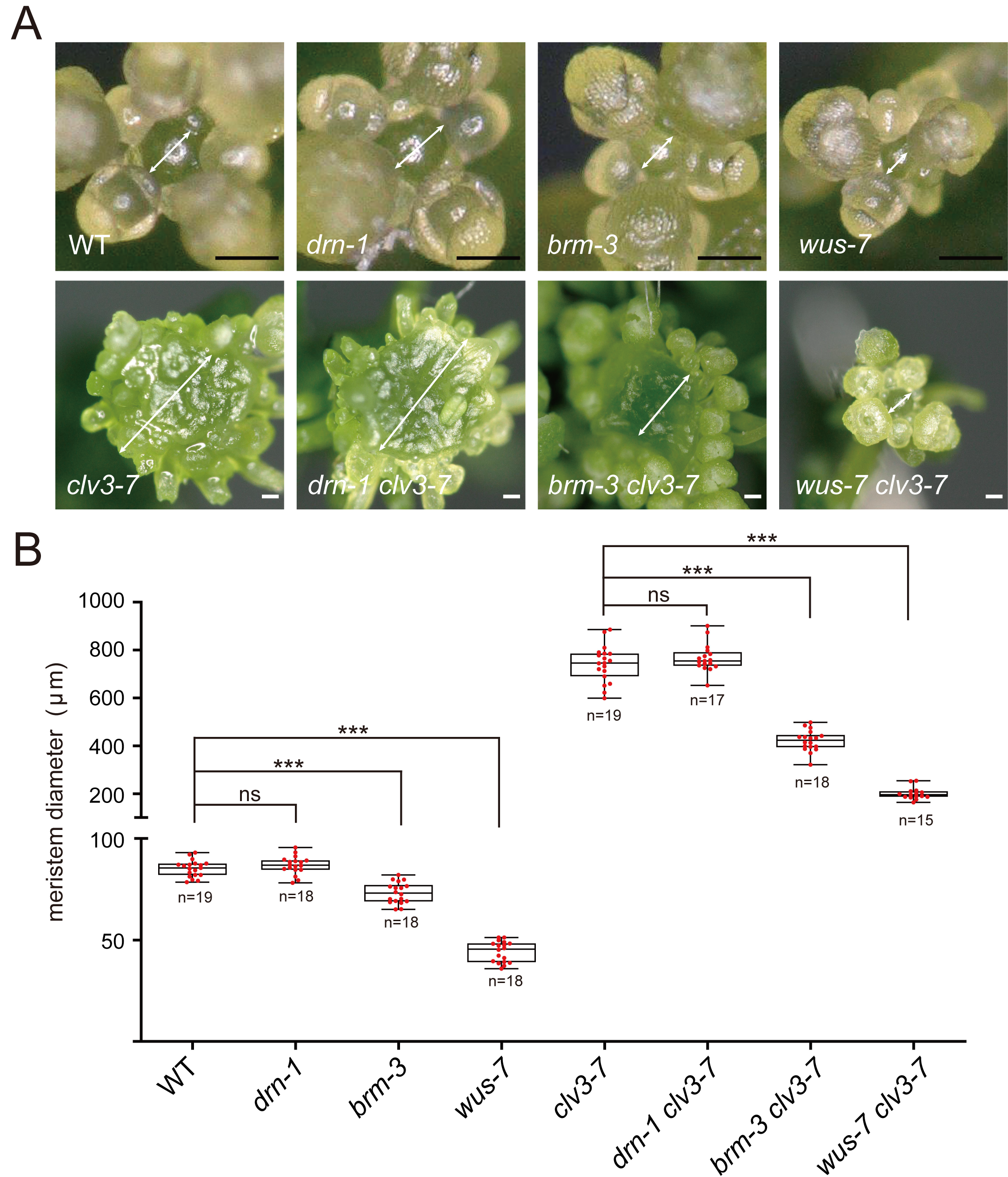

Supplement: S10 Fig — (A) The SAMs of indicated mutants are shown. The white arrows indicate the diameter of SAMs. Scale bars, 100 μm. (B) The SAM sizes of plants in A were analyzed. Black bars, highest and lowest values; box, median 50%; black line in the box, median. ***P < 0.001; ns, no significant difference; Student’s t test. The experiments were independently performed 2 times with similar results. The data underlying this figure can be found in S1 Data. (TIF) [file pbio.3002878.s011.tif]

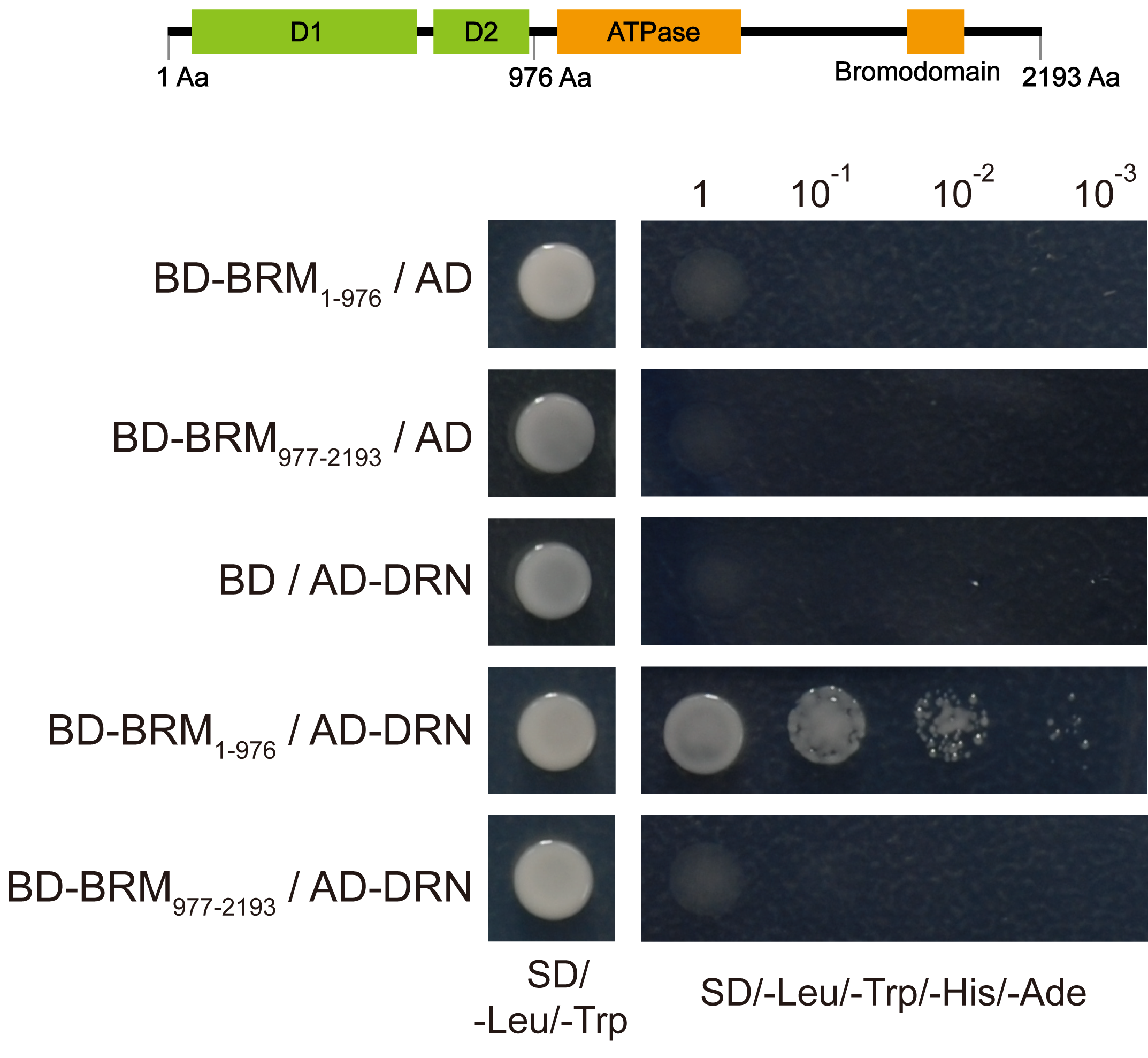

Supplement: S11 Fig — (A) Diagram of the BRM coding sequence. (B) The full-length of DRN and truncated BRM were used for Y2H. BD and AD empty vectors were used as negative controls. Yeast cells were grown on the selective medium (SD/−Leu/−Trp/−His/−Ade) in a series of dilutions of 10–1, 10–2, and 10–3. The experiments were independently performed 2 times with similar results. (TIF) [file pbio.3002878.s012.tif]

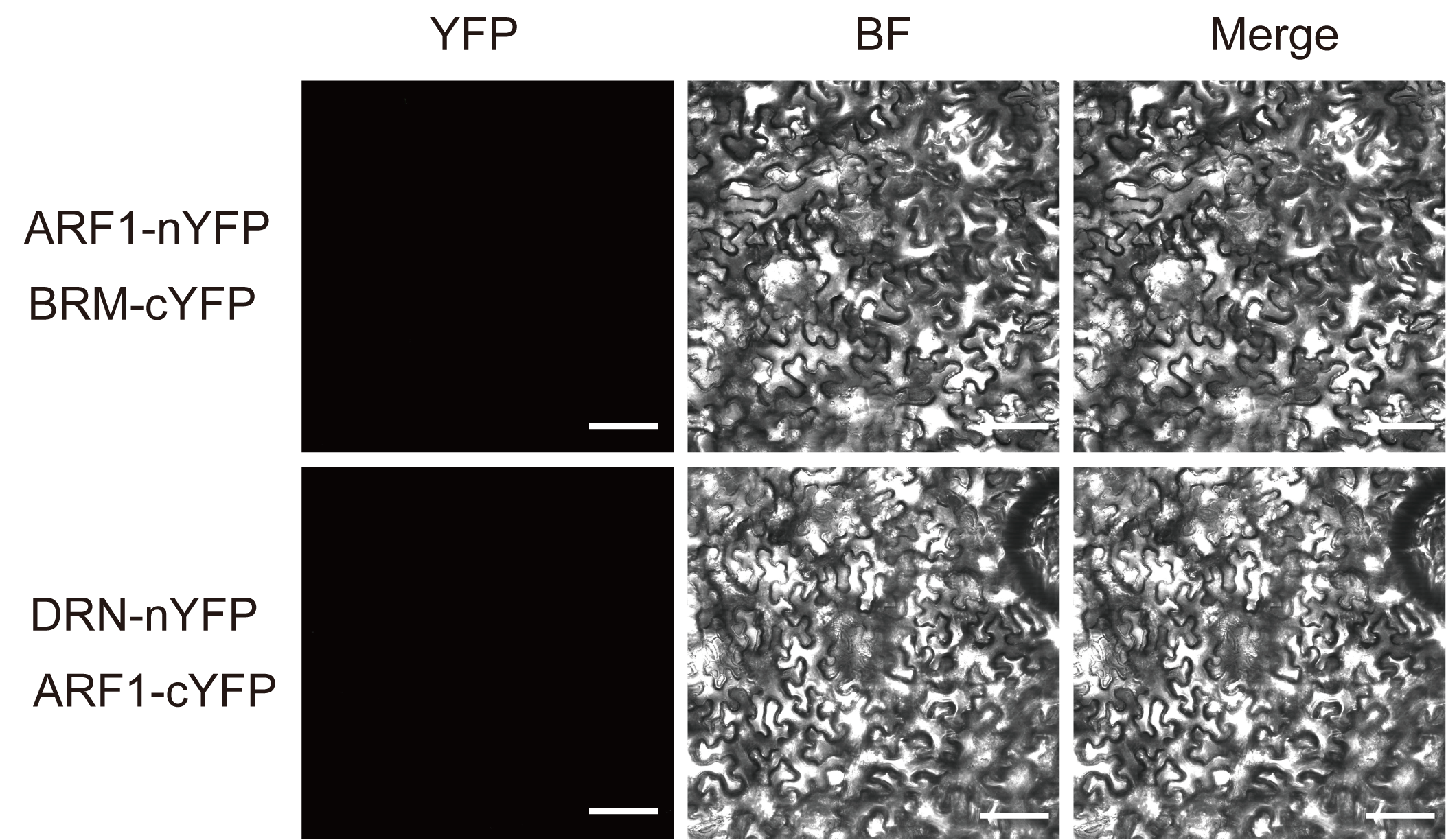

Supplement: S12 Fig — ARF1 was used as the negative control in BiFC. YFP was split into the N-terminus and C-terminus, fused to ARF1, BRM, and DRN. Scale bars, 100 μm. (TIF) [file pbio.3002878.s013.tif]

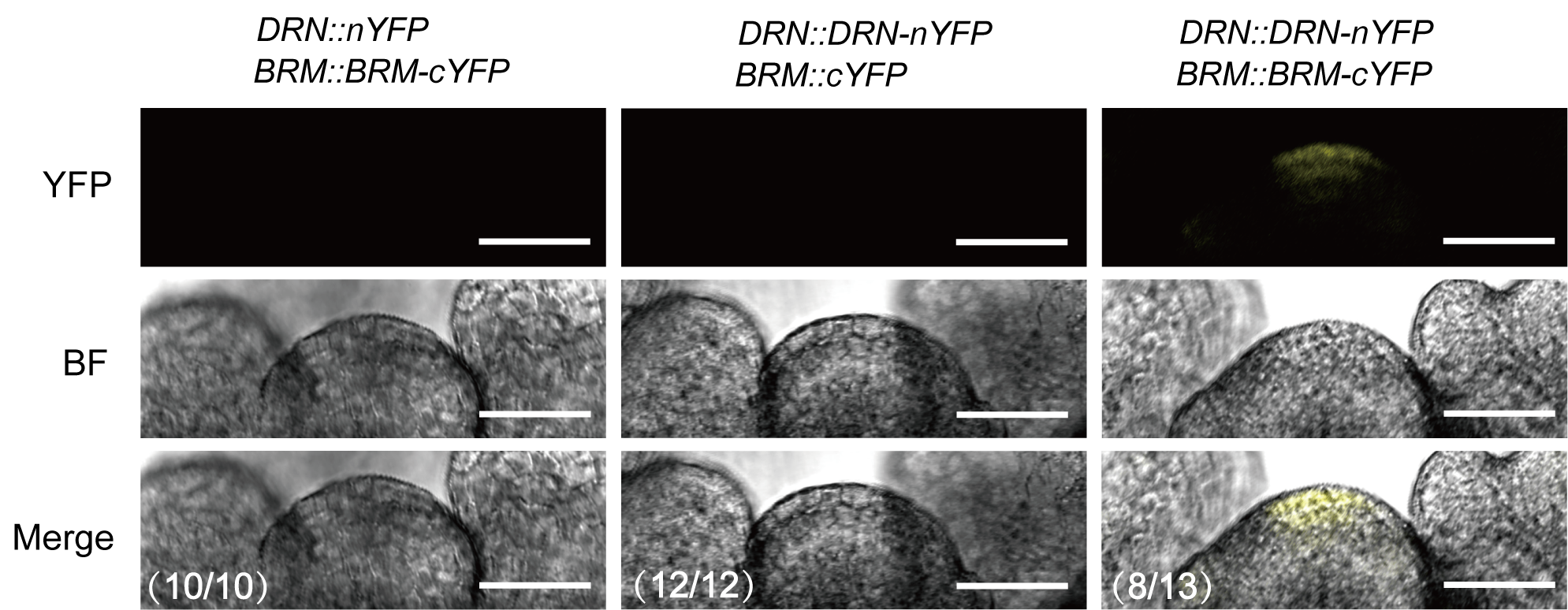

Supplement: S13 Fig — The DRN::DRN-nYFP/BRM::BRM-cYFP transgenic plants were used to detect DRN-BRM interactions in inflorescence SAMs. DRN::nYFP and BRM::cYFP were introduced as negative controls. Scale bars, 50 μm. The experiments were independently performed 2 times with similar results. (TIF) [file pbio.3002878.s014.tif]

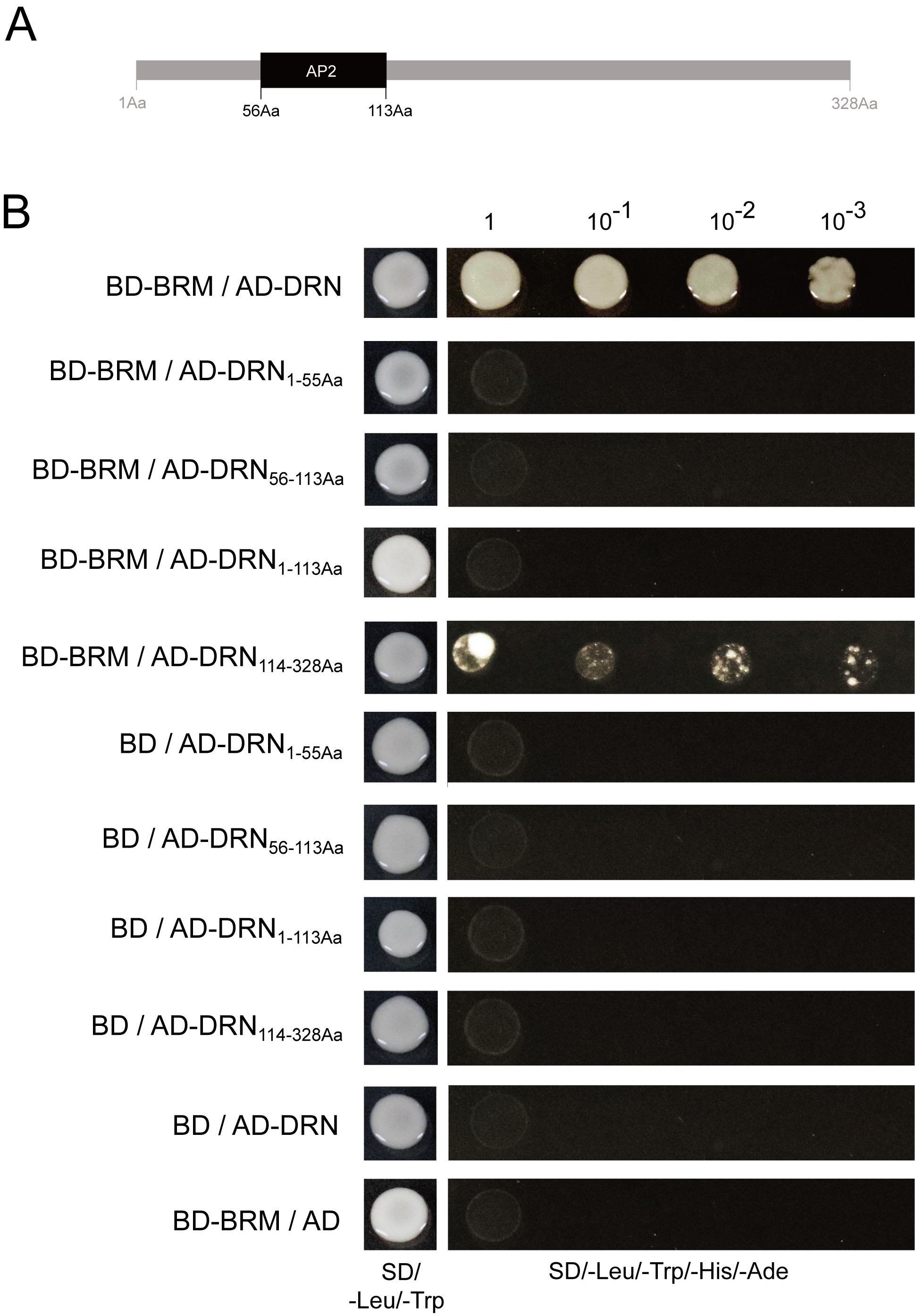

Supplement: S14 Fig — (A) Diagram of the DRN coding sequence. (B) The full-length BRM, truncation and full-length DRN were used for Y2H. BD and AD empty vectors were used as negative controls. Yeast cells were grown on the selective medium (SD/−Leu/−Trp/−His/−Ade) in a series of dilutions of 10–1, 10–2, and 10–3. The experiments were independently performed 2 times with similar results. (TIF) [file pbio.3002878.s015.tif]

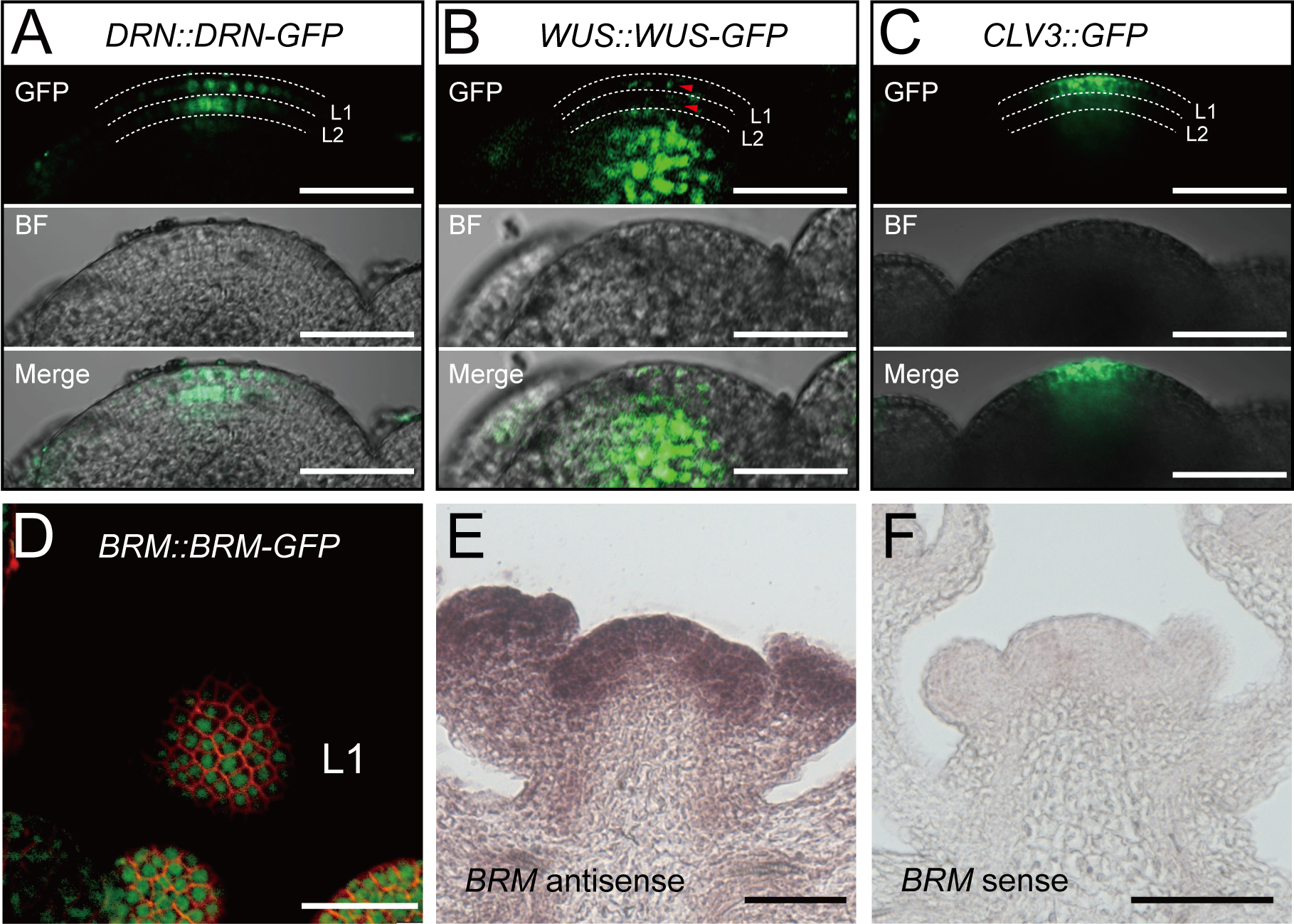

Supplement: S15 Fig — (A) The distribution of DRN proteins in the SAM was checked using DRN::DRN-GFP/drn-1 rescue lines during the reproductive stage, and 8 apices were analyzed. (B) The distribution of WUS proteins in the SAM was checked using WUS::WUS-GFP/wus-8 rescue lines during the reproductive stage. Ten apices were analyzed. The red arrows indicate WUS-GFP signals in the L1 and L2 cell layers. (C) CLV3 expression pattern was checked in CLV3::GFP/WT lines during the reproductive stage. Six apices were analyzed. (D) The top view of the SAM of BRM::BRM-GFP represents the L1 cell layer. Five apices were analyzed. Green, BRM-GFP signals; red, propidium iodide (PI) signals. (E) BRM mRNAs were detected by RNA in situ hybridization in the SAMs of wild-type plants. Seven apices were analyzed. (F) The BRM sense probe was used as a negative control. Five apices were analyzed. Scale bars in A–F, 50 μm. Bright field, BF, in A–C. All experiments were independently performed 2 times with similar results. (TIF) [file pbio.3002878.s016.tif]

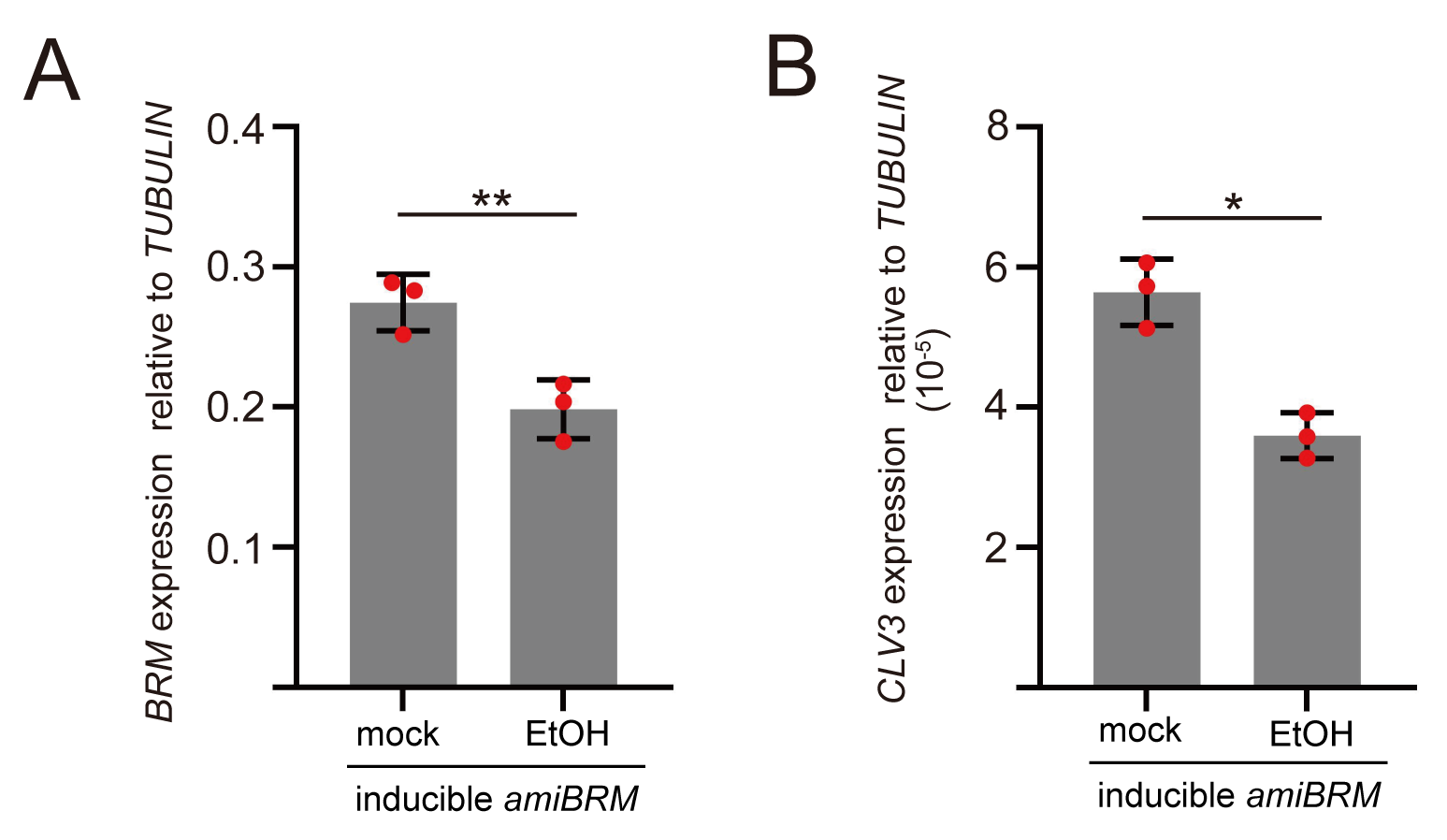

Supplement: S16 Fig — qRT-PCR was applied to check the relative transcript levels of BRM and CLV3 in 35S::inducible amiBRM/WT transgenic plants after 1% ethanol (EtOH) induction for 24 h using 14-day-old seedlings. Data represent means ± SDs from 3 biological replicates. *P < 0.1; **P < 0.01; Student’s t test. The experiments were independently performed 2 times with similar results. The data underlying this figure can be found in S1 Data. (TIF) [file pbio.3002878.s017.tif]

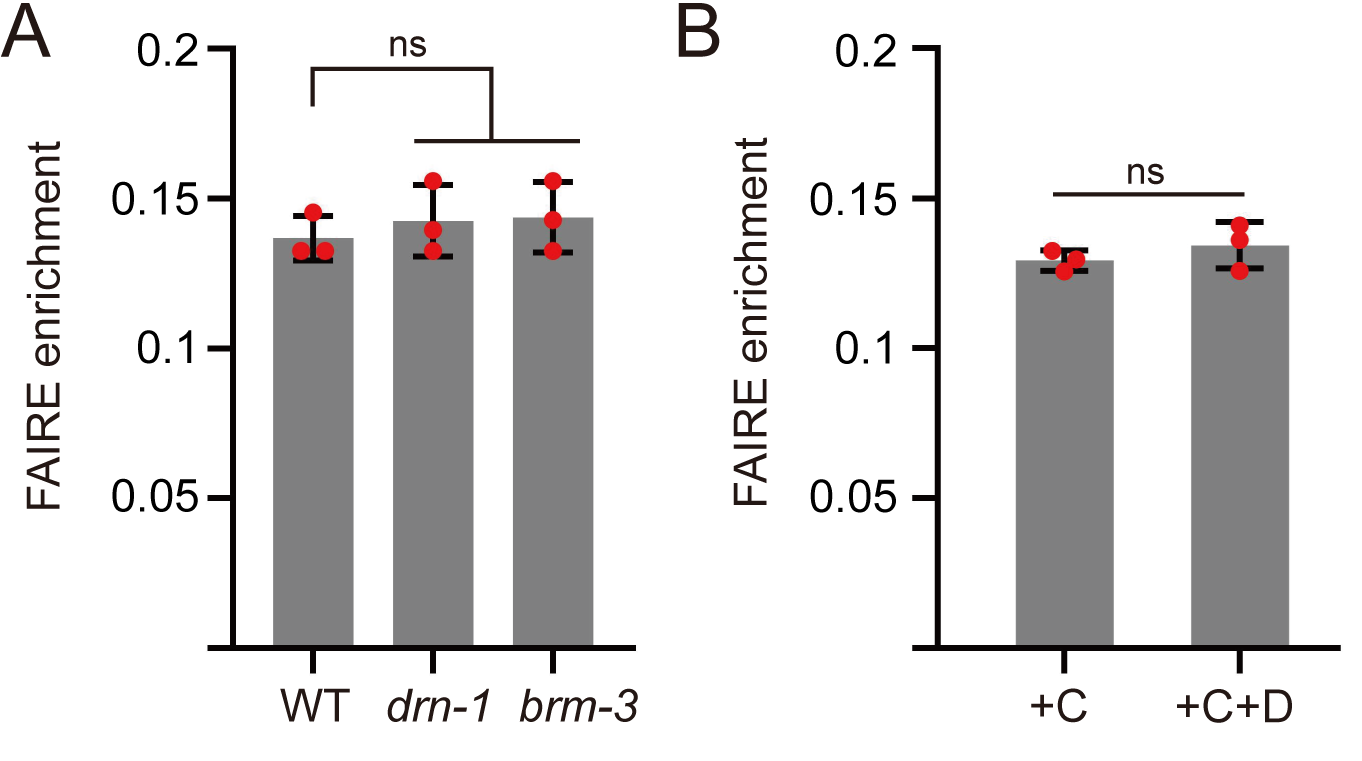

Supplement: S17 Fig — The −2,000 site upstream of CLV3 was selected to be the negative control of FAIRE assays in Fig 4C and 4D, using 10-day-old seedlings. Data represent means ± SDs from 3 biological replicates. ns, no significant difference; Student’s t test. The experiments were independently performed 2 times with similar results. The data underlying this figure can be found in S1 Data. (TIF) [file pbio.3002878.s018.tif]

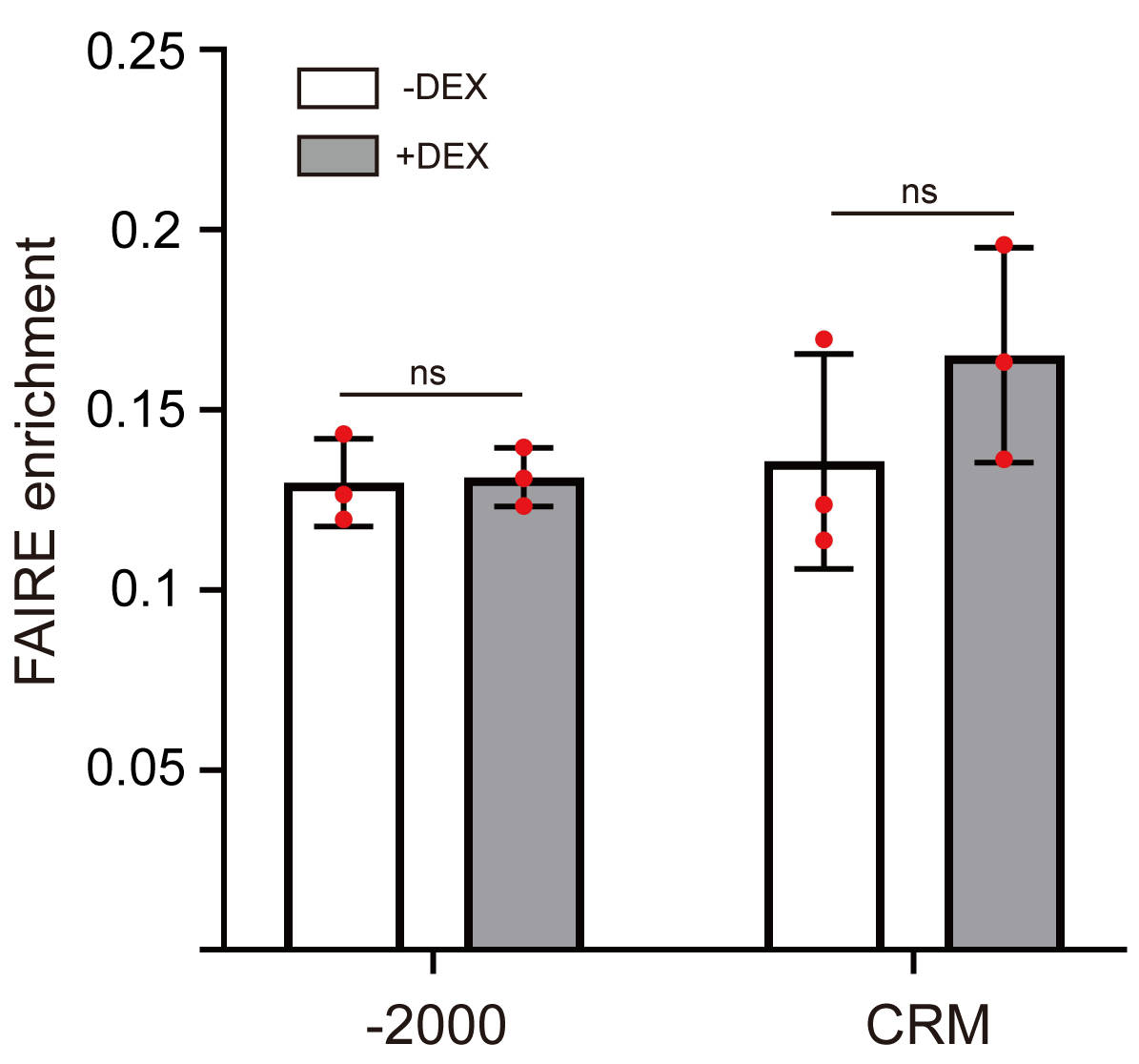

Supplement: S18 Fig — FAIRE assays were performed, using 35S::DRN-GR lines (10-day-old seedlings) inducted with 15 μM DEX for 3 h, to check the chromatin state at CRM region, downstream of CLV3. The upstream −2,000 site was used as the negative control. Data represent means ± SDs from 3 biological replicates. ns, no significant difference; Student’s t test. The experiments were independently performed 2 times with similar results. The data underlying this figure can be found in S1 Data. (TIF) [file pbio.3002878.s019.tif]

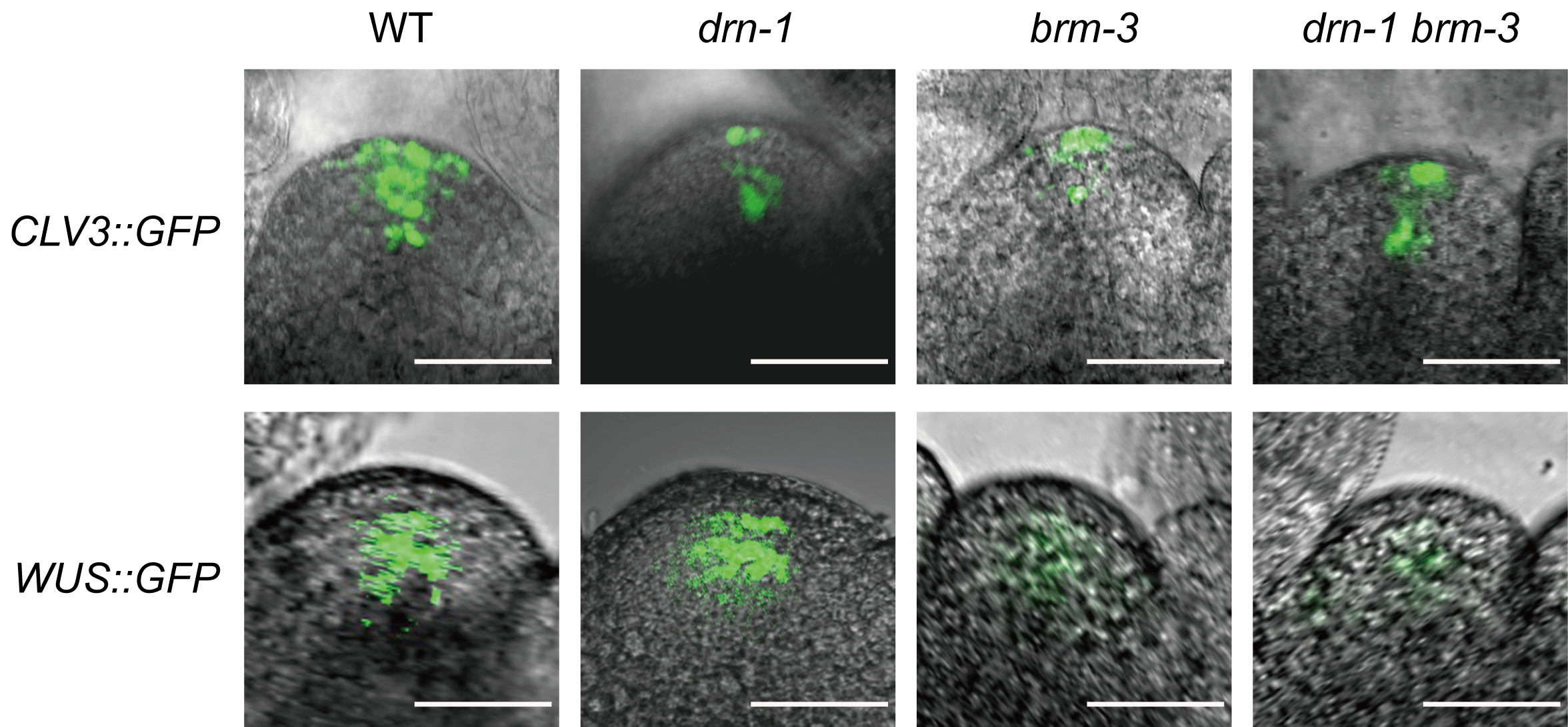

Supplement: S19 Fig — CLV3::GFP and WUS::GFP lines were crossed with drn-1, brm-3, and drn-1 brm-3, respectively. CLV3 and WUS expression was checked in these mutants by fluorescence. Six apices were analyzed in each line. Scale bars, 50 μm. The experiments were independently performed 2 times with similar results. (TIF) [file pbio.3002878.s020.tif]

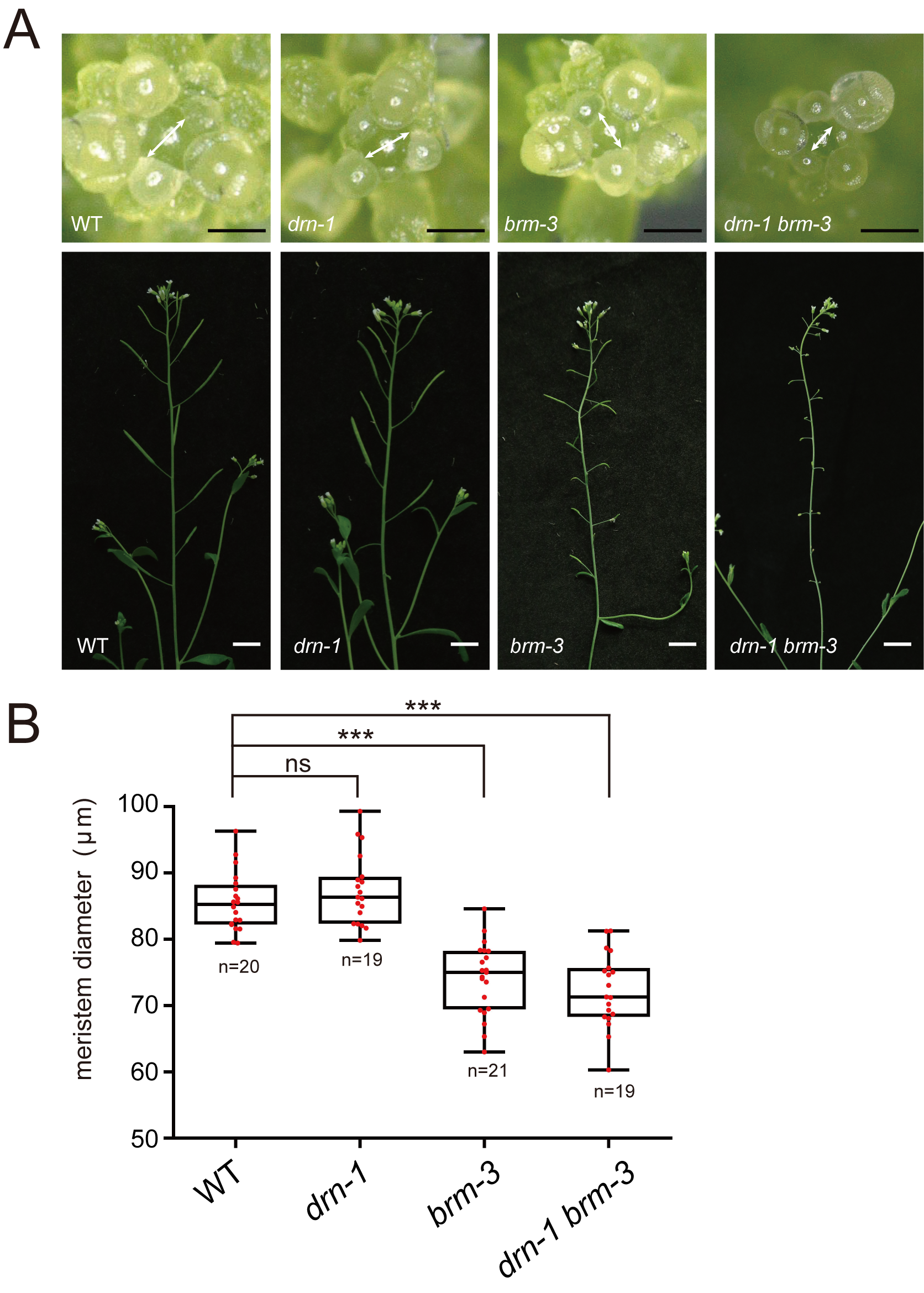

Supplement: S20 Fig — (A) The phenotypes of indicated mutants, including the shoots and SAMs, are shown. The white arrows indicate the diameter of SAMs. Black scale bars, 100 μm. White scale bars, 5 mm. (B) The SAM sizes of plants in A were analyzed. Black bars, highest and lowest values; box, median 50%; black line in the box, median. ***P < 0.001; ns, no significant difference; Student’s t test. The experiments were independently performed 2 times with similar results. The data underlying this figure can be found in S1 Data. (TIF) [file pbio.3002878.s021.tif]

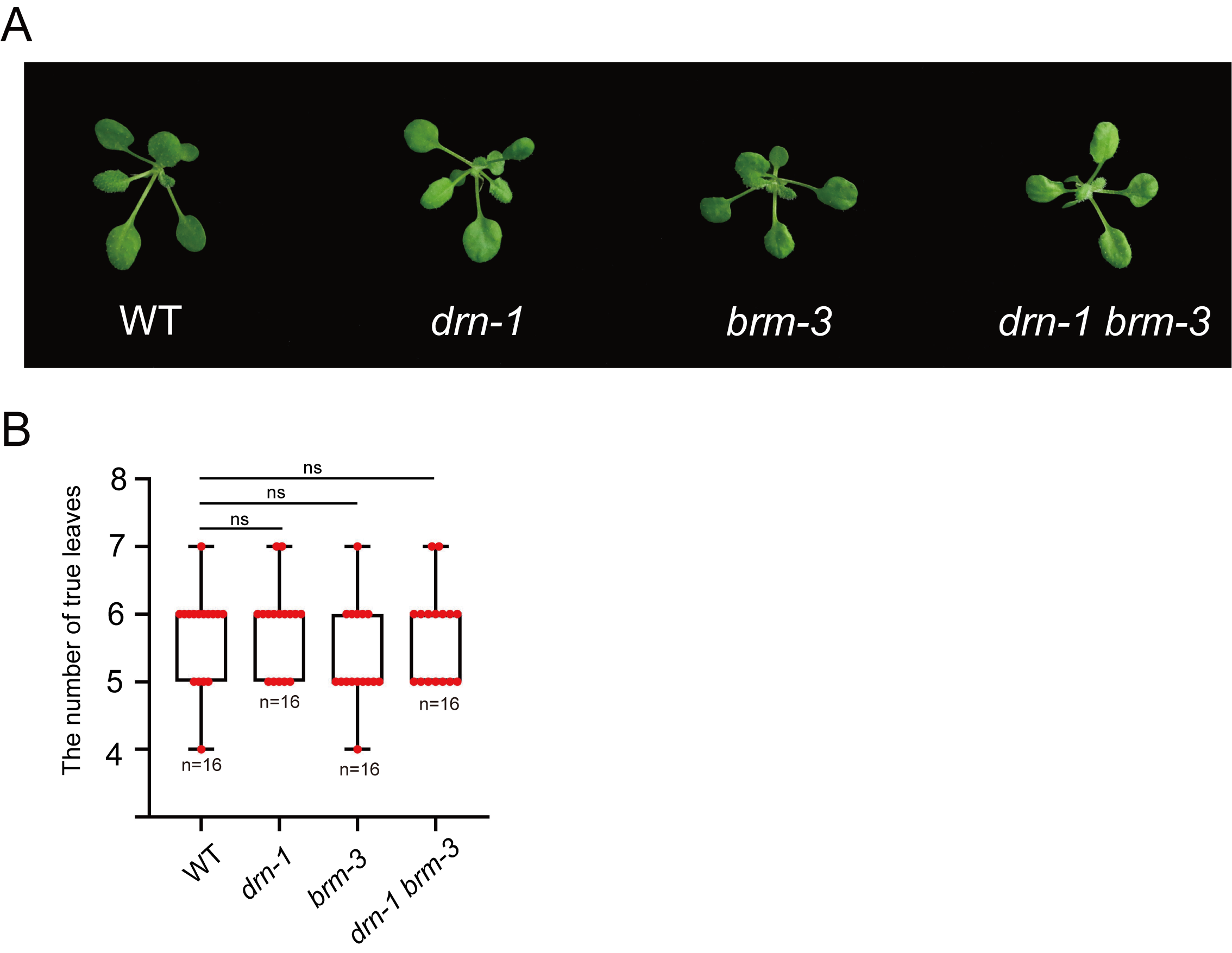

Supplement: S21 Fig — The number of true leaves was analyzed in WT, drn-1, brm-3, and drn-1 brm-3, using 14-day-old seedlings. Black bars, highest and lowest values; box, median 50%. ns, no significant difference; Student’s t test. The experiments were independently performed 2 times with similar results. The data underlying this figure can be found in S1 Data. (TIF) [file pbio.3002878.s022.tif]

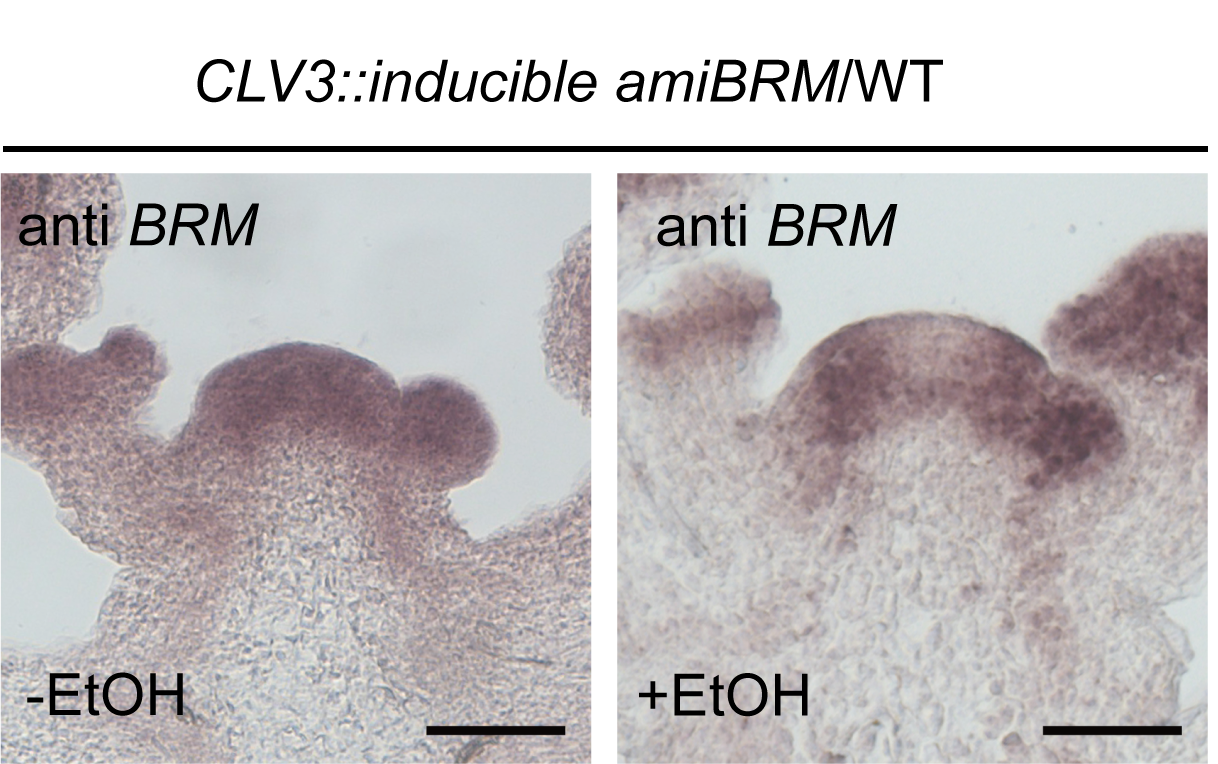

Supplement: S22 Fig — BRM mRNAs were detected by RNA in situ hybridization in SAMs of CLV3::inducible amiBRM plants after 1% ethanol induction for 24 h during the reproductive stage. Scale bars, 50 μm. Ten apices were analyzed in each group. The experiments were independently performed 2 times with similar results. (TIF) [file pbio.3002878.s023.tif]

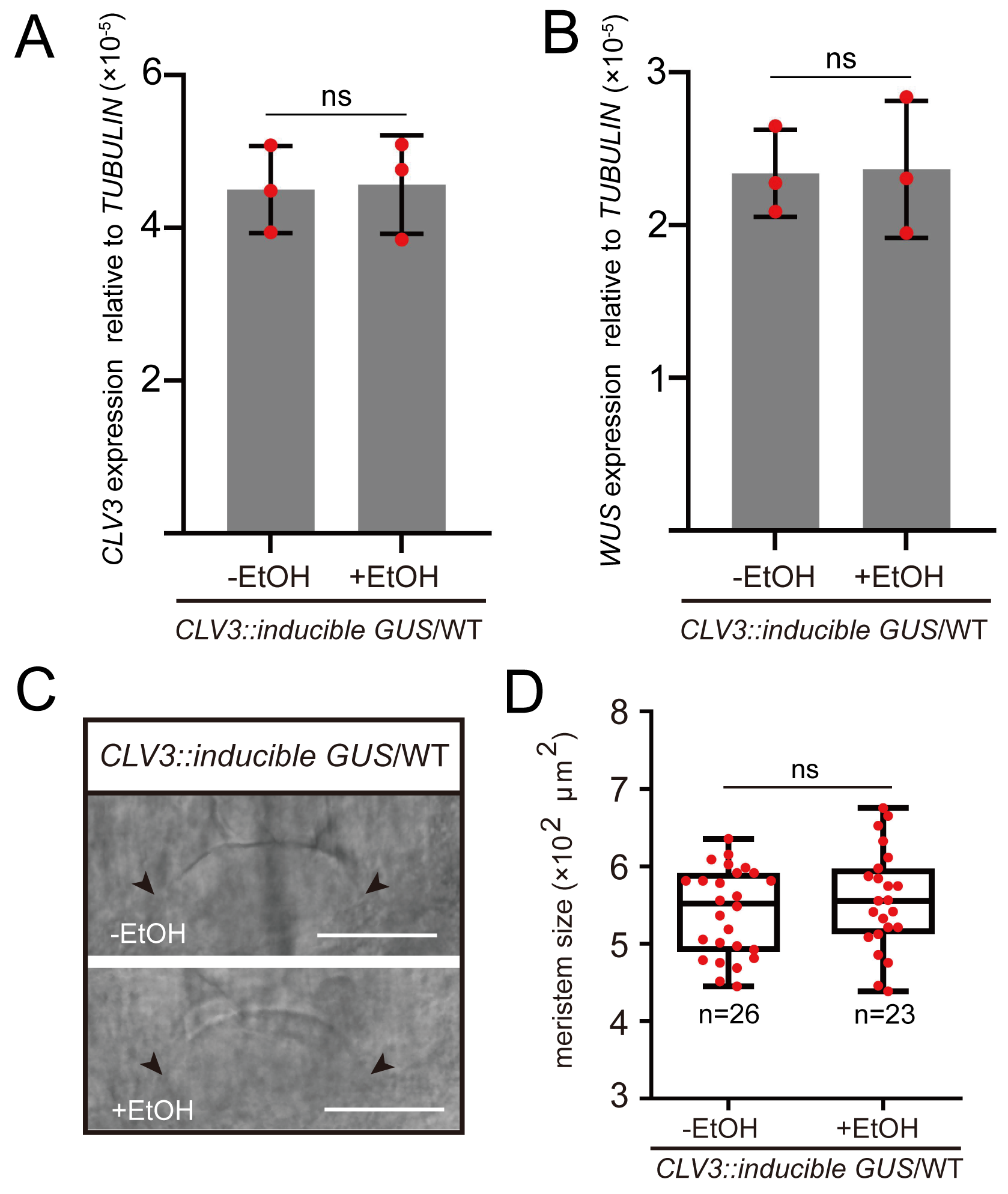

Supplement: S23 Fig — (A and B) CLV3::inducible GUS/WT lines (14-day-old seedlings), as the negative control of Fig 6A and 6B, were used for EtOH induction. qRT-PCR was performed to test CLV3 and WUS expression. Data represent means ± SDs from 3 biological replicates. (C and D) The SAM sizes of CLV3::inducible GUS/WT lines (14-day-old seedlings) with EtOH induction were analyzed, as the negative control of Fig 6C and 6D. The black arrows indicate the boundaries of SAMs. Scale bars, 50 μm. Black bars, highest and lowest values; box, median 50%; black line in the box, median. ns, no significant difference; Student’s t test. All experiments were independently performed 2 times with similar results. The data underlying this figure can be found in S1 Data. (TIF) [file pbio.3002878.s024.tif]
