# Supplementary material for: DRN facilitates WUS transcriptional regulatory activity by chromatin remodeling to regulate shoot stem cell homeostasis in Arabidopsis
Source: PLoS Biol. 2024 Nov 8;22(11):e3002878. doi: 10.1371/journal.pbio.3002878 (PMC11548754; doi:10.1371/journal.pbio.3002878)

Figure 1C

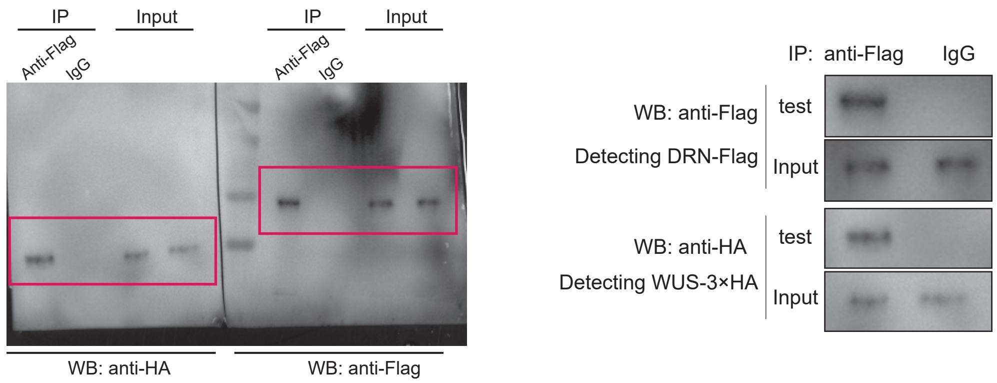

Co-IP in *Arabidopsis*.
35S::DRN-Flag and *35S::WUS-3×HA* were transformed into *Arabidopsis* protoplasts.
Anti-Flag antibody was used for IP.
IgG was used for IP as the negative control.

Figure 1C repeat

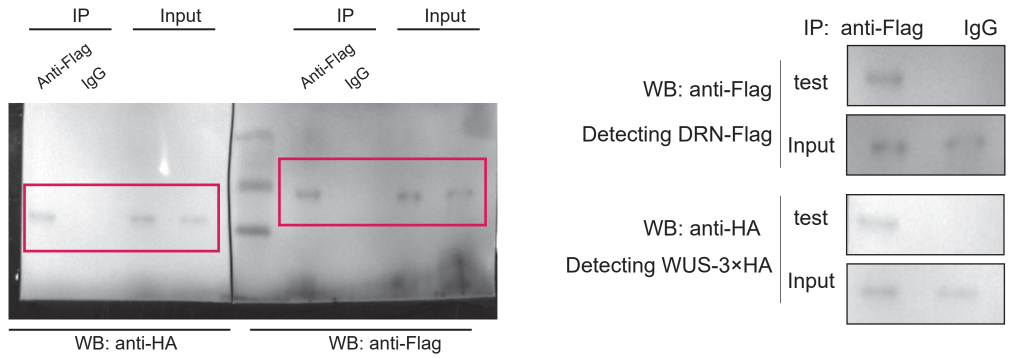

Figure 1D

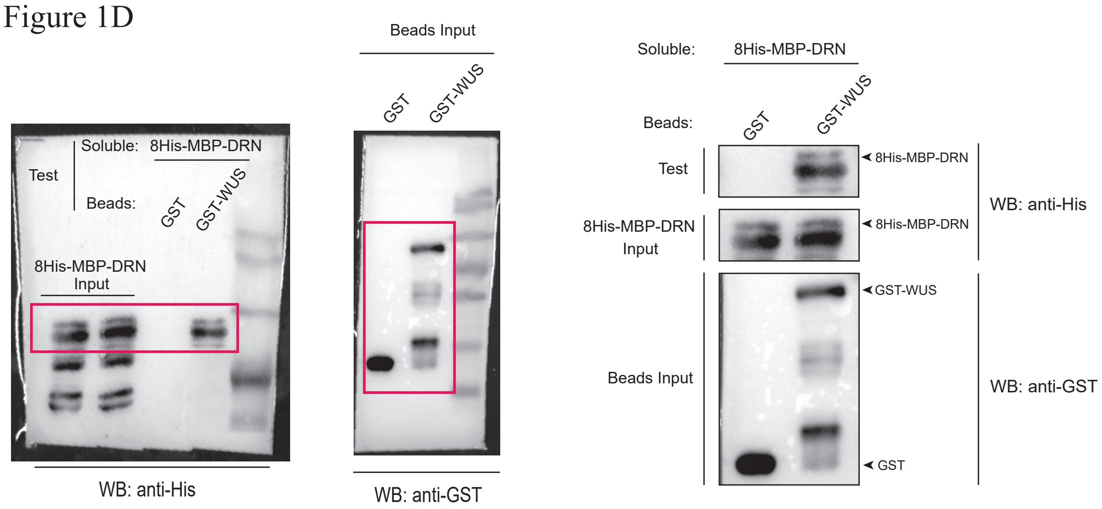

Pull-down shows the interaction of DRN and WUS directly.
Anti-His and anti-GST antibodies were used for immunoblot analysis.

Figure 1D repeat

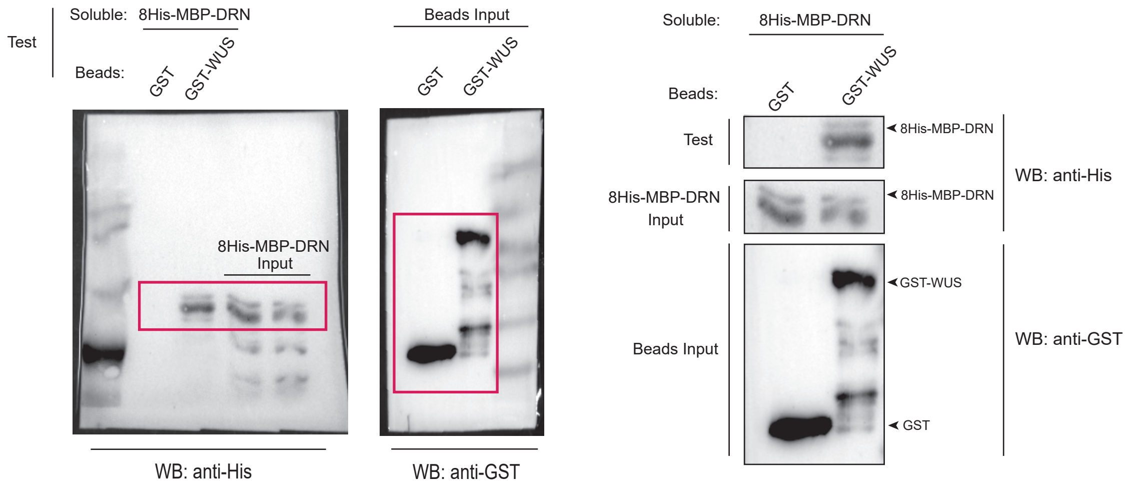

Figure 1F

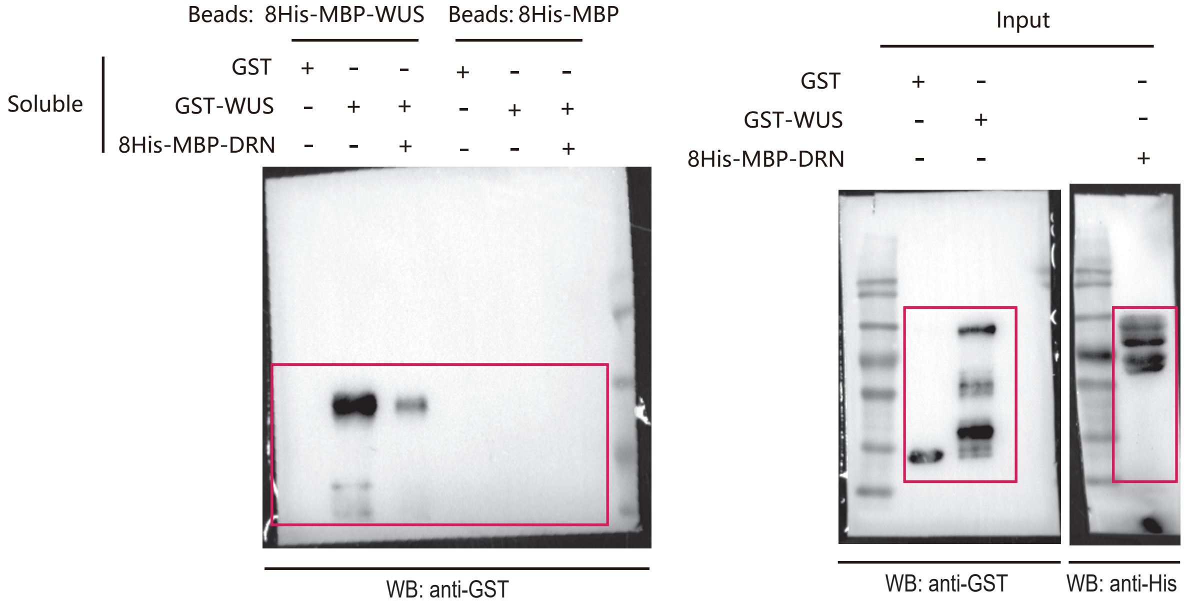

Pull-down assays were used to determine the interruption of WUS homodimer by DRN. The binding group demonstrates the interaction of WUS-WUS, which is compromised by introducing 8His-MBP-DRN.

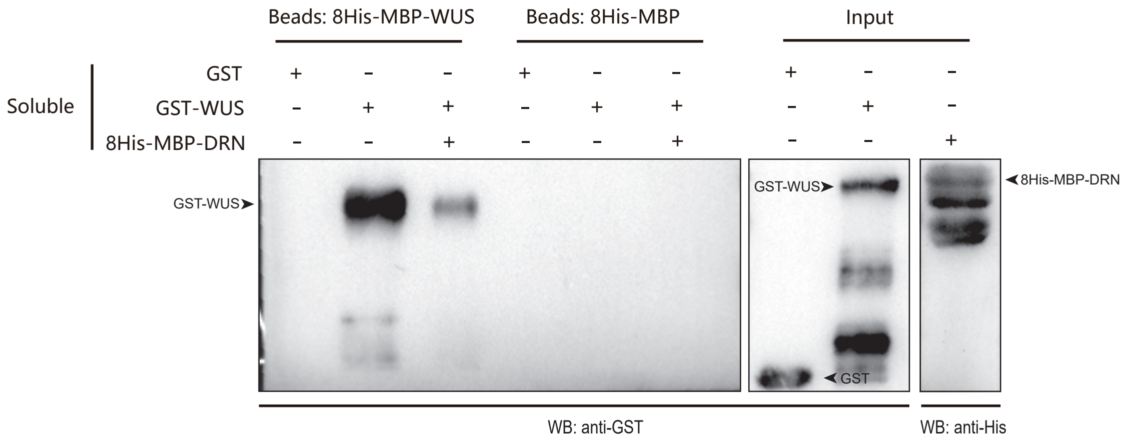

Figure 1F repeat

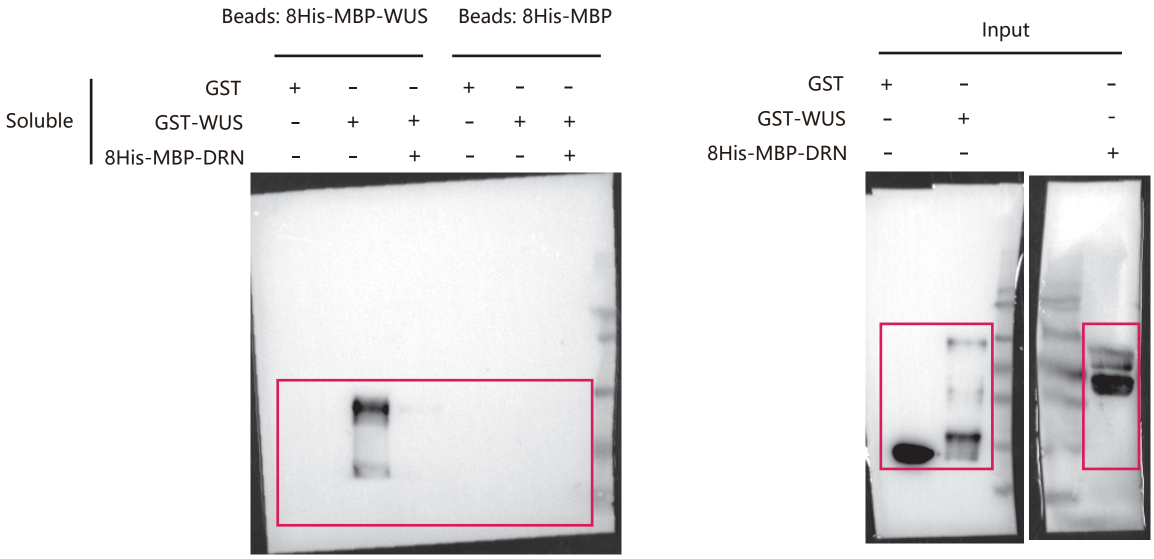

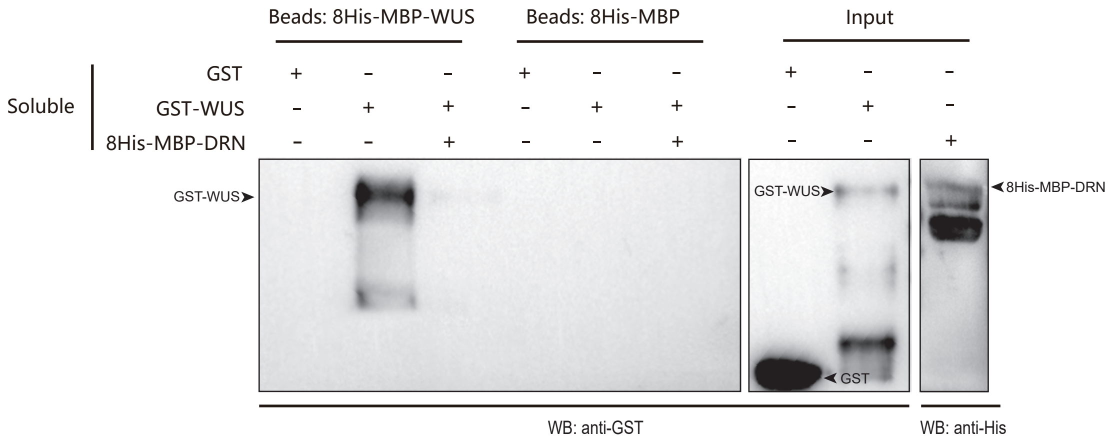

Figure 2B

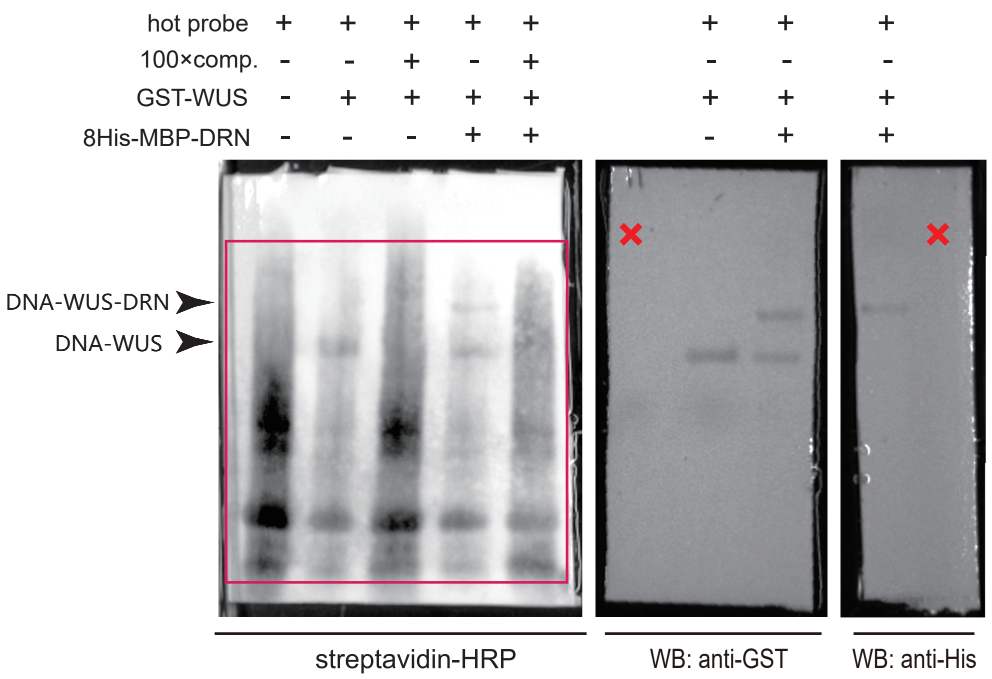

EMSAs results show the direct binding of WUS-DRN and the *CLV3* promoter fragment enriched in ChIP. The black arrows indicate protein-DNA complexes. The western blot analyses were performed to determine GST-WUS and 8His-MBP-DRN proteins in shift and super-shift.

Figure 2B repeat

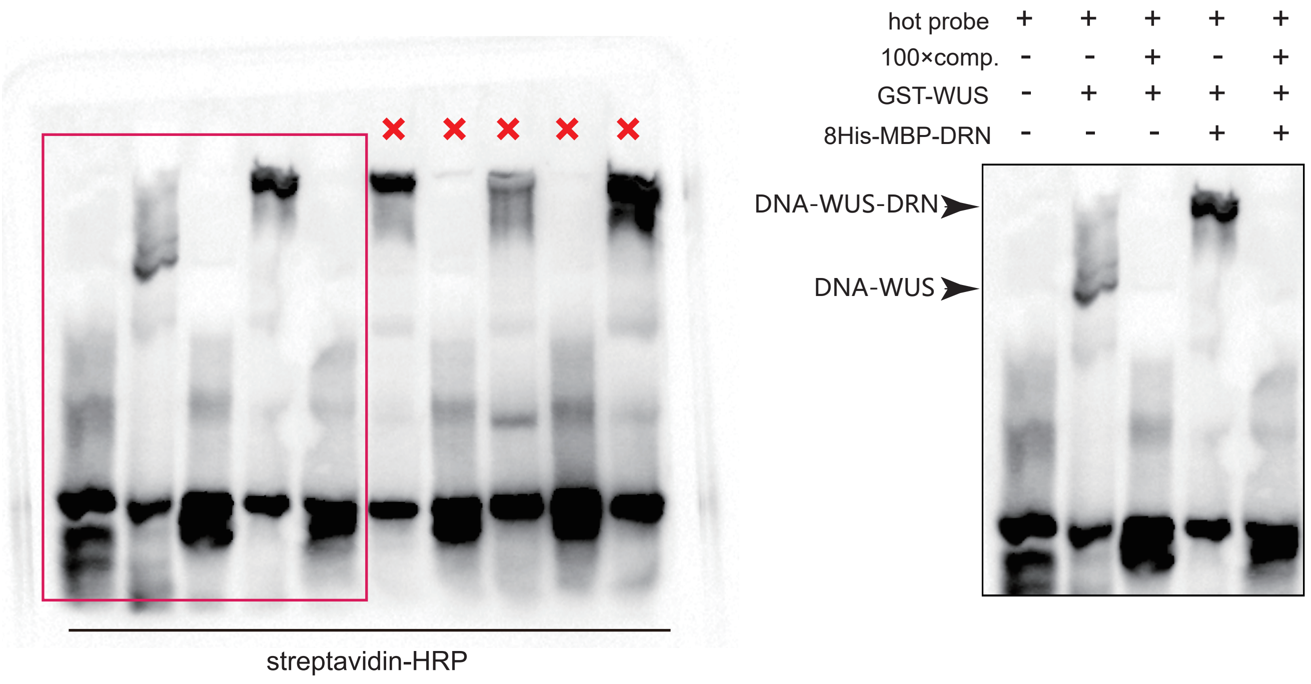

Figure 3C

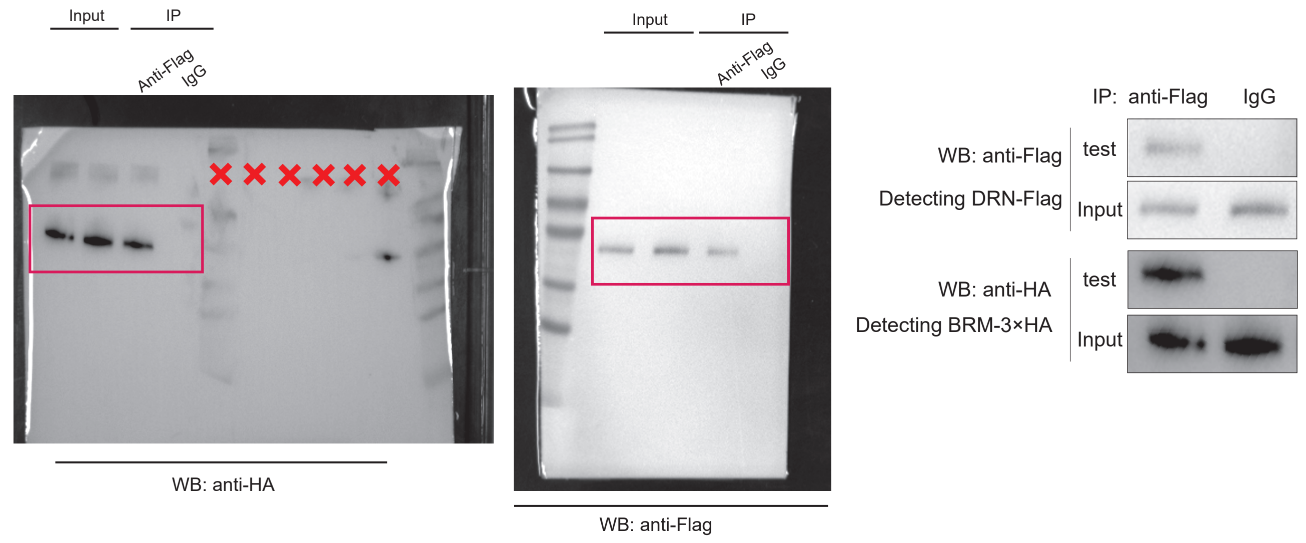

Co-IP in *Arabidopsis*.
35S::DRN-Flag and *35S::BRM-3×HA* were transformed into *Arabidopsis* protoplasts.
Anti-Flag antibody was used for IP.
IgG was used for IP as the negative control.

Figure 3C repeat

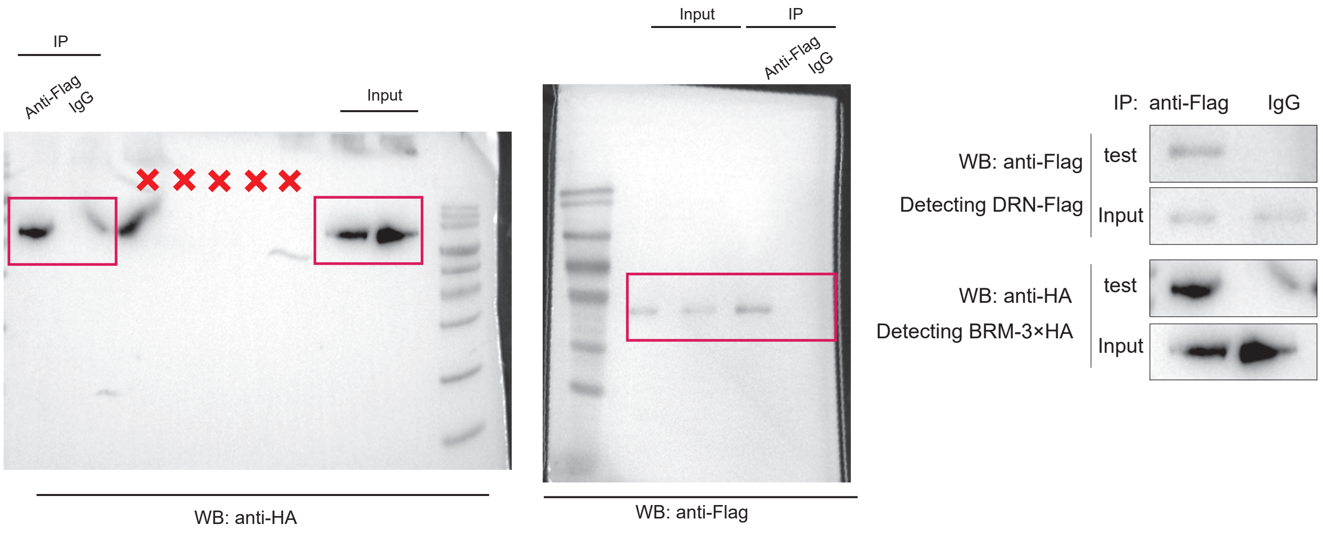

Figure 3D

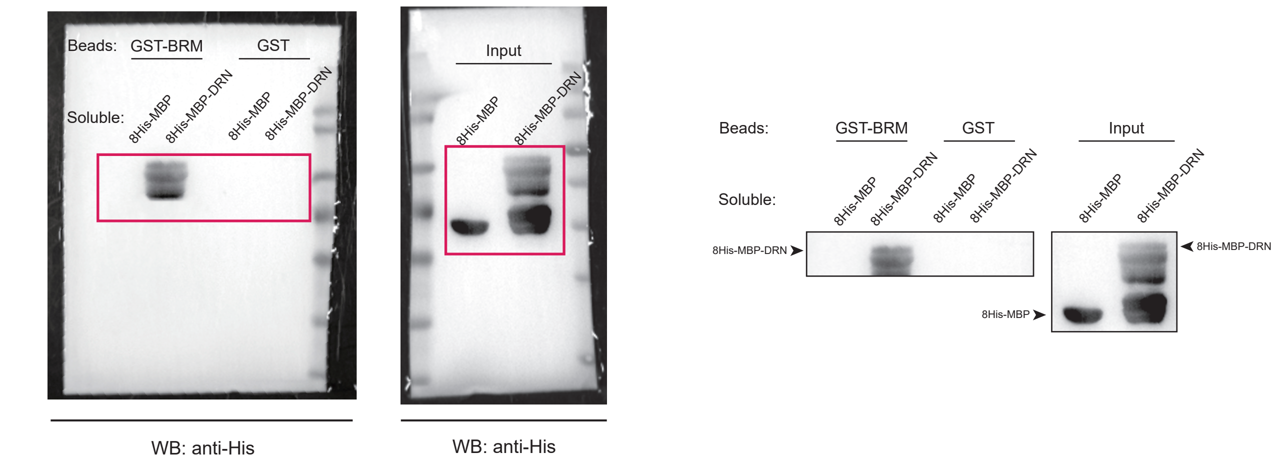

Pull-down shows the interaction of DRN and BRM directly.
Anti-His and anti-GST antibodies were used for immunoblot analysis.

Figure 3D repeat

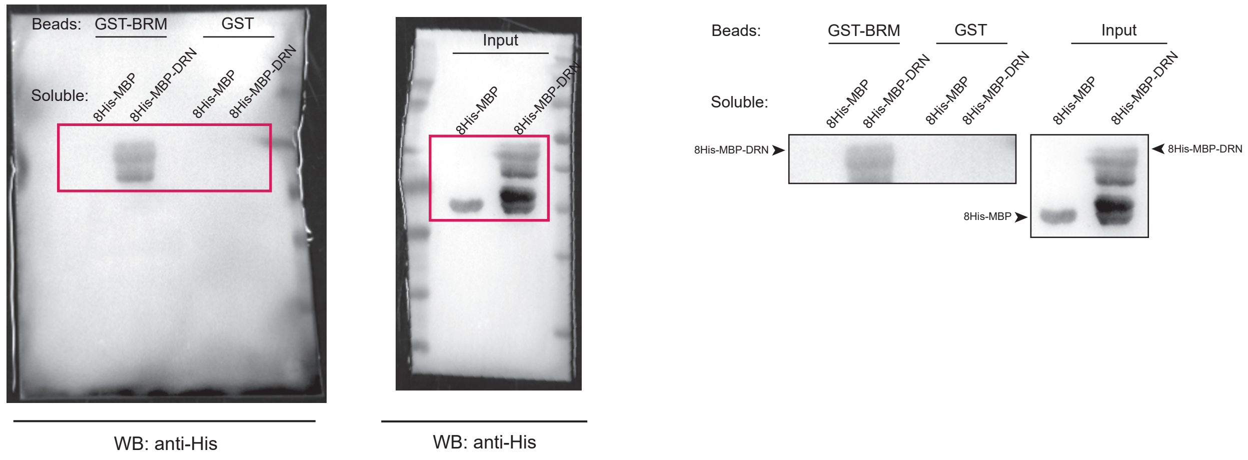

Supplement: S1 Raw Images — Raw gels of co-IP, pull-down, and EMSA assays in this study. The gels used in figures and the repetitive results are included in this file. (PDF) [file pbio.3002878.s026.pdf]
